# A SBM-DEA based performance evaluation and optimization for social organizations participating in community and home-based elderly care services

**DOI:** 10.1371/journal.pone.0248474

**Published:** 2021-03-17

**Authors:** Qiuhu Shao, Jingfeng Yuan, Jin Lin, Wei Huang, Junwei Ma, Hongxing Ding

**Affiliations:** 1 Department of Construction and Real Estate, School of Civil Engineering, Southeast University, Nanjing, P. R. China; 2 Nanjing Municipal Education Bureau, Nanjing, P. R. China; 3 School of Civil Engineering, Sanjiang University, Nanjing, P. R. China; University of Tehran, ISLAMIC REPUBLIC OF IRAN

## Abstract

The community and home-based elderly care service system has been proved an effective pattern to mitigate the elderly care dilemma under the background of accelerating aging in China. In particular, the participation of social organizations in community and home-based elderly care service has powerfully fueled the multi-supply of elderly care. As the industry of the elderly care service is in the ascendant, the management lags behind, resulting in the waste of significant social resources. Therefore, performance evaluation is proposed to resolve this problem. However, a systematic framework for evaluating performance of community and home-based elderly care service centers (CECSCs) is absent. To overcome this limitation, the SBM-DEA model is introduced in this paper to evaluate the performance of CECSCs. 186 social organizations in Nanjing were employed as an empirical study to develop the systematic framework for performance evaluation. Through holistic analysis of previous studies and interviews with experts, a systematic framework with 33 indicators of six dimensions (i.e., financial management, hardware facilities, team building, service management, service object and organization construction) was developed. Then, Sensitivity Analysis is used to screen the direction of performance optimization and specific suggestions were put forward for government, industrial associations and CECSCs to implement. The empirical study shows the proposed framework using SBM-DEA and sensitivity analysis is viable for conducting performance evaluation and improvement of CECSCs, which is conducive to the sustainable development of CECSCs.

## 1. Introduction

Globally, the aging population is increasing at an astonishing speed. According to World Health Organization, the proportion of the world’s population over 60 years old is predicted to rise from 12% to 22% during the period of 2015–2050 [1]. In particular, the population aged above 60 in China is expected to exceed 30% by 2050, which is obviously higher than the world average [2]. As a result, a corresponding number of special care for the elderly will be needed. Since the elders in China are keen to uphold traditional filial piety, they are more willing to choose traditional family care [3]. In response to the urgent needs for more elderly care, eldercare institutions have already undergone significant growth in number and size which provide an alternative for empty-nesters and disabled elders [4]. However, a series of problems in the existing eldercare institutions are apparent, such as ever-increasing operation costs, insufficient provision and inaccessibility [4, 5]. In addition, the community elderly care mainly provides family-oriented services for the elderly, such as life consulting services, mental care, primary and preventive healthcare as well as organizing sports activities. Community elderly care, which can narrow the growing gap between care needs and provision, will become the important trends in China [3].

To gear to the social development, it requires that home-based elderly care be prioritized, and correspondingly, more elderly healthcare facilities should be provided in communities. Meanwhile, the Chinese government is altering its role as direct provider to regulator and policy maker for all social welfare and encourages greater participation of social forces to fill the vacuum [6]. Given its nonprofit nature and commitment to service, social organizations can be viewed as the best choice to bridge the shortfall of elderly care services. In essence, social organizations are responsible to make full advantage of the donations and fund collected to cater for the citizens. Therefore, social organizations play a more important role in elderly care service delivery and emerge as the answer to the failure of China’s welfare economy.

Contemporarily, it is an inevitable trend for social organizations to become the main body of community and home-based elderly care [7]. The participation of social organizations in community and home-based elderly care can not only tackle practical problems, but also ensure the long-term development. However, there are still some problems in its development process, such as the imperfect organizational structure and government supervision system, the unsound service system, the lack of policy support, the single source of funding and the low purchasing process standardization [8–10]. These problems lead to the low-quality of community and home-based elderly care involving social organizations and cannot give full play to the advantages of solving the pension plight.

The study of community and home-based elderly care involving social organizations has begun to catch the attention of academia for the moment in China, but unfortunately, the depth and breadth of its research is far from enough. The existing research is given priority to case description, and focuses on the analysis of government purchasing policy, the choice of purchasing patterns, the problems and countermeasures of community and home-based elderly care involving social organizations [11–13]. However, few scholars pay close attention to the performance management and evaluation which is urgently needed for the above-mentioned problems. Hence, It is vital to study the performance of social organizations involved in community and home-based elderly care and evaluate the performance of them, which would not only help social organization to optimize itself and improve efficiency, but also be conducive to the government departments to strengthen the management, thereby promoting the structural optimization of the elderly care system.

Performance refers to the effective output of an organization at different levels to achieve its goals [14]. Peter Drucker indicated that performance management should be taken seriously by every organization [15]. Taylor established Scientific Management Theory in 1891, which marked the establishment of enterprise performance evaluation system [16]. Subsequently, the research scope of performance management was gradually expanded, from the first enterprise performance management to the later organization and individual performance management, and then to the government performance management. In recent years, with the methodology of ABC cost accounting, AHP, FAHP, DEA, Grey Relation Analysis, Social Network Analysis and other multi-disciplinary method, performance evaluation and management has formed a rather mature system [17–19].

The main question of this research is to propose a new approach to evaluate and optimize performance of social organizations involved in community and home-based elderly care. In the concrete practice of China, social organizations are known as community and home-based elderly care service centers (CECSCs). In this study, the index system is firstly constructed based on the social benefits of CECSCs. Then SBM-DEA is used to evaluate the performance of the CECSCs and Sensitivity analysis is utilized to screen the direction of performance optimization. The data of different levels of CECSCs are compared and analyzed. Finally, Specific development suggestions were put forward for different participants.

The main contributions of this study can be summarized as follows. This study focuses on the performance evaluation and optimization of CECSCs, and fills the gap of relevant research. Meanwhile, this study provides several directions for optimizing the performance of CECSCs, which is conducive to promoting the development of home-based care services in regional China.

The remaining part of this paper is organized as follows. In Section 2, related literatures are reviewed. Section 3 introduces the proposed method of framework analysis. The proposed method is applied to explore the performance of the CECSCs in Nanjing in Section 4. Further discussion and suggestion are proposed in section 5. Conclusions are provided in Section 6.

## 2. Literature review

### 2.1 Social organizations involved in community and home-based elderly care services

Social organizations are institutions whose aims are not necessarily to provide shareholders a return on their investment but to achieve social outcomes through various projects and activities [20, 21]. Social organizations may appear in public under different names, such as non-governmental, non-profit organizations, voluntary, humanitarian, philanthropic, independent and civil society organizations. For the purposes of our research, we will use the term social organizations [22]. Social organizations fulfil vital functions particularly for humanitarian-oriented activities, which are not addressed by the government and market institutions, such as health, non-formal education, relief and capacity building, protect wildlife, labor inclusion, etc. [21, 23, 24]. They are concerned with the problems of marginalization within society, so the role of these organizations in society has been essential, not only at national, but also at an international level [25].

Social organizations have drawn great attention from the public and academic, especially as suppliers of welfare provision, promoters of active citizenship, and guardians of the common and greater good in society through their special characteristics and values [26]. In co-production and co-creation theories [27, 28], social organizations engagement in community and home-based elderly care aims at achieving a public benefit which allows to increase the volume and quality of elderly care services and promote equality in the consumption of elderly care services and customer satisfaction. In China, rapid aging, a lack of public support for frail elders, and the failure to meet the needs of the old have led to including nonprofits as providers in the welfare for the elderly [6]. Participation of social organizations can not only increase the efficiency of the service but also have a positive effect on the democratic nature of service delivery. CECSCs have been set up in China to play the role of social organizations, which integrate social resources and provide services for the elderly.

Meanwhile, there are many weaknesses in social organizations, which may lead partnerships in the production of public services to fail. Because social organizations are not profit-oriented, they have to use specific operational models and rely on multiple resource providers to implement their operations [29], such as exemption from paying taxes [30]. However, recent studies on the sustainability and accountability of social organizations have raised significant concerns that social organizations are philanthropic uncertainty (inadequacy of owned and attracted resources, or relying heavily on government funding), philanthropic particularism (focusing on particular client groups and geographical limiting), philanthropic distrust (abusing and misconducting of religious charity funds), philanthropic paternalism (influence of donors’ interests on the mission and perceiving the contribution to the organizations entitlement, not as a right), philanthropic amateurism (problems with attracting professional workers) [24, 31].

Therefore, social organizations are facing growing pressure to performance-oriented, when they mobilize the endowment of resources and deliver services [32, 33]. Since Social organizations strive to the goal of public profit maximization [34, 35], it is necessary to secure development of such organizations not just by an increase of subsidies, but mainly by proper managing systems and radical elimination of what is not bringing any value [36]. Performance management of social organizations are essential for decision-making for governments, funders, clients, donors and all stakeholders [37]. Performance measurement helps to clarify expectations, promote consistency, provide risk signals, allow precision and objective forecasts, promote motivation and improve solutions to problems, improve accountability and increase objectivity [32]. Taking into account that many social organizations need to create trust and confidence, it is the multiplication of benefits from performance measurement as well.

### 2.2 Performance management and evaluation

Performance management, which is defined as a closed loop control system that deploys policy and strategy, and obtains feedback from various levels to manage the performance of the system [38], has been widely adopted by a wide range of industries [4, 39]. All organizations exist with high-level performance [40]. As such, performance management is positively related to strategic performance of organizations [41]. In the performance management system for organizations, the managers and implementers set objectives, measure and recapitulate how these objectives are met, give good performance reward and support continuous improvement [42]. Critical issues of performance management are the definition of objectives, measurement of objective achievement in terms of meeting all stakeholder requirements, the process of objectives attained and operation management [43]. To achieve performance management, managers or decision makers need to know which performance they seek. Hence, performance measurement constitutes an integral part of the performance management.

The success of a social organization depends on a plethora of factors, in which performance measurement is of paramount significance to ensure the outcome achieved [44–46]. Performance management and measurement are used to obtain a performance score by measuring relevant performance indicators, proceed analysis based on this score and improve performance continually. Thus, to identify the measuring and managing performance indicators is at the heart of performance management and measurement [47]. Prior studies have conducted studies about the financial indicators of social organizations [48, 49]. However, Social organizations are mission-driven (rather than profit-driven) organizations and we cannot simply use financial indicators to measure the overall performance of social organizations [50]. Moreover, it should be noted that non-profit social organizations have begun to include outcomes as metrics of performance [51]. Many studies implement the outcome-based evaluations to measure performance of social organizations by the approach of DEA, which considers three dimensions: service quality, effectiveness and efficiency [52].

Performance evaluation is the process of determining the efficiency and/or effectiveness of past action, which has been widely adopted to measure the performance of social organizations [53]. The commonly-used methods for performance measurement include ratio analysis, regression analysis, multiple-criteria decision making analysis, AHP, BSC, the Delphi method, cost-benefit analysis, fuzzy comprehensive evaluation, and data envelopment analysis(DEA) [54–58]. In effect, healthcare input and output are intractable to quantify and are easily affected by government polices [54]. Therefore, the functions between input and output show an insignificant relationship, leading to difficulty in evaluating performance of social organizations. Unlike other methods mentioned above, which need to judge indicator weight subjectively and make data dimensionless, the DEA can resolve qualitative problems with quantitative analysis, transform subjective judgments into objective ones, and develop an unbiased weighting or scoring for aggregation [59]. In addition, DEA is a simple and practical multi-criteria evaluation method applied in various categories [60]. Government departments, transportation projects, education institutions and etc., often use DEA in evaluating the operation performance of the institution or unit [61–63]. Thus, the DEA method is more suitable and feasible for this study.

### 2.3 Knowledge gap

The study of social organizations participating in community and home-based elderly care has drawn the attention of researchers. However, prior studies mainly focused on the analysis of government purchase conversion [64], purchase mode selection [65], problems and countermeasures [66]. Few scholars have paid attention to the performance management of social organizations participating in home-based elderly care services.

The current academic research on the performance management is relatively mature. The performance management of social organizations has gradually become a hot research field. But the previous study on social organizations are mostly referred to the concept of social organizations in a narrow sense, that is, private non-enterprise units and foundations, as well as institutions with strong administrative nature such as universities [67], healthcare [41], sports organizations [68]. There are few studies on the performance evaluation of social organizations participating in community and home-based elderly care, which provides the corresponding space for this study.

## 3. Materials and methods

### 3.1 Model selection

As a social organization, CECSC integrates massive social resources and provides social public services, which are relatively homogeneous and characterized by obvious uncertainty between input and output [69, 70]. Therefore, this study chooses DEA, which has a strong applicability to the relative efficiency of homogeneous decision-making units (DMUs) with multiple inputs and multiple outputs, and also can effectively estimate the production frontier [71].

DEA model is an input and output analysis method based on relative efficiency proposed by Charnes, Cooper and Rhodes in 1978 [72]. Particularly, its derived non-parametric models, such as Charnes-Cooper-Rhoder(CCR) and Banker-Charnes-Cooper(BCC), have been widely employed to measure the relative efficiency of the same type of DMUs with multiple input and output indicators [73]. However, CCR and BCC model are typical radial model, which ignore the slack adjustment. Besides, these model cannot distinguish the efficient DMUs further [74]. Based on the CCR model, Tone(2001) constructed a non-radial slack-based measurement(SBM) model in which all slacks measures were incorporated into the objective function via a scalar method [75]. Therefore, SBM-DEA model can eliminate the deviation of efficiency measurement caused by the difference of radial selection, thereby obtaining more objective and accurate efficiency.

The purpose of this study is to comprehensively and systematically evaluate the management performance of CECSCs. At present, CECSCs in China are featured with public welfare, and since they are just starting out, the government has invested a large amount of funds and resources to maintain their normal operation, so the investment is astonishingly large, and it is difficult to quantify the real efficiency of the investment. Therefore, the output-oriented SBM-DEA model is adopted in this study.

Suppose that there are *n* DMUs, which refer to CECSCs in this study. Each DMU has *m* input indicators and *s* output indicators *y*. *B*_*j*_ is the *j*-th DMU, where: *j* = 1, 2, ……, *n*, [*x*_*ij*_] is *m*×1 input indicators of *B*_*j*_, where: *i* = 1, 2, ……, *m*, [*y*_*rj*_] is *s*×1 output indicators of *B*_*j*_, where: r = 1, 2, ……, *s*. The relative efficiency value of the *j*_*0*_-th DMU is expressed as *h*_*j0*_. The output-oriented SBM-DEA model with variable returns to scale assumption is as follows:

$$\mathrm{Min}h_{j0}=\theta$$

$$s.t\{\begin{array}{c} \sum_{j=1}^{n}\lambda_{j}x_{ij}\leq \theta x_{ij0},i=1,\ldots .,m\\ \sum_{j=1}^{n}\lambda_{j}y_{rj}\geq y_{ij},r=1,\ldots .,s\\ \sum_{j=1}^{n}\lambda_{j}=1,\lambda_{j}\geq 0,j=1,\ldots ..,n \end{array}$$

In this formula, the efficiency value of *j*-th is expressed as *θ*. *λ*_*j*_ represents nonnegative vector. When *θ* = 1, the DMU is only considered efficient, otherwise, the DMU is inefficient and there is room for improvement. In this study, MaxDEA Basic 6.13 is used as the software for SBM-DEA model analysis.

After evaluating the performance of CECSCs through SBM-DEA, Sensitivity Analysis (SA) method is also employed in this study to design the path of performance optimization. SA is a common uncertainty analysis method in economic decision-making. It analyzes and predicts the influence of various uncertain factors on the economic effect of the program, finds out the factors that have greater influence on the economic effect of the program, or called sensitivity factors, and determines their influence degree [76]. The quantitative analysis of SA provides scientific data to identify the optimization direction and range of CECSCs, and realizes the effective practice of scientific and refined management in the field of performance optimization.

### 3.2 Indicator system construction

Scientific and reasonable indicator selection is the foundation of evaluation. Based on the extensive literature review and field interviews, this study constructs a performance evaluation indicator system of CECSCs. 33 indicators were proposed for the performance evaluation and these indicators could be categorized into six dimensions (i.e., financial management, hardware facilities, team building, service management, service object and organization construction) as shown in Table 1.

**Table 1:** Evaluation indicator system of operation performance of CECSCs.

Dimension	Primary Indicators	Secondary Indicators and Description	
A1 Financial management	B1 Capital source	C1 Government funding [77]	The sum of government investment per year
		C2 Donated funding [77]	The sum of donated investment per year
		C3 Other funding [77]	The sum of other investment per year
	B2 General financial position	C4 Operating income [78]	The sum of operating income per year
		C5 Overall financial evaluation [79]	The sum of operating income per year/ The sum of investment per year
A2 Hardware facilities	B3 Operating site	C6 Land Use [80]	The Land ownership of CECSCs
		C7 Size of site [41]	The area of CECSCs
	B4 Operating facility	C8 Number of beds [41]	Number of beds in CECSCs
		C9 Total amount of fixed assets [80]	Total amount of fixed assets of CECSCs
A3 Service management	B5 Service diversification	C10 Types of basic services [81]	Types of basic service, including food, bath, cleaning, emergency, and treatment
		C11 Types of other services [82, 83]	Types of other services
	B6 Quality of services	C12 Quality of basic services [84]	Quality of basic service, including food, bath, cleaning, emergency, and treatment
		C13 Quality of other services [85]	Quality of other services
A4 Team development	B7 Team composition	C14 Number of administrative staff [81]	Number of managers in CECSCs
		C15 Number of staff [81]	Number of staff in CECSCs
		C16 Number of social workers [86]	Number of social workers in CECSCs
		C17 Number of volunteers [87]	Number of volunteers in CECSCs
		C18 Number of other service personnel [81]	Number of other person in CECSCs, such as nurses
	B8 Staff training	C19 Qualification management evaluation [88]	The qualification level of CECSCs
		C20 Training management level [89]	The regular training quantity of CECSCs
		C21 Employee satisfaction level [90]	Job satisfaction of employees in CECSCs
A5 Elderly Care Recipients	B9 Community coverage	C22 Number of old people served [81]	Number of old people that government’s purchased service
		C23 Coverage of social services for the elderly [83]	The ratio of the number of elderly people serving to the number of elderly people in the community
	B10 Customer satisfaction	C24 Elderly satisfaction [91]	Satisfaction with the services provided by the elderly
		C25 Number of complaints [92, 93]	Number of complaints
A6 Organization management	B11 System management	C26 Degree of financial regulations perfection [94]	Evaluating whether there are detailed, clear, operable financial management procedures and regulations
		C27 Reasonable degree of organizational structure [94, 95]	Evaluating whether there are detailed, clear, operable organization management procedures and regulations
		C28 Degree of security system perfection [96]	Evaluating whether there are detailed, clear, operable safety management procedures and regulations
		C29 Degree of reward and punishment system perfection [97, 98]	Evaluating whether there are detailed, clear, operable reward and punishment management procedures and regulations
	B12 Process management	C30 Degree of service process perfection [99]	Evaluating whether there are detailed, clear, operable business management procedures and regulations
		C31 Degree of emergency management perfection [100]	Evaluating whether there are detailed, clear, operable emergency management procedures and regulations
	B13 Feedback mechanism management	C32 Degree of complaint handling perfection [101, 102]	Evaluating whether there are detailed, clear, operable complaint management procedures and regulations
		C33 Degree of Comments and Suggestions handling perfection [102]	Evaluating whether there are detailed, clear, operable Comments and Suggestions management procedures and regulations

The first dimension is financial management. Achievements of performance management of home-based care are mainly reflected in whether the funds such as operator investment, government subsidies, operating income and social donations are effectively utilized to achieve the balance of income and expenditure, and the above investment is transformed into social benefits through operation [77]. Meanwhile, the home-based care itself must have a certain source of funds for its service team training, service facilities construction, service content provision, etc [79]. Therefore, financial management dimension evaluation indicators can be identified from the sources of funds and the financial situation.

The second dimension is hardware facilities since hardware facilities determine the upper limit of the development of home-based care [80]. As a home-based care, the size of the site, the number of beds, the selection and quantity of fixed assets determine the number of service objects and the types of services provided by the home-based elderly care service center [41].

The third dimension is the management of the service. Service is the direct embodiment of the effective output of CECSCs [82, 83]. The type and quality of services provided by community and home-based elderly care not only directly determine the amount of operating income, but also represent the social benefits it can effectively produce [84].

The fourth dimension is team development which indicates that the effective training of CECSCs for employees can promote service level of the whole industry and propel the overall progress of the whole industry, which has great social implications [89]. The team composition of CECSCs is mainly consisted of management personnel, service personnel, social workers, volunteers and other personnel [81, 86, 87]. Therefore, we set up the evaluation index of team development dimension from two aspects of team composition and staff training.

The fifth dimension is elderly care recipients. If an organization aims to progress in the long run, it must have its own faithful elderly care recipients [81]. Different from the enterprise organization, the CECSCs do not pay attention to the absolute number and growth rate of customers at the end-user level, but pay more attention to the social benefits of the operation of CECSCs [91]. Therefore, the end-user satisfaction of CECSCs is closely connected with its mission.

The sixth dimension is organization management which indicates that the standardized operation of an organization is the key to its long-term operation and effective development [94]. Systematization and standardization are helpful to clarify working standards and improve working efficiency [97, 98]. The institutionalization and standardization of an organization can reflect the level of its managers and its management performance [101, 102].

### 3.3 Data source

Attention should be laid on the selection of DMUs to enter the analysis as well as the choice and screening of factors [103]. According to *Evaluation standard of CECSCs in Nanjing (2018 edition)* and *Opinions on carrying out the construction of CECSCs*, the municipal civil affairs bureau and the district civil affairs bureau divided all the CECSCs into five grades in Nanjing. The higher the level of the CECSCs, the better service it will provide for the elderly. There were 509 CECSCs rated 3A or above, among which 27 were rated AAAAA, 53 were rated AAAA and 429 were rated AAA in Nanjing by the end of 2018. As SBM-DEA modeling requests the minimum number of DMUs to be at least triple the number of inputs and outputs included to attain a reasonable level of discrimination [104], 186 CECSCs were taken into account as DMUs in this study. Considering the feasibility of survey and comprehensiveness of data, the selection of DMUs is spread over 11 districts in Nanjing. In order to avoid the heterogeneity problem of DMUs, all of which were from urban areas.

**Fig 1:**
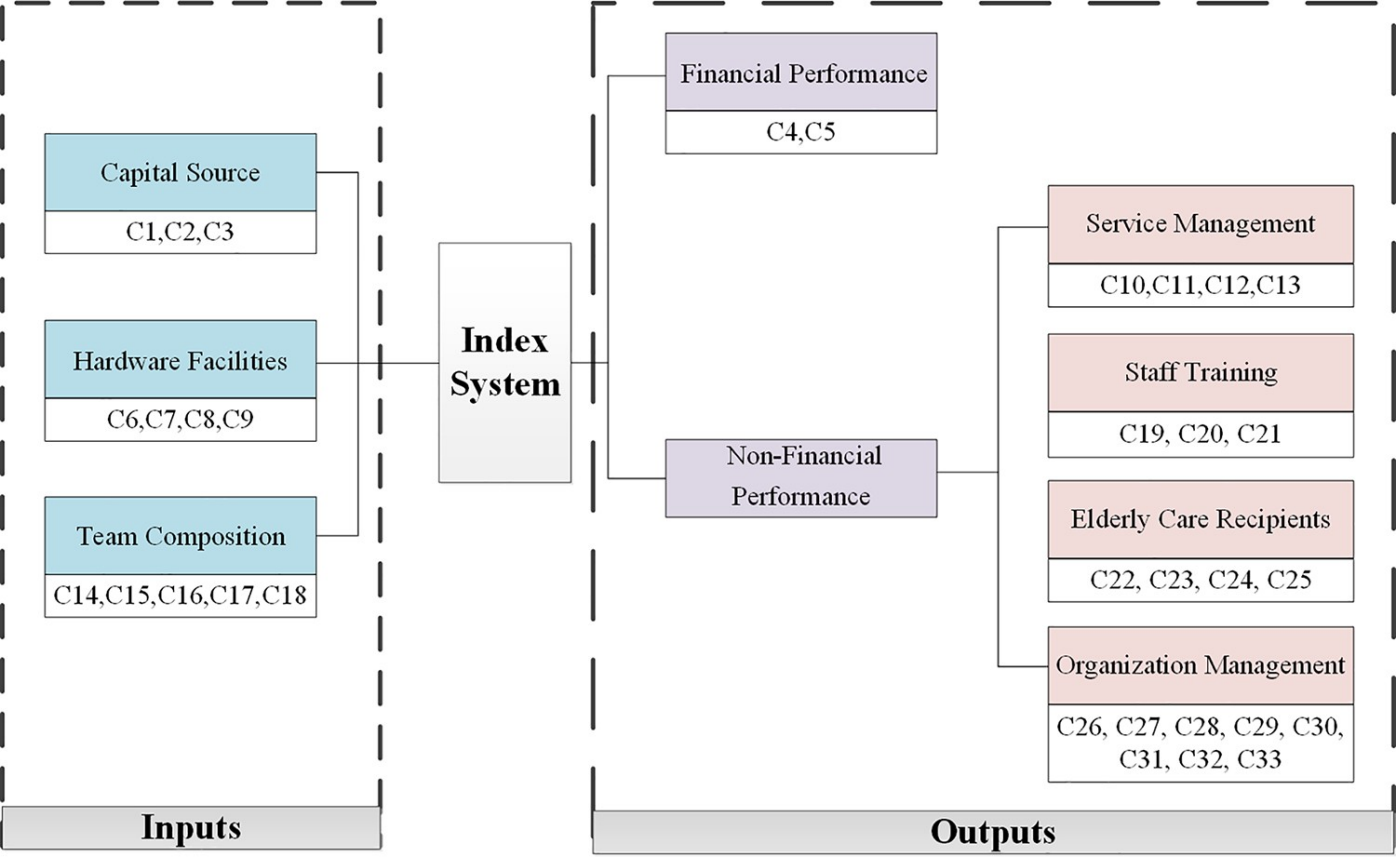
Internal relationship among indicators.

The next step is to determine inputs and outputs for the DMUs to be compared. Normally inputs are defined as resources utilized by DMUs or conditions affecting the performance of DMUs while outputs are outcomes or produced goods and services or benefits generated as a result of operation of DMUs. In order to meet the daily operation of CECSCs, it needs to invest enormous funds, hardware facilities and personnel. Therefore, indicators of capital source (C1, C2, C3), hardware facilities (C6, C7, C8, C9), and team composition (C14, C15, C16, C17, C18) are selected as inputs. Due to the nature of non-profit, it is critical to pay more attention to its social benefit, rather than economic benefit, when evaluating the performance of CECSCs. Therefore, indicators of service management (C10, C11, C12, C13), staff training (C19, C20, C21), elderly care recipients (C22, C23, C24, C25) and organization management (C26, C27, C28, C29, C30, C31, C32, C33) are selected as non-financial performance index in outputs. In addition, as an important indicator to measure whether an organization can survive effectively for a long time and provide services continuously, economic status also occupies a certain proportion in performance evaluation indexes. Therefore, indicators of general financial position (C4, C5) are selected as financial performance index in outputs. Internal relationship among indicators can be seen in Fig 1. The grading criteria is shown in S1 Table.

## 4. Results and discussion

### 4.1 DEA input and output variables

Statistical information of 33 indicators is briefly summarized in Table 2. When the input index appears 0 value, a very small positive number is used to replace 0 value. The raw values of input and output variables are shown in S2 Table.

**Table 2:** Descriptive statistics table.

Variable	Types	Mean	Max	Min	SD
C1	inputs	104803.97	3680000.00	0.00	47543.16
C2	inputs	1391.39	60000.00	0.00	682.09
C3	inputs	7881.72	220000.00	0.00	3560.13
C4	outputs	186622.68	6350051.00	1645.00	92021.09
C5	outputs	0.94	1.00	0.00	0.40
C6	inputs	0.15	2.00	0.00	0.07
C7	inputs	388.88	8000.00	0.00	164.63
C8	inputs	9.39	281.00	0.00	4.05
C9	inputs	315779.56	20000000.00	0.00	156296.54
C10	outputs	3.96	5.00	0.00	1.96
C11	outputs	3.96	16.00	0.00	1.76
C12	outputs	13351.09	257815.00	0.00	6125.16
C13	outputs	10270.32	99403.00	0.00	4745.70
C14	inputs	2.11	30.00	0.00	0.95
C15	inputs	7.96	318.00	0.00	3.46
C16	inputs	3.65	18.00	0.00	1.78
C17	inputs	39.54	1008.00	0.00	18.71
C18	inputs	2.54	30.00	0.00	1.17
C19	0.00	4.52	5.00	4.20	1.97
C20	outputs	4.56	5.00	4.20	2.18
C21	outputs	4.60	4.90	4.20	2.18
C22	outputs	34.36	900.00	0.00	15.99
C23	outputs	0.36	5.63	0.00	0.16
C24	outputs	4.96	5.00	4.60	2.09
C25	outputs	0.04	2.00	0.00	0.02
C26	outputs	4.53	4.90	4.20	1.88
C27	outputs	4.55	5.00	4.20	1.98
C28	outputs	4.53	4.90	4.20	1.88
C29	outputs	4.55	5.00	4.20	1.87
C30	outputs	4.59	4.90	4.20	1.89
C31	outputs	4.53	4.90	4.20	1.98
C32	outputs	4.55	4.90	4.20	1.87
C33	outputs	4.57	4.90	4.20	1.97

### 4.2 DEA analysis

MaxDEA Basic 6.13 is one of the most user-friendly softwares in SBM-DEA study. Based on the above principles and software operation, the performance of CECSCs in Nanjing was measured and the results are shown in Table 3.

**Table 3:** Performance efficiency of CECSCs in Nanjing.

DMU	Score	DMU	Score	DMU	Score	DMU	Score	DMU	Score
001	0.454	041	1.000	081	1.000	121	1.000	161	1.000
002	0.213	042	1.000	082	1.000	122	0.258	162	0.404
003	0.505	043	0.426	083	0.824	123	0.768	163	0.830
004	0.139	044	0.768	084	0.909	124	0.603	164	1.000
005	1.000	045	0.762	085	1.000	125	0.615	165	0.195
006	1.000	046	1.000	086	0.311	126	0.921	166	0.672
007	1.000	047	0.889	087	1.000	127	1.000	167	0.200
008	0.618	048	1.000	088	0.962	128	1.000	168	1.000
009	0.235	049	0.823	089	1.000	129	1.000	169	1.000
010	1.000	050	0.982	090	0.114	130	1.000	170	0.498
011	1.000	051	0.945	091	0.493	131	0.446	171	0.542
012	1.000	052	0.784	092	0.500	132	1.000	172	1.000
013	0.846	053	0.000	093	0.971	133	1.000	173	1.000
014	0.642	054	0.167	094	0.234	134	1.000	174	0.418
015	0.336	055	0.000	095	0.541	135	0.352	175	0.620
016	1.000	056	1.000	096	1.000	136	1.000	176	0.765
017	1.000	057	0.692	097	1.000	137	1.000	177	1.000
018	1.000	058	1.000	098	0.451	138	1.000	178	0.674
019	0.501	059	1.000	099	0.655	139	0.278	179	0.625
020	0.718	060	0.625	100	0.513	140	0.980	180	0.520
021	0.373	061	0.629	101	0.513	141	0.566	181	0.856
022	1.000	062	0.323	102	1.000	142	1.000	182	1.000
023	0.243	063	0.957	103	0.162	143	0.478	183	0.624
024	1.000	064	1.000	104	1.000	144	0.419	184	1.000
025	0.150	065	1.000	105	0.755	145	0.239	185	0.852
026	0.223	066	1.000	106	0.630	146	0.074	186	0.687
027	1.000	067	0.640	107	0.528	147	0.125		
028	1.000	068	0.417	108	1.000	148	0.871		
029	0.692	069	1.000	109	0.888	149	0.690		
030	1.000	070	0.826	110	1.000	150	0.086		
031	1.000	071	1.000	111	0.440	151	0.100		
032	1.000	072	1.000	112	0.575	152	0.385		
033	0.864	073	0.413	113	0.423	153	0.376		
034	0.791	074	1.000	114	1.000	154	0.441		
035	0.260	075	0.926	115	1.000	155	1.000		
036	1.000	076	1.000	116	1.000	156	0.585		
037	0.354	077	0.693	117	1.000	157	0.100		
038	1.000	078	1.000	118	0.526	158	0.648		
039	1.000	079	0.714	119	0.959	159	0.627		
040	1.000	080	0.247	120	1.000	160	1.000		

The efficiency value is divided into effective and invalid value, and the invalid efficiency value is divided into three levels as shown in Table 4. It can be seen from Table 3 that among 186 CECSCs, 75 CECSCs are effective and 111 remaining CECSCs are invalid. In addition, the projection by the SBM-DEA model represents a practical “improvement”. The inputs and outputs of the projection of each inefficient DMU on the frontier are shown in appendix. It is easy to see that: inefficient DMUs should use lower inputs and higher outputs than the original values in order to reach the frontier; other DMUs should reduce (expand) at least one of its outputs (inputs) to reach the frontier.

**Table 4:** Performance efficiency statistics of CECSCs in Nanjing.

Scores range	Number of DMU	Efficiency value evaluation	
Score = 1	75	Efficient	
0.8≤Score<1	21	Invalid	The first level
0.4≤Score<0.8	58		The second level
0≤Score<0.4	32		The third level

As a typical example in the effective level, DMU059 is one of the earliest CECSC developed in Nanjing and even in the country. With well-equipped hardware facilities, high degree of management standardization and above-average service specialization, DMU059 is a leader in the community and home-based elderly care industry of Nanjing. DMU126(efficiency score = 0.921) was selected from the first level of SBM-DEA invalidation for analysis. This organization mainly provides spiritual consolation services for the elderly, but its emphasis on spirit rather than substance leads to its weak economic benefits, which partly affects its performance. However, the organization has a wide range of elderly care recipients, a long service time, a good service effect, and a high degree of management standardization. As a result, it performs well in all but economic indicators. DMU043(efficiency score = 0.426) was selected from the second level of SBM-DEA invalid for analysis. Due to its short opening time, low popularity, unstable source of users, and its huge investment cost in the early stage, the institution is greatly affected by the economic situation. DMU152(efficiency score = 0.385) was selected from the third level of SBM-DEA invalid for analysis. There are more well-known service centers around the organization, which affect its economic benefits. In addition, due to its poor reputation, customer satisfaction is relatively low. Moreover, the operating cost is ineffectively controlled, which leads to its unsatisfactory performance.

Meanwhile, the evaluation result of SBM-DEA efficiency value is compared with the existing grades of CECSCs in Nanjing, as shown in the Table 5. Compared with the rating results of current CECSCs, the evaluation results of SBM-DEA efficiency value are broadly similar in general trend but still differ in the evaluation of individual cases. This study indicates that the main reason is that the current evaluation of CECSCs is merely focusing on the hardware, ignoring the requirements for management efficiency. This reflects some problems in the reality, that is, many CECSCs use sufficient funds to buy hardware, but neglect the operation management and service quality. Although these social organizations can be rewarded a high rating in the evaluation, they cannot satisfy end-users, which defeats the purpose of the evaluation.

**Table 5:** Comparison of SBM-DEA performance efficiency and the existing grades of CECSCs in Nanjing.

	Score = 1	0.8≤Score<1	0.4≤Score<0.8	0≤Score<0.4	Total
AAA	43	13	40	13	109
AAAA	24	4	11	12	51
AAAAA	8	4	6	7	25
Total	75	21	57	32	186

### 4.3 Sensitivity analysis

After identifying the gap, it is of necessity to determine the direction of performance optimization. In this study, SA method is adopted to study the effect of improving various indicators on the overall efficiency value of CECSCs. Three DMUs are randomly selected from the three levels where the efficiency value is invalid. Table 6 shows sensitivity analysis of output indicators of DMU126(first level), DMU043(second level) and DMU152(third level).

**Table 6:** Sensitivity Analysis of output indicators.

Indicators	DMU126							DMU043							DMU152						
	-30%	-20%	-10%	0	10%	20%	30%	-30%	-20%	-10%	0	10%	20%	30%	-30%	-20%	-10%	0	10%	20%	30%
C4	0.921	0.921	0.921	0.921	0.921	0.921	0.921	0.426	0.426	0.426	0.426	0.426	0.426	0.426	0.385	0.385	0.385	0.385	0.385	0.385	0.385
C10	**0.792**	**0.801**	**0.835**	**0.921**	**-**	**-**	**-**	**0.426**	**0.426**	**0.426**	**0.426**	**-**	**-**	**-**	**0.290**	**0.320**	**0.353**	**0.385**	**-**	**-**	**-**
C11	0.921	0.921	0.921	0.921	0.921	0.921	0.921	0.426	0.426	0.426	0.426	0.426	0.426	0.426	0.385	0.385	0.385	0.385	0.385	0.385	0.385
C12	0.921	0.921	0.921	0.921	0.921	0.921	0.921	0.426	0.426	0.426	0.426	0.426	0.426	0.426	0.385	0.385	0.385	0.385	0.385	0.385	0.385
C13	0.921	0.921	0.921	0.921	0.921	0.921	0.921	0.426	0.426	0.426	0.426	0.426	0.426	0.426	0.385	0.385	0.385	0.385	0.385	0.385	0.385
C19	0.921	0.921	0.921	0.921	0.921	-	-	0.426	0.426	0.426	0.426	0.426	0.426	0.426	0.385	0.385	0.385	0.385	0.385	0.385	0.385
C20	0.921	0.921	0.921	0.921	0.921	-	-	0.426	0.426	0.426	0.426	0.426	0.426	0.426	0.385	0.385	0.385	0.385	0.385	0.385	0.385
C21	0.921	0.921	0.921	0.921	0.921	0.921	0.921	0.426	0.426	0.426	0.426	0.426	-	-	0.385	0.385	0.385	0.385	0.385	-	-
C22	**0.921**	**0.921**	**0.921**	**0.921**	**0.923**	**0.962**	**0.979**	**0.305**	**0.341**	**0.384**	**0.426**	**0.469**	**0.512**	**0.568**	**0.382**	**0.383**	**0.384**	**0.385**	**0.386**	**0.388**	**0.390**
C23	**0.921**	**0.921**	**0.921**	**0.921**	**0.924**	**0.929**	**0.935**	**-**	**-**	**-**	**0.426**	**-**	**-**	**-**	**0.375**	**0.378**	**0.381**	**0.385**	**0.390**	**0.394**	**0.399**
C24	0.921	0.921	0.921	0.921	-	-	-	0.426	0.426	0.426	0.426	-	-	-	0.385	0.385	0.385	0.385	-	-	-
C26	0.921	0.921	0.921	0.921	0.921	0.921	0.921	0.426	0.426	0.426	0.426	0.426	0.426	0.426	0.385	0.385	0.385	0.385	0.385	0.385	0.385
C27	0.921	0.921	0.921	0.921	0.921	0.921	0.921	0.426	0.426	0.426	0.426	0.426	0.426	0.426	0.385	0.385	0.385	0.385	0.385	0.385	0.385
C28	0.921	0.921	0.921	0.921	0.921	0.921	0.921	0.426	0.426	0.426	0.426	0.426	0.426	0.426	0.385	0.385	0.385	0.385	0.385	-	-
C29	0.921	0.921	0.921	0.921	0.921	0.921	0.921	0.426	0.426	0.426	0.426	0.426	-	-	0.385	0.385	0.385	0.385	0.385	-	-
C30	0.921	0.921	0.921	0.921	0.921	-	-	0.426	0.426	0.426	0.426	0.426	0.426	0.426	0.385	0.385	0.385	0.385	0.385	0.385	0.385
C31	0.921	0.921	0.921	0.921	0.921	0.921	0.921	0.426	0.426	0.426	0.426	0.426	0.426	0.426	0.385	0.385	0.385	0.385	0.385	0.385	0.385
C32	0.921	0.921	0.921	0.921	0.921	0.921	0.921	0.426	0.426	0.426	0.426	0.426	0.426	0.426	0.385	0.385	0.385	0.385	0.385	-	-
C33	0.921	0.921	0.921	0.921	0.921	0.921	0.921	0.426	0.426	0.426	0.426	0.426	0.426	0.426	0.385	0.385	0.385	0.385	0.385	-	-

**Fig 2:**
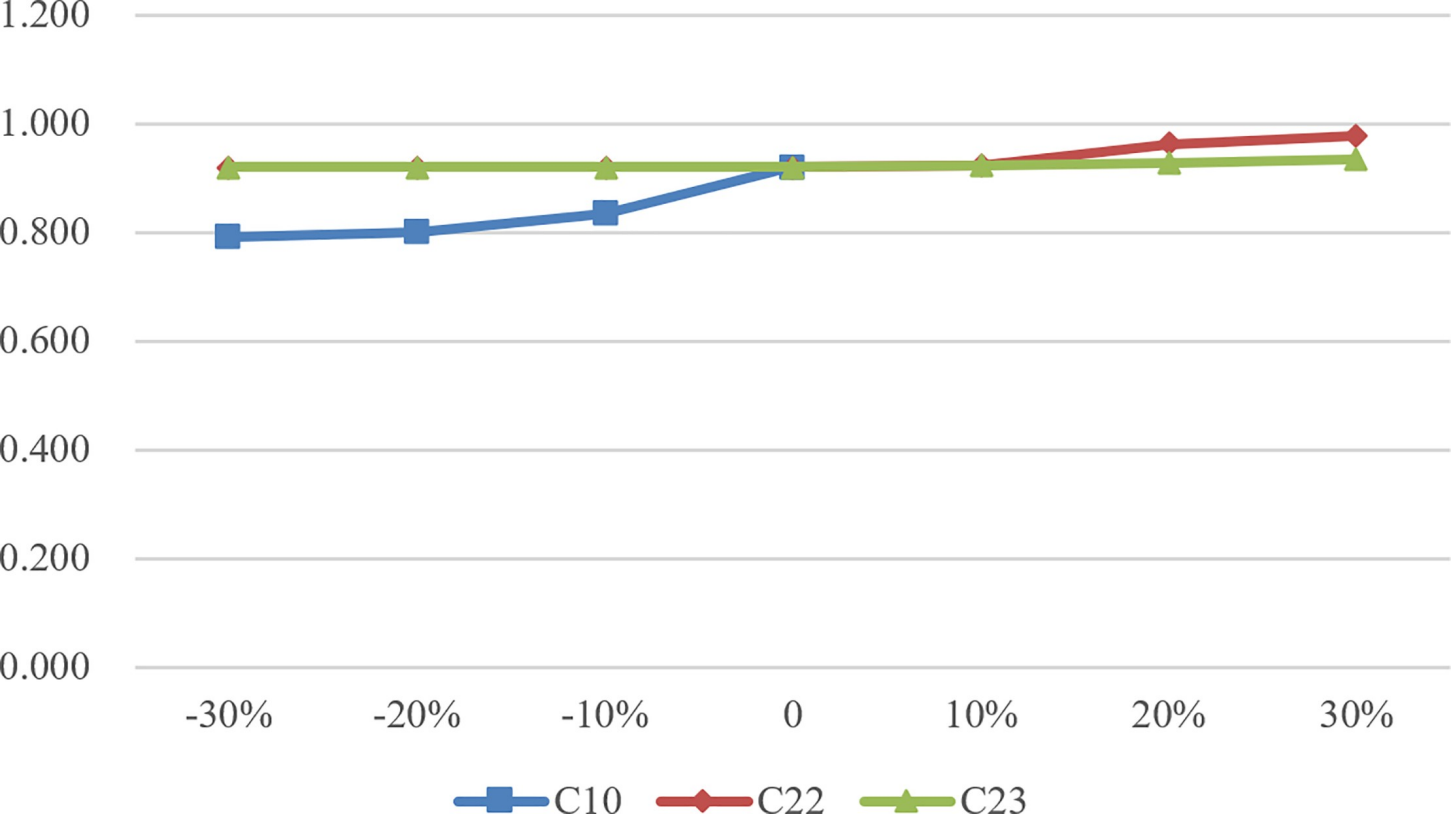
Efficiency value change of DMU126.

**Fig 3:**
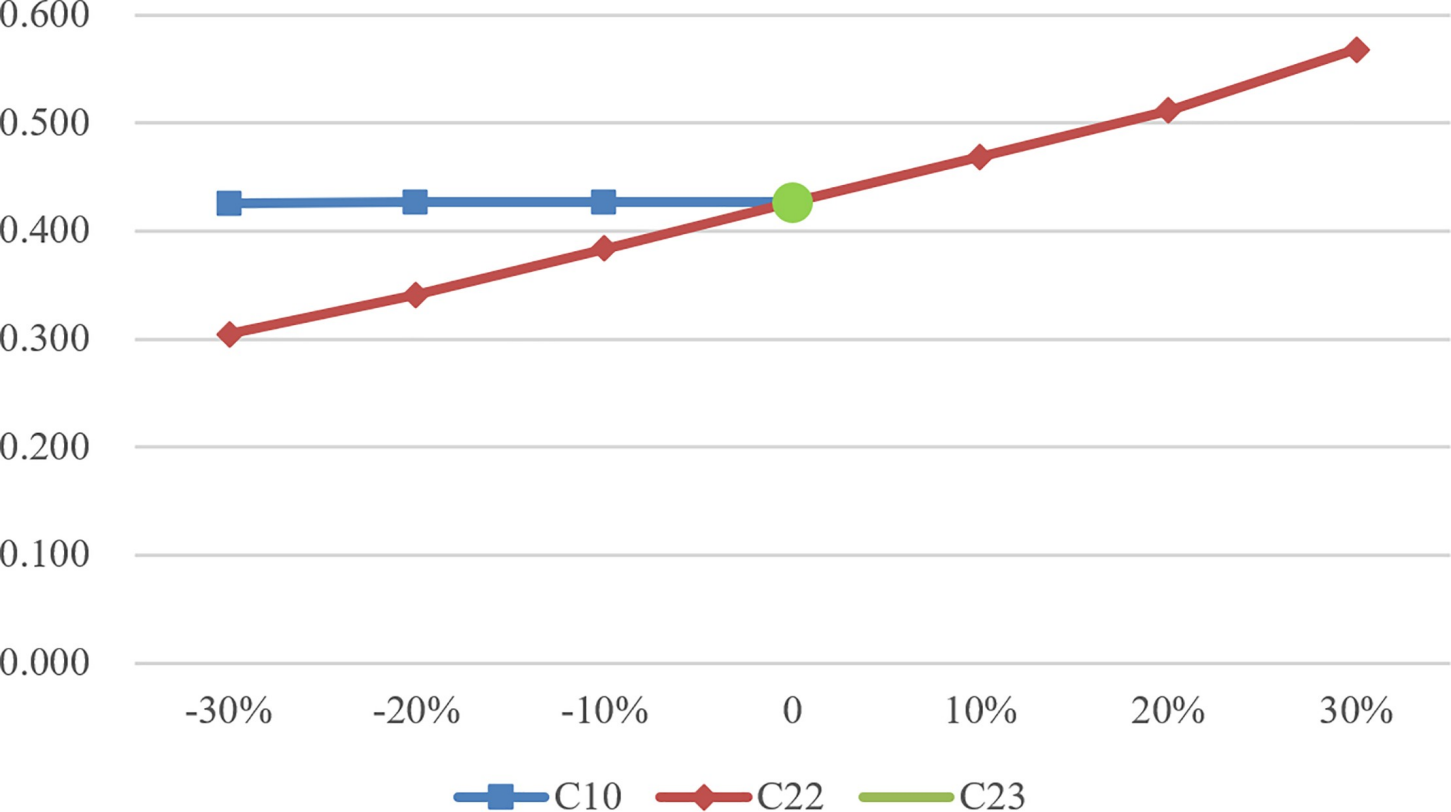
Efficiency value change of DMU043.

**Fig 4:**
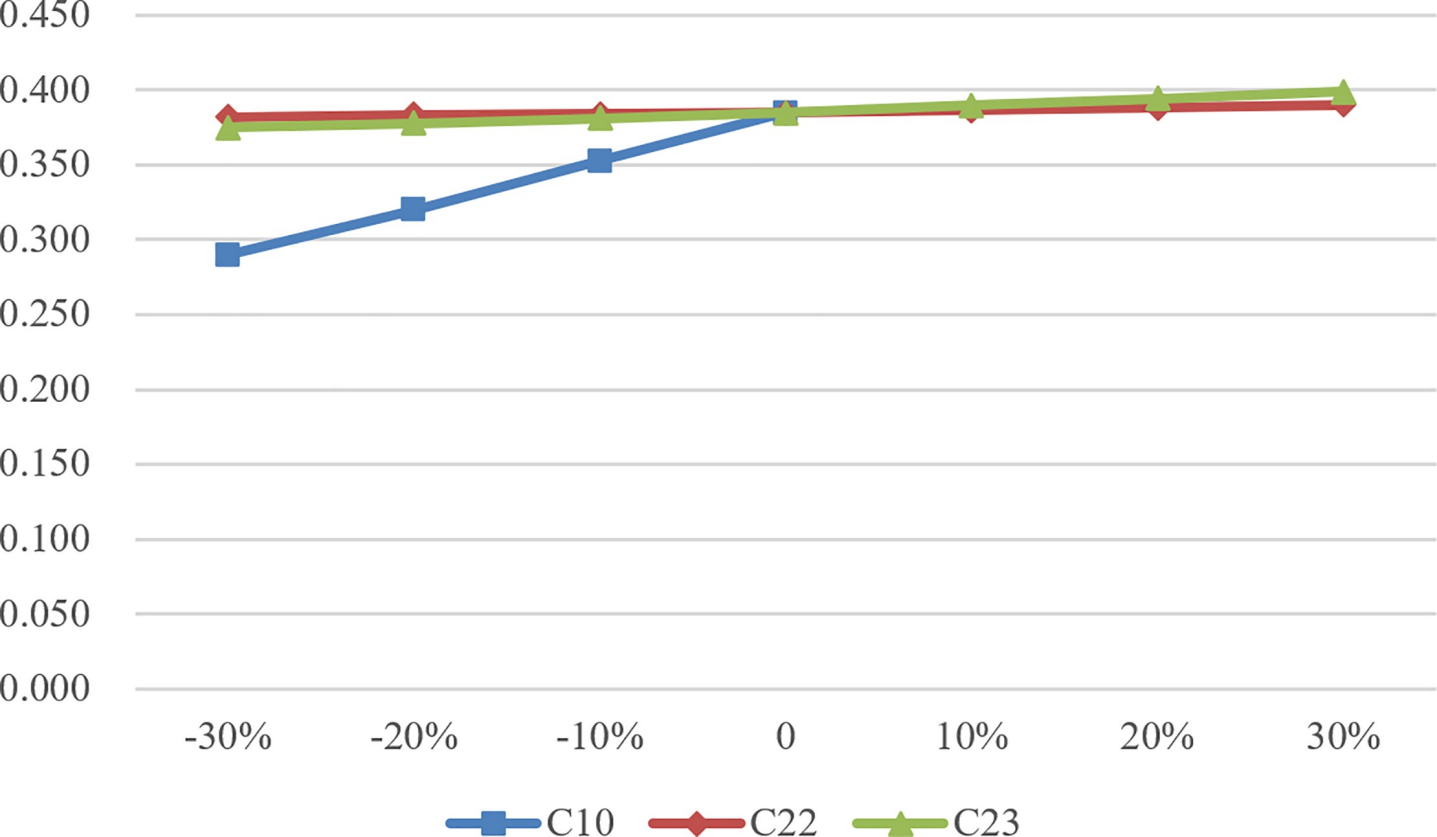
Efficiency value change of DMU152.

Although it is difficult to change the overall efficiency value through a certain DMU’s indicator due to the large number of input and output indicators, it can be seen from Figs 2 to 4 that the change of C10, C22, and C23 has a significant impact on the change of efficiency value. According to SA, as the basic demand of the largest number of the elderly, C10 plays a crucial role in the performance evaluation of CECSCs. Meanwhile, C22 and C23 also affect the efficiency value of CECSCs to a large extent. As for the other indicators, such as the indicators in team development dimension and organization construction dimension, they belong to the internal development category of CECSCs, and the optimization of this category is difficult to improve efficiency value directly and significantly, which can only indirectly affect the efficiency value by attracting end-users and increasing operational income.

### 4.4 Data comparative analysis

To observe the inputs and outputs of invalid DMUs and find out the reasons for the performance gap, SBM-DEA model is employed. This study randomly selects an invalid decision unit from each of the three invalid levels, which is the DMU126(efficiency score = 0.921) in the first level, DMU043 (efficiency score = 0.426) in the second level and DMU152(efficiency score = 0.385) in the third level. In order to provide a comprehensive analysis, the criteria for the selection of invalid decision unit takes into account CECSC from urban and rural districts with different ratings.

#### 1) DMU126

**Fig 5:**
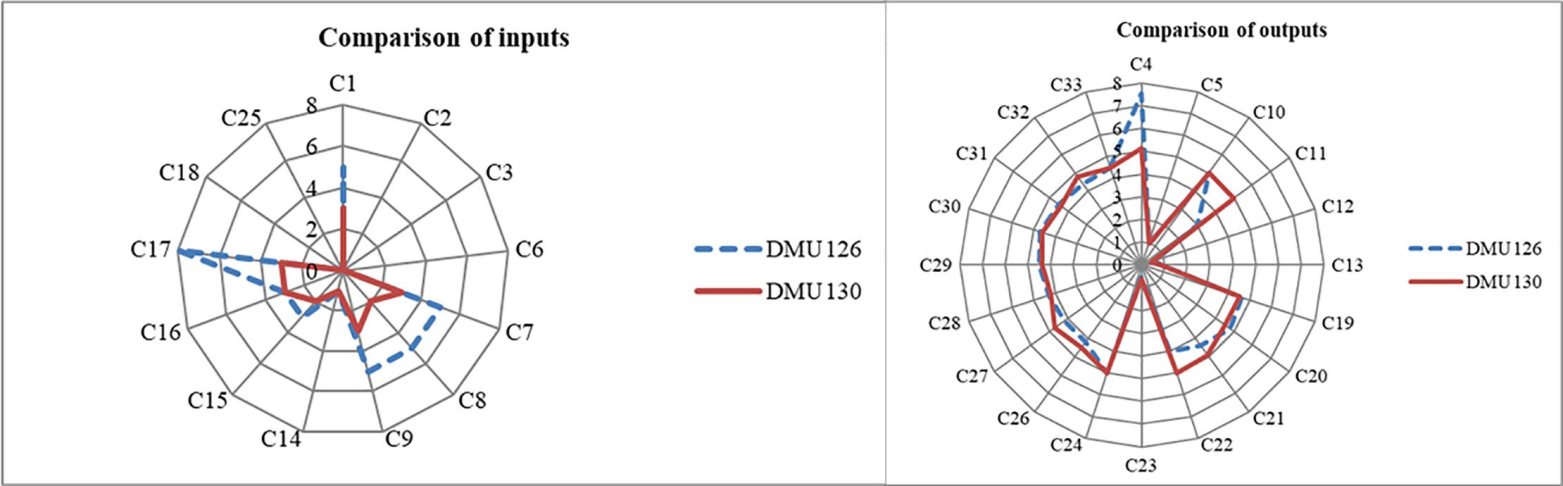
Comparison of DMU126 and DMU130 inputs and outputs.

For the comparative analysis of CECSCs at the first level, several DMUs which are located in the same area as DMU126 in Nanjing city and have relatively consistent input indicators of capital source, hardware facilities and team composition were selected. On this basis, the DMU with the highest efficiency score is selected for comparison. Hence, DMU130 (efficiency score = 1.000) with the highest performance efficiency is chosen for comparison. The comparison of inputs and outputs of DMU126 and DMU130 is shown in Fig 5.

Although almost all inputs of DMU126 are higher than that of DMU130, DMU126 has no absolute advantage over DMU130 in outputs except in terms of C4. Moreover, it is even deficient in C11, C21, C22, and C23.This clearly shows that DMU130 is able to convert its inputs into outputs more efficiently than DMU126 by enhancing internal management measures. Hence, it is necessary to improve the ability of operation management and provide accurate service to improve organizational performance.

#### 2) DMU043

**Fig 6:**
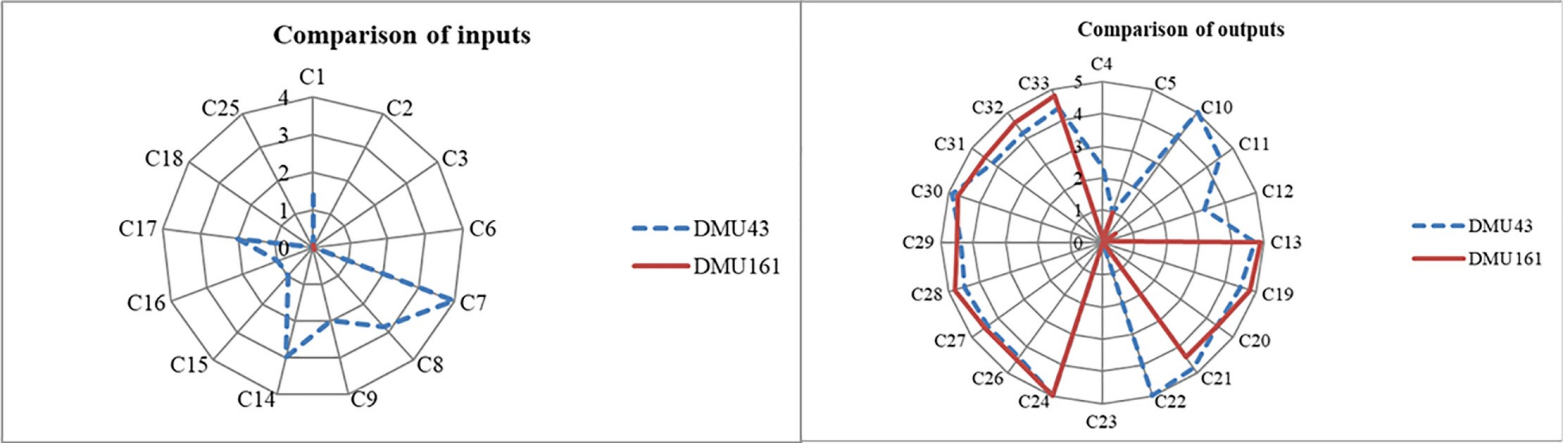
Comparison of DMU043 and DMU161 inputs and outputs.

Taking the approach of selecting the DMU for comparative analysis from the first level, DMU161 (efficiency score = 1.000) with the highest performance efficiency is chosen for comparison. The comparison of inputs and outputs of DMU043 and DMU161 is shown in Fig 6.

As is shown in Fig 6, DMU043, which is the representative of high-standard CECSC in Nanjing, has significant advantages in all inputs due to receiving significant capital support. In contrast, DMU161, as the representative of CECSCs away from the city center, only has one service type, but it has reached higher performance than DMU043 which has nearly ten services. Therefore, an important breakthrough in improving organization performance is to grasp the demand on customer group with different consumption ability and preference. CECSCs should pay more attention to improve the quality of existing services and continuously enhance the satisfaction of the elderly while investing in abundant funds.

#### 3) DMU152

**Fig 7:**
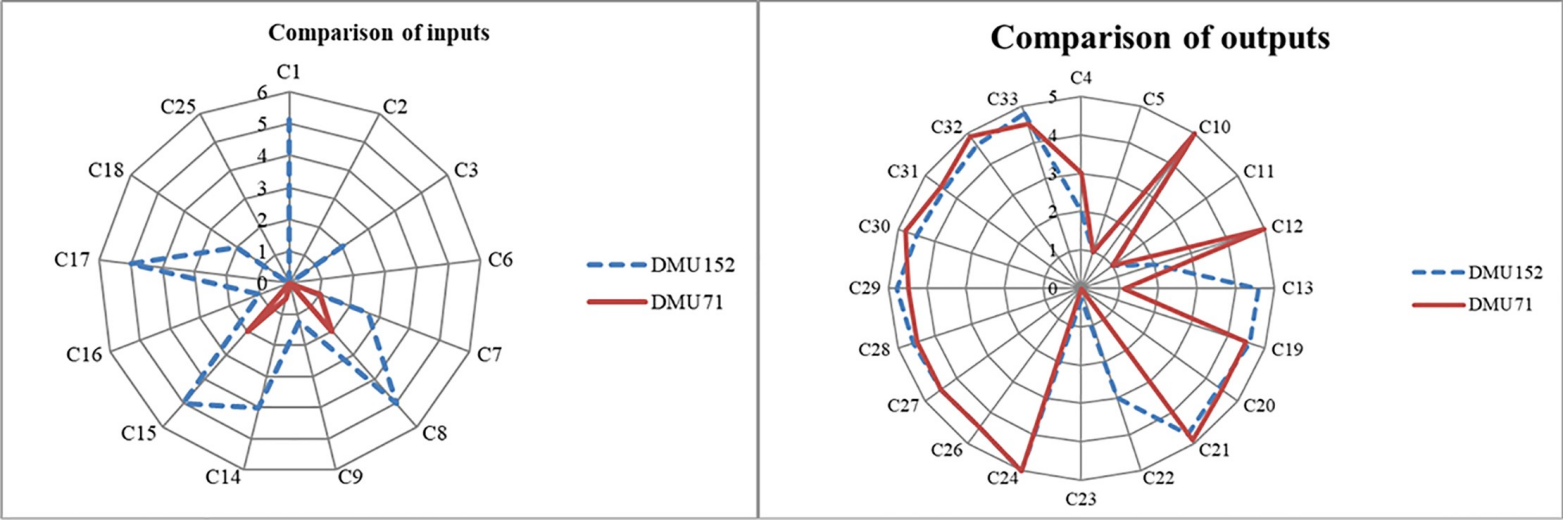
Comparison of DMU152 and DMU071 inputs and outputs.

Taking the approach of selecting the DMU for comparative analysis from the first level, DMU071 (efficiency score = 1.000) with the highest performance efficiency is chosen for comparison. The comparison of inputs and outputs of DMU152 and DMU071 is shown in Fig 7.

As is shown in Fig 7, it is interesting to note that DMU071, which has relatively low inputs, has equal outputs to DMU152. The finding is in accordance with numerous advantages from developing service chain of the CECSCs. It has been demonstrated that it is the service chain that promotes efficient utilization of headquarter valuable resources for CECSCs, handles the requirements of all the elderly appropriately, maximizes long term profitability and so on. In addition, DMU071 focuses on the basic service, namely food, bath, cleaning, emergency, and treatment, while DMU152 chooses multi-channel development, placing equal emphasis on basic service and other value-added service functions. The efficiency scores of CECSCs can also be promoted by improving management measures systematically according to the region’s different demand while developing service chain.

#### (2) Design of Performance Improvement Path

The core of performance improvement for CECSCs lies in cost reduction and efficiency enhancement. Under the condition that the investment remains unchanged, efforts should be made to focus on core business, improve management ability, and comprehensively enhance service quality, so as to achieve the goal of performance improvement. As mentioned above, the key output indicators of CECSCs are C10, C22, and C23. Taking DMU126, DMU043, and DMU152 as examples, since the categories of C10 have reached the upper limit and cannot be further improved, both C22 and C23 are increased simultaneously (the indicator C23 of DMU043 is 0, so only the effect of improving C22 on the efficiency value is considered). Therefore, the efficiency value will be greatly improved, as shown in Table 7.

From Table 7, it can be seen that C22 and C23 have a profound impact on the performance of CECSCs, and the greater the improvement, the greater the impact on the performance. Taking DMU152 as an example, as a rural CECSCs, the center fails to give full play to its geographical advantages. The coverage rate of the elderly in this community is only 27%, which is lower than the average 36% of the 186 CECSCs in Nanjing. In addition, the number of elderly people whose services are procured by the government is only 3, with huge space for improvement.

**Table 7:** The effect of improving C22 and C23 on the efficiency value of DMU126, 152 and 043.

DMU126		DMU152		DMU043	
Improve rate of C22 and C23	Efficiency value	Improve rate of C22 and C23	Efficiency value	Improve rate of C22	Efficiency value
0	0.92068	0	0.385005	0	0.426464
10%	0.925596	10%	0.39108	10%	0.469111
20%	0.970999	20%	0.397155	20%	0.511757
30%	1	30%	0.403231	30%	0.554404
40%	1	40%	0.410632	40%	0.59705
50%	1	50%	0.418532	50%	0.639696
……	-	……	-	……	-
100%	1	100%	0.537364	100%	0.852929

Therefore, the next step should focus on improving the service quality and attracting more end-users for this center. Rural areas are more willing to give priority to home-based elderly care, which is different from that in cities. Therefore, according to this characteristic, the center is suggested to encourage more rural elders to try and gradually adapt to the home-based elderly care by clearing requirements, improving service quality, and strengthening propagatition without substantial cost increase, which can eventually reach the purpose of cost reduction and efficiency increase.

Meanwhile, this paper lays emphasis on the performance optimization of the input indicators while strengthening the output. Since the capital investment, the occupancy nature and the total amount of fixed assets are one-time inputs, it is difficult to change. Therefore, this paper mainly studies the impact of indicators C7, C8 and C14-C18 on the performance. The study still takes DMU152 as an example.

From Table 8, it can be seen that for DMU152, the idea of improving the performance level by reducing investment is feasible. However, according to the Value Engineering Theory, the premise of increasing value by reducing input is that the output cannot fall significantly, otherwise it cannot improve performance [105].

**Table 8:** The effect of changing C7, C8 and C14-C18 on the efficiency value of DMU152.

Change rate of C7	Efficiency value	Change rate of C8	Efficiency value	Change rate of C14-C18	Efficiency value
0	0.385005	0	0.385005	0	0.385005
-10%	0.402539	-10%	0.405032	-10%	0.393517
-20%	0.442703	-20%	0.427919	-20%	0.404574
-30%	0.494397	-30%	0.453549	-30%	0.431091
-40%	0.55976	-40%	0.482444	-40%	0.46431
-50%	0.645038	-50%	0.515271	-50%	0.540786

For DMU152, the annual service number is only 15,000, which is less than the average number of Nanjing (24,000). However, it has 250 m^2^ space, 10 beds and ¥1.22 million of fixed assets, which are all higher than the average level of the city. By improving the utilization rate of space and beds, reducing the space and the number of beds, and changing the space and beds to other functions, the performance can be effectively improved. Simultaneously, as a rural CECSC, there are 26 staffs, which is obviously redundant. While improving the working efficiency of staff and ensuring the quality of service, the number of staffs and expenses can be reduced to achieve the purpose of cost reduction and efficiency increase.

## 5. Implications and suggestions

The participation of social organizations in community and home-based elderly care service is related to a series of strategies and actions aiming to achieve elderly satisfaction and requirements with the main principles of welfare society. All the different aspects (e.g., finance, facilities, and administration) can significantly influence the level of performance of CECSCs. After a careful examination, some implications and suggestions can be provided, based on the shortcomings in the CECSC efficiencies in Nanjing. The performance improvement of the elderly care should involve the government, the industry association and CECSC. Each participant has the responsibility to make efforts to the development of the industry.

### 5.1 Government authorities

As the coordinator of CECSCs, the government needs to provide macro guidance for the comprehensive, coordinated and sustainable development of the industry. Firstly, government should guarantee and implement policies in land, finance, health and other aspects. For example, the government can increase investment (C1) in CECSCs to reduce their initial investment (C6) including construction costs and land costs. Government can provide subsidies when CECSCs purchase hardware facilities (C8, C9). Secondly, government should use the policy guidance to enhance the competitiveness of social organizations and stimulate the innovation ability of social organizations. Through the adjustment of service organization system (C30), professional division of labor (C14, C15, C16, C17, C18) and other aspects, it can help improve operational efficiency and reduce operating costs. Thirdly, government should carry out classified management according to the actual situation of CECSCs. As can be seen from the results of SBM-DEA, some CECSCs spend partial funds to improve the hardware to obtain high rating, but neglect the improvement of operation management. For the small-scale CECSCs, the rigid requirements on their hardware should be reduced; the focus of performance evaluation should be placed on the quality of service (C12, C13) and operational management (C30, C31). End-users’ satisfaction (C24) should be placed in a prominent position, so as to avoid the undesirable phenomenon that performance evaluation becomes investment comparison.

### 5.2 Industry associations

As a department authorized by the government, the industry association not only undertakes part of administrative functions but also shoulder the functions of training, assessment and supervision. Firstly, the industry association should strengthen supervision and evaluation. For example, the industry association should pay attention to forming an all-round and multi-level supervision system and building a multi-channel feedback mechanism (C32, C33). In addition, it attaches importance to the output indicators of performance evaluation to guide the rational investment of social organizations(C4). Secondly, the industry association should improve the quality of service personnel. The industry association is advised to increase the intensity and frequency of personnel training(C20, C21), and give priority to ensuring that training funds are fully implemented on the premise of government support, social participation and market promotion(C5). Thirdly, the industry association should diversify participation and improve management. The industry association is suggested to guide and encourage social organizations to actively participate in the community and home-based elderly care service industry(C2, C3), and increase the investment in living conditions, skills training and salary incentives for elderly care workers (C7, C21, C29), so as to maximize resource conservation and efficiency.

### 5.3 CECSCs

As the results demonstrate, in some CECSCs, there exist low performance efficiency despite high capital investment. In order to improve operation performance, CECSCs should strive to improve the ability of internal administration (B11, B12, B13). Meanwhile, as the most significant indicators, the level of community coverage (C22, C23) and the level of Customer satisfaction (C24), should also be given due attention to. It is mandatory for CECSCs to provide differentiated services that are based on accurate analysis of consumption ability and preference in community. For example, the CECSCs that are spread in underdeveloped area should enhance the efficiency in the organization by providing basic service with high quality. In addition, the development of CECSCs needs to balance service types and service quality (C10, C11), meaning the pursuit of highly professional service is crucial. It is suggested that CECSCs avoid to blindly expand the scale and increase the types of services, but implement professional service to improve the customer satisfaction. At present, some CECSCs in Nanjing have realized chain operation is conducive to the development of organizations by achieving scale effect. Through SBM-DEA analysis, the service chain operation should facilitate internal resource allocation as well as reduce costs, consequently achieving performance improvement.

## 6. Conclusion

This study proposes a systematic framework for performance evaluation of CECSCs with 33 indicators developed from literature review and field interviews. These indicators help to evaluate performance of the CECSCs from six dimensions: financial management, hardware facilities, team development, service management, elderly care recipients and organization construction. On the basis of the different CECSCs in Nanjing in 2018, the proposed SBM-DEA method is able to effectively measure the performance efficiency and discern the inefficiencies in its internal management. As an important consideration, the impact of region and quality grading is also considered in the evaluation. This study proves that the performance of 75 CECSCs in Nanjing is effective, while that of the remaining 111 is not. This result is similar in general trend but still differ in individual cases compared with the existing rating results of CECSCs. To test the stability of SBM-DEA results, sensitivity analysis has been conducted in the models by changing outputs, which determines the direction of performance optimization. Hence, the performance evaluation system proposed in this paper can be used in practice. Some relevant suggestions for government authorities, industry associations and CECSCs have also been offered.

However, this study is also subjective to some limitations. The first restriction is that social impact, the most pivotal aspect of the performance evaluation of social organizations, still needs to be improved. Another limitation is that a large quantity of valuable data cannot be effectively applied in the research due to the different statistical diameters, which needs further exploration in the future.

## Supporting information

S1 Table Grading criteria of indicators. (DOCX)

S2 Table Raw value of indicators. (DOCX)

S1 Appendix (DOCX)
