# Supplementary material for: A SBM-DEA based performance evaluation and optimization for social organizations participating in community and home-based elderly care services
Source: PLoS One. 2021 Mar 17;16(3):e0248474. doi: 10.1371/journal.pone.0248474 (PMC7968683; doi:10.1371/journal.pone.0248474)
Supplement: S1 Table — (DOCX) [file pone.0248474.s001.docx]

**S1 Table. Grading criteria of indicators.**

| **Indicators** | **Explanation** | **Data source** |
| --- | --- | --- |
| C1  Government funding | The sum of government investment per year | The Elderly Care Association in Nanjing |
| C2  Donated funding | The sum of donated investment per year | The Elderly Care Association in Nanjing |
| C3  Other funding | The sum of other investment per year | The Elderly Care Association in Nanjing |
| C4  Operating income | The sum of operating income per year | Nanjing elderly care information platform and price list of service provided by the Elderly Care Association.  Service price (RMB per time): food=7, bath=50, cleaning=20, emergency=50, treatment=20. |
| C5  Overall financial evaluation | The sum of operating income per year/ The sum of investment per year | C5’=C4/C1+C2+C3;  C5’≥10%，C5=1;  5%≤C5’＜10%，C5=0.5;  C5’＜5%，C5=0 |
| C6  Land Use | The Land ownership of CECSCs | The Elderly Care Association in Nanjing  Given by the community for free=0;  Rental=1;  Self-built or self-owned=2. |
| C7  Size of site | The area of CECSCs | Contract or real estate certificate provided by the CECSC to the Elderly Care Association when submitting the rating declaration. |
| C8  Number of beds | Number of beds in CECSCs | The Elderly Care Association in Nanjing |
| C9  Total amount of fixed assets | Total amount of fixed assets of CECSCs | The Elderly Care Association in Nanjing |
| C10  Types of basic services | Types of basic service, including food, bath, cleaning, emergency, and treatment | Extracted from the statistics of Nanjing elderly care information platform |
| C11  Types of other services | Types of other services | Extracted from the statistics of Nanjing elderly care information platform |
| C12  Quality of basic services | Quality of basic service, including food, bath, cleaning, emergency, and treatment | Extracted from the statistics of Nanjing elderly care information platform  Number of credit card swipes during service |
| C13  Quality of other services | Quality of other services | Extracted from the statistics of Nanjing elderly care information platform  Number of credit card swipes during service |
| C14  Number of administrative staff | Number of managers in CECSCs | The Elderly Care Association in Nanjing |
| C15  Number of staff | Number of staff in CECSCs | The Elderly Care Association in Nanjing |
| C16  Number of social workers | Number of social workers in CECSCs | The Elderly Care Association in Nanjing |
| C17  Number of volunteers | Number of volunteers in CECSCs | The Elderly Care Association in Nanjing |
| C18  Number of other service personnel | Number of other person in CECSCs, such as nurses | The Elderly Care Association in Nanjing |
| C19  Qualification management evaluation | The qualification level of CECSCs | The Elderly Care Association  A scoring system is adopted, with a 5-point system as the full score and a basic score of 4 points.  Administrative staff can get 0.2 points for each intermediate or higher qualification certificate.  Staff can get 0.1 for each intermediate or higher qualification certificate.  No more than 5 points. |
| C20  Training management level | The regular training quantity of CECSCs | Nanjing pension service management (nursing) talents.  A scoring system is adopted, with a 5-point system as the perfect score and a basic score of 4 points.  Administrative staff can get 0.2 points for getting trained once or more per person per year.  Staff can get 0.1 points for getting trained once or more per person per year.  No more than 5 points. |
| C21  Employee satisfaction level | Job satisfaction of employees in CECSCs | Nanjing pension service management (nursing) talents |
| C22  Number of old people served | Number of old people that government's purchased service | The Elderly Care Association in Nanjing |
| C23  Coverage of social services for the elderly | The ratio of the number of elderly people serving to the number of elderly people in the community | Elderly Care Association.  Number of valid card users in each CECSC /number of effective demand end-users in this community. |
| C24  Elderly satisfaction | Satisfaction with the services provided by the elderly | A questionnaire on the satisfaction of the elderly care service in Nanjing conducted by the Elderly Care Association. |
| C25  Number of complaints | Number of complaints | The Elderly Care Association in Nanjing |
| C26  Degree of financial regulations perfection | Evaluating whether there are detailed, clear, operable financial management procedures and regulations | A scoring system is adopted, with a five-point system as the full score and a basic score of four points, to evaluate the five aspects of payment management, financial planning, financial vouchers, financial handover, and financial supervision.  0.2 points for the detailed, clear and operable regulations in each area.  0.1 points if there are only relevant regulations, but not specific and complete.  No more than 5 points.  Marking according to the financial system provided by each CECSC when applying for rating. |
| C27  Reasonable degree of organizational structure | Evaluating whether there are detailed, clear, operable organization management procedures and regulations | Adopt a scoring system, with a five-point system as the full score and a basic score of four points, and evaluate the seven aspects of staff job requirements, center responsibilities, organizational structure, department authority, responsibility settings, professional staff responsibilities, financial staff responsibilities and service staff responsibilities.  0.2 points for the detailed, clear and operable regulations in each area.  0.1 points if there are only relevant regulations, but not specific and complete.  No more than 5 points.  Marking according to the position system provided by each CECSC when applying for rating. |
| C28  Degree of security system perfection | Evaluating whether there are detailed, clear, operable safety management procedures and regulations | A scoring system is adopted, with a five-point system as the perfect score and a basic score of four points.  Five aspects of full-time staff position setting, one-key call system, fire protection measures, anti-theft protection measures, and duty system are evaluated.  0.2 points for the detailed, clear and operable regulations in each area.  0.1 points if there are only relevant regulations, but not specific and complete.  No more than 5 points.  Marking based on the security system provided by each CECSC when applying for rating. |
| C29  Degree of reward and punishment system perfection | Evaluating whether there are detailed, clear, operable reward and punishment management procedures and regulations | A scoring system is adopted, with a five-point system as the perfect score and a basic score of four points,  Five aspects including encouraging behavior, prohibited behavior, reward and punishment system, evaluation mechanism and feedback.  0.2 points for the detailed, clear and operable regulations in each area.  0.1 points if there are only relevant regulations, but not specific and complete.  No more than 5 points.  Marking based on the reward and punishment system provided by each CECSC when applying for rating. |
| C30  Degree of service process perfection | Evaluating whether there are detailed, clear, operable business management procedures and regulations | A scoring system is adopted, with a five-point system as the perfect score and a basic score of four points.  Five aspects of service reception, service execution, service evaluation and feedback, quality standards, and civilized standards are evaluated.  0.2 points for the detailed, clear and operable regulations in each area.  0.1 points if there are only relevant regulations, but not specific and complete.  No more than 5 points.  Marking according to the service process mechanism provided by each CECSC when applying for rating. |
| C31  Degree of emergency management perfection | Evaluating whether there are detailed, clear, operable emergency management procedures and regulations | A scoring system is adopted, with a five-point system as the perfect score and a basic score of four points.  Five aspects of emergency exit instructions, one-key alarm settings, fire equipment settings, emergency plan settings, and responsible personnel settings.  0.2 points for the detailed, clear and operable regulations in each area.  0.1 points if there are only relevant regulations, but not specific and complete.  No more than 5 points.  Marking according to the emergency handling process mechanism provided by each CECSC when applying for rating. |
| C32  Degree of complaint handling perfection | Evaluating whether there are detailed, clear, operable complaint management procedures and regulations | A scoring system is adopted, with a five-point system as the perfect score and a basic score of four points.  Five aspects of complaint reception, complaint transfer, complaint investigation, responsibility division, and feedback channels are evaluated.  0.2 points for the detailed, clear and operable regulations in each area.  0.1 points if there are only relevant regulations, but not specific and complete.  No more than 5 points.  Marking based on the complaint handling mechanism provided by each CECSC when applying for rating. |
| C33  Degree of Comments and Suggestions handling perfection | Evaluating whether there are detailed, clear, operable Comments and Suggestions management procedures and regulations | A scoring system is adopted, with a five-point system as the full score and a basic score of four points.  Five aspects of the reception of opinions and suggestions, verification of opinions and suggestions, feasibility investigation, comparison and selection of improvement plans, and feedback.  0.2 points for the detailed, clear and operable regulations in each area.  0.1 points if there are only relevant regulations, but not specific and complete.  No more than 5 points.  Marking based on the opinions and suggestions handling mechanism provided by each home care service center when applying for rating. |
