# Supplementary material for: A SBM-DEA based performance evaluation and optimization for social organizations participating in community and home-based elderly care services
Source: PLoS One. 2021 Mar 17;16(3):e0248474. doi: 10.1371/journal.pone.0248474 (PMC7968683; doi:10.1371/journal.pone.0248474)
Supplement: S2 Table — (DOCX) [file pone.0248474.s002.docx]

|  | **C1** | **C2** | **C3** | **C4** | **C5** | **C6** | **C7** | **C8** | **C9** | **C10** | **C11** | **C12** | **C13** | **C14** | **C15** | **C16** | **C17** | **C18** | **C19** | **C20** | **C21** | **C22** | **C23** | **C24** | **C25** | **C26** | **C27** | **C28** | **C29** | **C30** | **C31** | **C32** | **C33** |
| --- | --- | --- | --- | --- | --- | --- | --- | --- | --- | --- | --- | --- | --- | --- | --- | --- | --- | --- | --- | --- | --- | --- | --- | --- | --- | --- | --- | --- | --- | --- | --- | --- | --- |
| 1 | 100000 | 0 | 0 | 782225 | 1 | 0 | 800 | 10 | 50000 | 5 | 2 | 53455 | 44926 | 2 | 20 | 10 | 50 | 3 | 4.2 | 4.5 | 4.6 | 37 | 0.30 | 5 | 0 | 4.4 | 4.5 | 4.5 | 4.8 | 4.4 | 4.4 | 4.5 | 4.5 |
| 2 | 200000 | 0 | 0 | 244547 | 1 | 0 | 1000 | 5 | 100000 | 5 | 7 | 33783 | 8103 | 1 | 2 | 9 | 300 | 20 | 4.4 | 4.5 | 4.5 | 6 | 0.30 | 5 | 0 | 4.8 | 4.6 | 4.5 | 4.4 | 4.8 | 4.8 | 4.6 | 4.5 |
| 3 | 150000 | 0 | 0 | 74113 | 1 | 0 | 360 | 10 | 220000 | 5 | 8 | 6674 | 16116 | 1 | 3 | 1 | 30 | 5 | 4.5 | 4.4 | 4.8 | 45 | 0.05 | 4.8 | 0 | 4.5 | 4.5 | 4.5 | 4.5 | 4.4 | 4.5 | 4.5 | 4.5 |
| 4 | 23000 | 0 | 5000 | 314761 | 1 | 0 | 500 | 20 | 400000 | 1 | 5 | 20898 | 0 | 2 | 8 | 18 | 30 | 25 | 4.2 | 4.2 | 4.4 | 2 | 0.52 | 5 | 0 | 4.2 | 4.6 | 4.6 | 4.8 | 4.5 | 4.2 | 4.6 | 4.6 |
| 5 | 0 | 0 | 3000 | 74799 | 1 | 0 | 200 | 5 | 8000 | 0 | 2 | 5638 | 10939 | 1 | 2 | 10 | 16 | 16 | 4.3 | 4.5 | 4.7 | 30 | 0.10 | 5 | 0 | 4.8 | 4.5 | 4.6 | 4.5 | 4.9 | 4.8 | 4.5 | 4.6 |
| 6 | 100000 | 0 | 0 | 58370 | 1 | 0 | 120 | 10 | 150000 | 5 | 10 | 4990 | 10359 | 1 | 2 | 1 | 20 | 3 | 4.5 | 4.5 | 4.8 | 97 | 0.79 | 5 | 0 | 4.6 | 4.5 | 4.8 | 4.7 | 4.6 | 4.6 | 4.5 | 4.8 |
| 7 | 10000 | 0 | 0 | 8191 | 1 | 0 | 450 | 5 | 30000 | 5 | 5 | 464 | 5589 | 3 | 11 | 12 | 132 | 0 | 4.6 | 4.8 | 4.5 | 32 | 1.67 | 5 | 0 | 4.7 | 4.6 | 4.6 | 4.6 | 4.8 | 4.7 | 4.6 | 4.6 |
| 8 | 130000 | 60000 | 5000 | 8759 | 0 | 0 | 1500 | 10 | 6000 | 5 | 4 | 778 | 1545 | 2 | 3 | 12 | 46 | 0 | 4.6 | 4.8 | 4.2 | 28 | 0.04 | 5 | 0 | 4.3 | 4.5 | 4.6 | 4.9 | 4.8 | 4.3 | 4.5 | 4.6 |
| 9 | 60000 | 60000 | 5000 | 9087 | 0.5 | 0 | 1200 | 5 | 232000 | 5 | 1 | 591 | 0 | 1 | 2 | 10 | 24 | 2 | 4.2 | 4.4 | 4.6 | 44 | 0.04 | 5 | 0 | 4.4 | 4.7 | 4.5 | 4.8 | 4.9 | 4.4 | 4.7 | 4.5 |
| 10 | 0 | 0 | 0 | 222235 | 1 | 0 | 120 | 5 | 50000 | 5 | 3 | 14535 | 80377 | 2 | 3 | 7 | 13 | 6 | 4.5 | 4.7 | 4.2 | 41 | 0.42 | 5 | 0 | 4.5 | 4.8 | 4.8 | 4.8 | 4.6 | 4.5 | 4.8 | 4.8 |
| 11 | 0 | 0 | 0 | 126104 | 1 | 0 | 100 | 3 | 1000 | 5 | 3 | 13256 | 69540 | 1 | 1 | 8 | 46 | 7 | 4.4 | 4.5 | 4.5 | 3 | 0.06 | 5 | 0 | 4.3 | 4.8 | 4.6 | 4.8 | 4.4 | 4.3 | 4.8 | 4.6 |
| 12 | 100000 | 0 | 0 | 661663 | 1 | 0 | 900 | 28 | 1000000 | 5 | 0 | 45132 | 19278 | 1 | 3 | 0 | 30 | 0 | 4.8 | 4.6 | 4.5 | 0 | 0.34 | 4.6 | 0 | 4.5 | 4.7 | 4.4 | 4.4 | 4.8 | 4.5 | 4.7 | 4.4 |
| 13 | 0 | 0 | 0 | 139527 | 1 | 0 | 180 | 5 | 260000 | 0 | 2 | 9929 | 29664 | 1 | 2 | 0 | 30 | 0 | 4.5 | 4.5 | 4.5 | 0 | 0.22 | 5 | 0 | 4.8 | 4.5 | 4.9 | 4.7 | 4.5 | 4.8 | 4.5 | 4.9 |
| 14 | 0 | 0 | 0 | 187802 | 1 | 0 | 120 | 10 | 50000 | 0 | 2 | 18959 | 15806 | 3 | 6 | 9 | 20 | 0 | 4.2 | 4.6 | 4.6 | 0 | 0.22 | 5 | 0 | 4.9 | 4.5 | 4.8 | 4.8 | 4.8 | 4.9 | 4.5 | 4.8 |
| 15 | 80000 | 0 | 0 | 187438 | 1 | 0 | 300 | 5 | 80000 | 5 | 6 | 10960 | 20658 | 2 | 5 | 11 | 18 | 6 | 4.8 | 4.5 | 4.6 | 2 | 0.34 | 5 | 0 | 4.8 | 4.8 | 4.6 | 4.5 | 4.7 | 4.8 | 4.8 | 4.6 |
| 16 | 0 | 0 | 0 | 381481 | 1 | 0 | 600 | 10 | 0 | 5 | 3 | 17361 | 7336 | 2 | 4 | 3 | 30 | 0 | 4.6 | 4.5 | 4.8 | 1 | 0.30 | 5 | 1 | 4.8 | 4.5 | 4.8 | 4.4 | 4.9 | 4.8 | 4.5 | 4.8 |
| 17 | 0 | 0 | 70000 | 41060 | 1 | 0 | 270 | 5 | 300000 | 5 | 0 | 4315 | 15162 | 1 | 5 | 9 | 0 | 0 | 4.7 | 4.6 | 4.6 | 21 | 0.00 | 5 | 0 | 4.5 | 4.4 | 4.6 | 4.8 | 4.8 | 4.8 | 4.5 | 4.4 |
| 18 | 0 | 0 | 0 | 173930 | 1 | 1 | 220 | 5 | 0 | 5 | 2 | 15071 | 3918 | 1 | 1 | 2 | 2 | 0 | 4.3 | 4.5 | 4.6 | 4 | 0.08 | 5 | 0 | 4.8 | 4.5 | 4.4 | 4.8 | 4.9 | 4.4 | 4.5 | 4.5 |
| 19 | 200000 | 5000 | 0 | 244058 | 1 | 0 | 300 | 8 | 20000 | 5 | 3 | 17321 | 1070 | 1 | 4 | 4 | 25 | 3 | 4.4 | 4.7 | 4.5 | 0 | 0.61 | 5 | 0 | 4.4 | 4.5 | 4.7 | 4.8 | 4.8 | 4.2 | 4.4 | 4.8 |
| 20 | 0 | 0 | 0 | 46825 | 1 | 0 | 300 | 5 | 200000 | 5 | 2 | 4234 | 13804 | 1 | 3 | 1 | 10 | 5 | 4.5 | 4.8 | 4.8 | 0 | 0.54 | 4.6 | 1 | 4.2 | 4.7 | 4.5 | 4.5 | 4.8 | 4.5 | 4.2 | 4.5 |
| 21 | 0 | 0 | 0 | 147389 | 1 | 1 | 100 | 5 | 1000 | 0 | 2 | 8078 | 5855 | 1 | 1 | 7 | 100 | 7 | 4.3 | 4.8 | 4.6 | 2 | 0.07 | 5 | 0 | 4.5 | 4.8 | 4.8 | 4.5 | 4.5 | 4.6 | 4.8 | 4.7 |
| 22 | 100000 | 0 | 30000 | 85127 | 1 | 0 | 520 | 37 | 0 | 5 | 3 | 12161 | 0 | 2 | 6 | 0 | 30 | 0 | 4.5 | 4.7 | 4.4 | 55 | 0.12 | 5 | 0 | 4.5 | 4.8 | 4.8 | 4.5 | 4.2 | 4.5 | 4.4 | 4.5 |
| 23 | 105000 | 15000 | 0 | 171074 | 1 | 0 | 700 | 5 | 20000 | 5 | 3 | 10099 | 1739 | 5 | 5 | 11 | 30 | 0 | 4.8 | 4.5 | 4.9 | 2 | 0.00 | 5 | 0 | 4.8 | 4.8 | 4.6 | 4.5 | 4.5 | 4.4 | 4.5 | 4.4 |
| 24 | 100000 | 0 | 30000 | 57881 | 1 | 0 | 200 | 10 | 0 | 5 | 1 | 5185 | 6503 | 1 | 5 | 0 | 20 | 0 | 4.9 | 4.5 | 4.8 | 0 | 1.40 | 5 | 0 | 4.8 | 4.8 | 4.8 | 4.5 | 4.6 | 4.7 | 4.8 | 4.2 |
| 25 | 100000 | 0 | 30000 | 78322 | 1 | 0.5 | 1200 | 10 | 200000 | 5 | 4 | 5824 | 5180 | 2 | 12 | 12 | 20 | 3 | 4.8 | 4.8 | 4.6 | 0 | 0.09 | 5 | 0 | 4.4 | 4.6 | 4.5 | 4.4 | 4.8 | 4.5 | 4.5 | 4.5 |
| 26 | 0 | 0 | 0 | 39662 | 1 | 1 | 1000 | 10 | 190000 | 5 | 3 | 3994 | 6474 | 1 | 2 | 15 | 220 | 5 | 4.8 | 4.5 | 4.8 | 0 | 0.08 | 5 | 0 | 4.7 | 4.6 | 4.5 | 4.7 | 4.5 | 4.5 | 4.7 | 4.5 |
| 27 | 0 | 0 | 0 | 138016 | 1 | 1 | 466 | 45 | 120000 | 5 | 0 | 9489 | 28 | 3 | 28 | 0 | 0 | 4 | 4.5 | 4.4 | 4.6 | 192 | 0.00 | 5 | 0 | 4.5 | 4.5 | 4.5 | 4.8 | 4.8 | 4.4 | 4.5 | 4.8 |
| 28 | 60000 | 0 | 0 | 108911 | 1 | 0 | 120 | 5 | 0 | 5 | 3 | 5857 | 2986 | 1 | 6 | 11 | 1008 | 0 | 4.8 | 4.5 | 4.4 | 6 | 0.11 | 5 | 0 | 4.6 | 4.2 | 4.5 | 5 | 4.8 | 4.2 | 4.4 | 4.5 |
| 29 | 100000 | 0 | 60000 | 6918 | 0 | 0 | 600 | 5 | 100000 | 5 | 4 | 326 | 7528 | 1 | 2 | 5 | 50 | 4 | 4.4 | 4.5 | 4.7 | 47 | 0.14 | 5 | 0 | 4.5 | 4.3 | 4.5 | 4.8 | 4.6 | 4.5 | 4.2 | 4.4 |
| 30 | 0 | 0 | 10000 | 48911 | 1 | 0 | 80 | 5 | 5000 | 0 | 2 | 5350 | 2235 | 2 | 6 | 12 | 300 | 4 | 4.2 | 4.4 | 4.9 | 46 | 0.08 | 5 | 0 | 4.6 | 4.5 | 4.4 | 4.5 | 4.8 | 4.9 | 4.5 | 4.4 |
| 31 | 0 | 0 | 0 | 40243 | 1 | 0 | 190 | 10 | 0 | 5 | 2 | 3500 | 3963 | 1 | 2 | 3 | 0 | 0 | 4.5 | 4.2 | 4.7 | 23 | 0.04 | 5 | 0 | 4.5 | 4.4 | 4.7 | 4.4 | 4.6 | 4.6 | 4.8 | 4.5 |
| 32 | 0 | 0 | 0 | 35453 | 1 | 0 | 300 | 0 | 30000 | 0 | 2 | 2972 | 3152 | 2 | 5 | 10 | 20 | 3 | 4.6 | 4.8 | 4.4 | 10 | 0.04 | 5 | 0 | 4.5 | 4.5 | 4.8 | 4.5 | 4.4 | 4.4 | 4.5 | 4.9 |
| 33 | 30000 | 0 | 0 | 27037 | 1 | 0 | 240 | 10 | 1000 | 5 | 2 | 3115 | 2840 | 1 | 2 | 2 | 10 | 15 | 4.5 | 4.4 | 4.8 | 15 | 0.23 | 5 | 0 | 4.6 | 4.6 | 4.9 | 4.5 | 4.7 | 4.8 | 4.7 | 4.6 |
| 34 | 0 | 0 | 0 | 36508 | 1 | 0 | 200 | 5 | 1000 | 0 | 4 | 2903 | 2717 | 1 | 10 | 11 | 150 | 0 | 4.4 | 4.5 | 4.4 | 2 | 0.34 | 5 | 0 | 4.5 | 4.8 | 4.8 | 4.4 | 4.9 | 4.5 | 4.5 | 4.7 |
| 35 | 250000 | 8000 | 0 | 43634 | 1 | 0 | 200 | 8 | 5000 | 2 | 2 | 0 | 5017 | 2 | 4 | 2 | 12 | 5 | 4.7 | 4.8 | 4.5 | 0 | 0.54 | 5 | 0 | 4.7 | 4.8 | 4.5 | 4.2 | 4.7 | 4.8 | 4.4 | 4.9 |
| 36 | 30000 | 0 | 30000 | 2580 | 0 | 0 | 400 | 10 | 0 | 5 | 3 | 102 | 1735 | 1 | 2 | 0 | 30 | 0 | 4.5 | 4.5 | 4.9 | 12 | 2.53 | 4.8 | 0 | 4.8 | 4.5 | 4.4 | 4.8 | 4.4 | 4.7 | 4.2 | 4.7 |
| 37 | 0 | 0 | 0 | 5362 | 1 | 0 | 140 | 10 | 0 | 1 | 2 | 766 | 497 | 1 | 3 | 2 | 31 | 7 | 4.5 | 4.7 | 4.6 | 4 | 0.00 | 5 | 0 | 4.8 | 4.8 | 4.5 | 4.4 | 4.8 | 4.9 | 4.5 | 4.9 |
| 38 | 60000 | 0 | 0 | 1645 | 0 | 0 | 120 | 5 | 0 | 5 | 3 | 131 | 0 | 1 | 6 | 11 | 1008 | 0 | 4.6 | 4.6 | 4.8 | 6 | 0.11 | 5 | 0 | 4.7 | 4.4 | 4.5 | 4.5 | 4.4 | 4.8 | 4.5 | 4.8 |
| 39 | 400000 | 0 | 0 | 2180 | 0 | 0 | 600 | 10 | 1200000 | 5 | 1 | 109 | 0 | 8 | 97 | 0 | 200 | 10 | 4.5 | 4.9 | 4.8 | 600 | 0.02 | 5 | 0 | 4.5 | 4.2 | 4.4 | 4.8 | 4.5 | 4.9 | 4.8 | 4.6 |
| 40 | 635000 | 1000 | 0 | 250960 | 1 | 0 | 980 | 20 | 390000 | 5 | 11 | 23021 | 42063 | 5 | 12 | 3 | 98 | 18 | 5 | 4.8 | 4.9 | 880 | 0.07 | 5 | 0 | 4.9 | 4.8 | 4.8 | 4.5 | 4.9 | 4.8 | 4.6 | 4.8 |
| 41 | 270000 | 3000 | 0 | 212495 | 1 | 0 | 180 | 5 | 80000 | 5 | 8 | 17131 | 38984 | 2 | 6 | 3 | 22 | 10 | 4.5 | 4.8 | 4.6 | 880 | 0.06 | 5 | 0 | 4.8 | 4.6 | 4.8 | 4.7 | 4.6 | 4.8 | 4.4 | 4.6 |
| 42 | 100200 | 0 | 0 | 286669 | 1 | 0 | 120 | 5 | 177000 | 5 | 1 | 16123 | 30570 | 1 | 3 | 0 | 26 | 2 | 4.6 | 4.8 | 4.4 | 0 | 1.00 | 5 | 1 | 4.5 | 4.5 | 4.4 | 4.5 | 4.7 | 4.5 | 4.5 | 4.4 |
| 43 | 1538140 | 0 | 0 | 233156 | 1 | 0 | 8000 | 281 | 20000000 | 5 | 9 | 33308 | 942 | 30 | 100 | 1 | 20 | 0 | 4.5 | 4.4 | 4.8 | 55 | 0.00 | 5 | 0 | 4.4 | 4.4 | 4.5 | 4.4 | 4.9 | 4.2 | 4.2 | 4.4 |
| 44 | 30000 | 0 | 38000 | 64819 | 1 | 0 | 220 | 10 | 120000 | 5 | 5 | 5610 | 25254 | 3 | 8 | 2 | 20 | 5 | 4.4 | 4.7 | 4.5 | 48 | 0.00 | 5 | 0 | 4.5 | 4.7 | 4.8 | 4.2 | 4.7 | 4.5 | 4.5 | 4.7 |
| 45 | 0 | 0 | 0 | 144942 | 1 | 1 | 180 | 5 | 10000 | 5 | 0 | 17405 | 2326 | 3 | 3 | 1 | 16 | 10 | 4.5 | 4.8 | 4.8 | 0 | 0.00 | 5 | 0 | 4.5 | 4.5 | 4.5 | 4.5 | 4.9 | 4.6 | 4.5 | 4.8 |
| 46 | 0 | 0 | 0 | 118598 | 1 | 0 | 300 | 5 | 0 | 5 | 6 | 13997 | 3862 | 2 | 6 | 1 | 30 | 10 | 4.4 | 4.5 | 4.7 | 6 | 0.12 | 5 | 0 | 4.4 | 4.5 | 4.7 | 4.5 | 4.8 | 4.5 | 4.8 | 4.8 |
| 47 | 30000 | 0 | 0 | 84489 | 1 | 0 | 120 | 10 | 100000 | 5 | 5 | 6234 | 11193 | 3 | 5 | 2 | 12 | 3 | 4.2 | 4.4 | 4.9 | 40 | 0.00 | 4.8 | 0 | 4.2 | 4.4 | 4.5 | 4.8 | 4.6 | 4.4 | 4.5 | 4.7 |
| 48 | 30000 | 0 | 0 | 62722 | 1 | 0 | 120 | 10 | 100000 | 5 | 5 | 5353 | 11652 | 3 | 7 | 2 | 15 | 2 | 4.8 | 4.8 | 4.8 | 60 | 0.00 | 5 | 0 | 4.8 | 4.2 | 4.4 | 4.5 | 4.8 | 4.2 | 4.4 | 4.9 |
| 49 | 30000 | 0 | 0 | 102174 | 1 | 0 | 120 | 10 | 100000 | 5 | 4 | 6739 | 9770 | 3 | 4 | 2 | 15 | 2 | 4.6 | 4.8 | 4.9 | 30 | 0.00 | 5 | 0 | 4.9 | 4.5 | 4.8 | 4.4 | 4.6 | 4.8 | 4.8 | 4.8 |
| 50 | 0 | 0 | 0 | 106751 | 1 | 1 | 120 | 5 | 100000 | 5 | 5 | 10390 | 4603 | 2 | 10 | 2 | 20 | 15 | 4.5 | 4.8 | 4.8 | 11 | 0.15 | 5 | 0 | 4.8 | 4.8 | 4.6 | 4.5 | 4.4 | 4.6 | 4.8 | 4.9 |
| 51 | 0 | 0 | 0 | 253420 | 1 | 0 | 120 | 5 | 74100 | 5 | 5 | 9899 | 2757 | 4 | 4 | 2 | 2 | 2 | 4.6 | 4.5 | 4.8 | 0 | 0.00 | 5 | 0 | 4.8 | 4.5 | 4.8 | 4.2 | 4.4 | 4.5 | 4.8 | 4.8 |
| 52 | 0 | 0 | 0 | 75434 | 1 | 0 | 125 | 5 | 30000 | 5 | 5 | 5710 | 4383 | 3 | 28 | 8 | 60 | 10 | 4.6 | 4.5 | 4.5 | 0 | 0.22 | 5 | 0 | 4.5 | 4.4 | 4.6 | 4.5 | 4.7 | 4.6 | 4.5 | 4.8 |
| 53 | 0 | 0 | 0 | 73910 | 1 | 0 | 0 | 0 | 0 | 0 | 0 | 3634 | 5054 | 0 | 0 | 0 | 0 | 0 | 4.5 | 4.5 | 4.2 | 0 | 0.00 | 5 | 0 | 4.8 | 4.5 | 4.4 | 4.5 | 4.8 | 4.6 | 4.5 | 4.5 |
| 54 | 140000 | 0 | 0 | 148357 | 1 | 0 | 60 | 0 | 0 | 0 | 1 | 8062 | 22 | 2 | 2 | 9 | 100 | 0 | 4.2 | 4.5 | 4.5 | 0 | 0.00 | 5 | 0 | 4.2 | 4.2 | 4.4 | 4.8 | 4.5 | 4.5 | 4.5 | 4.2 |
| 55 | 0 | 0 | 0 | 12470 | 1 | 0 | 0 | 0 | 0 | 0 | 0 | 601 | 1531 | 0 | 0 | 0 | 0 | 0 | 4.3 | 4.5 | 4.6 | 0 | 0.00 | 5 | 0 | 4.3 | 4.5 | 4.7 | 4.8 | 4.2 | 4.2 | 4.5 | 4.5 |
| 56 | 270000 | 3000 | 0 | 10023 | 0 | 0 | 180 | 5 | 80000 | 5 | 8 | 525 | 732 | 2 | 6 | 3 | 22 | 10 | 4.5 | 4.4 | 4.8 | 880 | 0.06 | 5 | 0 | 4.5 | 4.5 | 4.8 | 4.5 | 4.5 | 4.3 | 4.5 | 4.6 |
| 57 | 0 | 0 | 0 | 19656 | 1 | 0 | 280 | 6 | 0 | 5 | 5 | 1183 | 1 | 1 | 4 | 1 | 12 | 3 | 4.4 | 4.7 | 4.5 | 2 | 0.04 | 5 | 0 | 4.6 | 4.8 | 4.5 | 4.4 | 4.8 | 4.4 | 4.6 | 4.6 |
| 58 | 80000 | 0 | 0 | 8382 | 1 | 1 | 150 | 5 | 1000000 | 5 | 1 | 878 | 0 | 2 | 4 | 1 | 1 | 1 | 4.5 | 4.8 | 4.8 | 100 | 1.00 | 5 | 0 | 4.6 | 4.8 | 4.2 | 4.2 | 4.4 | 4.7 | 4.2 | 4.4 |
| 59 | 3680000 | 0 | 0 | 6350051 | 1 | 0 | 780 | 10 | 0 | 5 | 10 | 257815 | 65014 | 20 | 318 | 7 | 150 | 1 | 5 | 5 | 4.8 | 900 | 0.00 | 5 | 0 | 4.9 | 5 | 4.9 | 4.8 | 4.7 | 4.8 | 4.8 | 4.9 |
| 60 | 0 | 0 | 0 | 1051290 | 1 | 0 | 80 | 4 | 0 | 1 | 0 | 146080 | 171 | 0 | 0 | 0 | 0 | 0 | 4.8 | 4.8 | 4.6 | 0 | 0.00 | 5 | 0 | 4.5 | 4.4 | 4.8 | 4.5 | 4.8 | 4.6 | 4.5 | 4.7 |
| 61 | 100000 | 0 | 0 | 689089 | 1 | 0 | 200 | 5 | 150000 | 5 | 2 | 51775 | 5142 | 1 | 3 | 2 | 30 | 0 | 4.8 | 4.5 | 4.8 | 0 | 0.23 | 5 | 0 | 4.2 | 4.2 | 4.4 | 4.8 | 4.5 | 4.5 | 4.5 | 4.8 |
| 62 | 0 | 0 | 0 | 708265 | 1 | 0 | 700 | 6 | 100000 | 5 | 2 | 37515 | 7643 | 1 | 2 | 0 | 0 | 0 | 4.5 | 4.4 | 4.6 | 0 | 0.00 | 4.8 | 1 | 4.3 | 4.5 | 4.7 | 4.8 | 4.2 | 4.6 | 4.6 | 4.5 |
| 63 | 0 | 0 | 0 | 391727 | 1 | 1 | 120 | 5 | 0 | 5 | 6 | 41325 | 2003 | 1 | 3 | 7 | 25 | 0 | 4.8 | 4.5 | 4.4 | 0 | 0.00 | 5 | 0 | 4.8 | 4.5 | 4.4 | 4.5 | 4.4 | 4.5 | 4.6 | 4.2 |
| 64 | 0 | 0 | 0 | 572422 | 1 | 0 | 70 | 5 | 0 | 5 | 6 | 31359 | 9128 | 1 | 3 | 9 | 10 | 0 | 4.4 | 4.5 | 4.7 | 0 | 0.00 | 5 | 0 | 4.4 | 4.5 | 4.7 | 4.8 | 4.5 | 4.5 | 4.7 | 4.5 |
| 65 | 5000 | 1300 | 0 | 277250 | 1 | 0 | 80 | 3 | 240000 | 0 | 2 | 38575 | 490 | 3 | 10 | 7 | 10 | 0 | 4.2 | 4.4 | 4.9 | 0 | 0.50 | 5 | 0 | 4.2 | 4.4 | 4.5 | 4.5 | 4.9 | 4.4 | 4.9 | 4.8 |
| 66 | 0 | 0 | 0 | 323147 | 1 | 0 | 130 | 6 | 0 | 5 | 4 | 17919 | 16015 | 1 | 2 | 8 | 30 | 3 | 4.5 | 4.2 | 4.7 | 0 | 0.07 | 5 | 0 | 4.5 | 4.2 | 4.5 | 4.7 | 4.6 | 4.2 | 4.7 | 4.4 |
| 67 | 0 | 0 | 0 | 306199 | 1 | 0 | 230 | 5 | 0 | 5 | 6 | 24134 | 7984 | 1 | 3 | 8 | 30 | 0 | 4.6 | 4.8 | 4.4 | 0 | 0.00 | 5 | 0 | 4.6 | 4.8 | 4.4 | 4.5 | 4.4 | 4.8 | 4.4 | 4.7 |
| 68 | 0 | 0 | 0 | 226274 | 1 | 2 | 300 | 8 | 500000 | 5 | 2 | 24750 | 2095 | 3 | 8 | 2 | 120 | 0 | 4.5 | 4.4 | 4.8 | 0 | 0.00 | 5 | 0 | 4.5 | 4.4 | 4.7 | 4.8 | 4.5 | 4.4 | 4.8 | 4.4 |
| 69 | 0 | 0 | 0 | 180713 | 1 | 0 | 120 | 4 | 0 | 5 | 7 | 15696 | 11072 | 0 | 37 | 3 | 26 | 0 | 4.4 | 4.5 | 4.4 | 0 | 0.65 | 5 | 0 | 4.4 | 4.5 | 4.5 | 4.5 | 4.9 | 4.5 | 4.4 | 4.7 |
| 70 | 0 | 0 | 0 | 298084 | 1 | 1 | 200 | 5 | 0 | 5 | 6 | 25030 | 1257 | 1 | 3 | 5 | 30 | 0 | 4.7 | 4.8 | 4.5 | 0 | 0.00 | 5 | 0 | 4.7 | 4.8 | 4.5 | 4.7 | 4.6 | 4.8 | 4.5 | 4.5 |
| 71 | 0 | 0 | 0 | 216360 | 1 | 0 | 100 | 4 | 10000 | 5 | 1 | 24013 | 1106 | 1 | 4 | 0 | 0 | 0 | 4.5 | 4.5 | 4.9 | 0 | 0.00 | 5 | 0 | 4.5 | 4.5 | 4.5 | 4.5 | 4.8 | 4.5 | 4.9 | 4.5 |
| 72 | 0 | 0 | 0 | 158282 | 1 | 1 | 180 | 10 | 50000 | 5 | 12 | 8949 | 14944 | 2 | 4 | 13 | 30 | 0 | 4.5 | 4.7 | 4.6 | 0 | 0.24 | 4.6 | 0 | 4.5 | 4.7 | 4.6 | 4.8 | 4.5 | 4.7 | 4.6 | 4.4 |
| 73 | 0 | 0 | 0 | 125187 | 1 | 0 | 380 | 10 | 800000 | 5 | 4 | 16530 | 7024 | 2 | 6 | 0 | 0 | 0 | 4.4 | 4.5 | 4.7 | 0 | 0.00 | 5 | 0 | 4.5 | 4.5 | 4.6 | 4.8 | 4.2 | 4.5 | 4.8 | 4.7 |
| 74 | 0 | 0 | 80000 | 125190 | 1 | 0 | 130 | 10 | 0 | 5 | 9 | 11775 | 10299 | 1 | 2 | 0 | 0 | 2 | 4.2 | 4.4 | 4.9 | 0 | 0.00 | 5 | 0 | 4.6 | 4.8 | 4.4 | 4.5 | 4.5 | 4.8 | 4.5 | 4.5 |
| 75 | 0 | 0 | 0 | 242511 | 1 | 1 | 90 | 5 | 0 | 5 | 6 | 17043 | 4582 | 1 | 3 | 9 | 10 | 0 | 4.5 | 4.2 | 4.7 | 0 | 0.00 | 5 | 0 | 4.6 | 4.8 | 4.5 | 4.4 | 4.8 | 4.8 | 4.2 | 4.5 |
| 76 | 0 | 0 | 0 | 276717 | 1 | 0 | 180 | 2 | 0 | 5 | 1 | 16593 | 4501 | 1 | 1 | 0 | 0 | 1 | 4.8 | 4.5 | 4.9 | 0 | 0.00 | 5 | 0 | 4.4 | 4.5 | 4.2 | 4.2 | 4.4 | 4.5 | 4.5 | 4.5 |
| 77 | 0 | 0 | 0 | 293855 | 1 | 0 | 200 | 5 | 0 | 5 | 6 | 16157 | 2902 | 1 | 3 | 8 | 28 | 0 | 4.9 | 4.5 | 4.8 | 0 | 0.00 | 5 | 0 | 4.5 | 4.4 | 4.3 | 4.5 | 4.7 | 4.4 | 4.8 | 4.6 |
| 78 | 168000 | 0 | 0 | 275568 | 1 | 1 | 600 | 9 | 0 | 5 | 2 | 16443 | 0 | 7 | 4 | 0 | 15 | 9 | 4.8 | 4.8 | 4.6 | 0 | 1.00 | 4.6 | 1 | 4.2 | 4.2 | 4.5 | 4.5 | 4.8 | 4.2 | 4.4 | 4.6 |
| 79 | 0 | 0 | 0 | 44870 | 1 | 0 | 200 | 6 | 10000 | 5 | 2 | 3648 | 11254 | 1 | 5 | 0 | 0 | 0 | 4.8 | 4.5 | 4.8 | 0 | 0.00 | 5 | 0 | 4.3 | 4.5 | 4.5 | 4.5 | 4.8 | 4.5 | 4.7 | 4.4 |
| 80 | 0 | 0 | 0 | 104327 | 1 | 0 | 90 | 3 | 29000 | 1 | 0 | 14318 | 315 | 2 | 3 | 0 | 0 | 2 | 4.5 | 4.4 | 4.6 | 0 | 0.00 | 5 | 0 | 4.5 | 4.5 | 4.6 | 4.8 | 4.5 | 4.6 | 4.8 | 4.5 |
| 81 | 0 | 0 | 0 | 198524 | 1 | 1 | 180 | 5 | 50000 | 5 | 6 | 13088 | 1318 | 5 | 40 | 0 | 0 | 30 | 4.8 | 4.5 | 4.4 | 253 | 0.00 | 5 | 0 | 4.6 | 4.8 | 4.6 | 4.8 | 4.2 | 4.5 | 4.4 | 4.2 |
| 82 | 0 | 0 | 0 | 72335 | 1 | 0 | 50 | 0 | 0 | 1 | 5 | 4934 | 8505 | 0 | 0 | 0 | 0 | 0 | 4.2 | 4.2 | 4.4 | 0 | 0.00 | 5 | 0 | 4.6 | 4.8 | 4.2 | 4.4 | 4.6 | 4.4 | 4.5 | 4.3 |
| 83 | 0 | 0 | 0 | 81753 | 1 | 0 | 300 | 2 | 600000 | 5 | 2 | 16135 | 1299 | 2 | 15 | 7 | 46 | 6 | 4.3 | 4.5 | 4.7 | 0 | 0.71 | 5 | 0 | 4.2 | 4.7 | 4.5 | 4.7 | 4.2 | 4.6 | 4.8 | 4.5 |
| 84 | 0 | 0 | 0 | 84143 | 1 | 1 | 55 | 0 | 0 | 1 | 3 | 6802 | 4164 | 0 | 0 | 0 | 0 | 0 | 4.5 | 4.5 | 4.8 | 0 | 0.00 | 5 | 0 | 4.5 | 4.9 | 4.4 | 4.5 | 4.5 | 4.5 | 4.4 | 4.5 |
| 85 | 5000 | 2500 | 0 | 10311 | 1 | 1 | 120 | 5 | 300000 | 5 | 7 | 1369 | 9562 | 2 | 2 | 10 | 28 | 0 | 4.6 | 4.8 | 4.5 | 0 | 0.87 | 5 | 0 | 4.5 | 4.8 | 4.8 | 4.6 | 4.5 | 4.4 | 4.5 | 4.6 |
| 86 | 200000 | 0 | 0 | 203521 | 1 | 0 | 155 | 6 | 260000 | 1 | 7 | 9055 | 1530 | 3 | 4 | 2 | 30 | 1 | 4.6 | 4.8 | 4.2 | 0 | 0.00 | 5 | 0 | 4.8 | 4.6 | 4.5 | 4.5 | 4.5 | 4.7 | 4.8 | 4.6 |
| 87 | 0 | 0 | 0 | 68887 | 1 | 0 | 50 | 0 | 0 | 1 | 5 | 10821 | 9754 | 0 | 0 | 0 | 0 | 0 | 4.4 | 4.5 | 4.5 | 0 | 0.00 | 5 | 0 | 4.5 | 4.8 | 4.2 | 4.6 | 4.6 | 4.5 | 4.5 | 4.2 |
| 88 | 0 | 0 | 0 | 71815 | 1 | 0 | 80 | 5 | 0 | 5 | 6 | 10247 | 0 | 1 | 3 | 9 | 26 | 0 | 4.5 | 4.4 | 4.8 | 0 | 0.00 | 5 | 0 | 4.4 | 4.6 | 4.5 | 4.7 | 4.8 | 4.5 | 4.7 | 4.5 |
| 89 | 0 | 0 | 0 | 156735 | 1 | 0 | 180 | 10 | 100000 | 5 | 6 | 10197 | 26 | 3 | 1 | 8 | 3 | 0 | 4.2 | 4.2 | 4.4 | 0 | 0.02 | 5 | 0 | 4.5 | 4.4 | 4.4 | 4.5 | 4.5 | 4.4 | 4.5 | 4.4 |
| 90 | 0 | 0 | 0 | 79984 | 1 | 1 | 1700 | 91 | 2000000 | 5 | 16 | 9154 | 0 | 6 | 19 | 11 | 0 | 5 | 4.3 | 4.5 | 4.7 | 0 | 0.00 | 5 | 0 | 4.2 | 4.4 | 4.2 | 4.5 | 4.7 | 4.7 | 4.8 | 4.8 |
| 91 | 100000 | 0 | 0 | 127616 | 1 | 0 | 300 | 10 | 310000 | 5 | 3 | 8453 | 0 | 1 | 5 | 2 | 50 | 1 | 4.5 | 4.5 | 4.8 | 0 | 0.24 | 5 | 0 | 4.5 | 4.7 | 4.8 | 4.4 | 4.5 | 4.5 | 4.5 | 4.5 |
| 92 | 0 | 0 | 0 | 190667 | 1 | 0 | 300 | 5 | 0 | 3 | 5 | 6666 | 1125 | 0 | 0 | 0 | 0 | 0 | 4.6 | 4.8 | 4.5 | 0 | 0.00 | 5 | 0 | 4.5 | 4.8 | 4.4 | 4.7 | 4.8 | 4.5 | 4.8 | 4.6 |
| 93 | 0 | 0 | 0 | 32871 | 1 | 0 | 200 | 10 | 60000 | 5 | 9 | 4642 | 2488 | 3 | 2 | 11 | 20 | 0 | 4.6 | 4.8 | 4.2 | 0 | 0.13 | 5 | 0 | 4.8 | 4.5 | 4.5 | 4.5 | 4.5 | 4.8 | 4.5 | 4.5 |
| 94 | 0 | 0 | 0 | 31614 | 1 | 0 | 120 | 2 | 30000 | 0 | 2 | 2895 | 366 | 2 | 1 | 2 | 10 | 1 | 4.2 | 4.4 | 4.6 | 0 | 0.13 | 5 | 0 | 4.8 | 4.2 | 4.8 | 4.5 | 4.7 | 4.8 | 4.2 | 4.2 |
| 95 | 100000 | 0 | 0 | 1253757 | 1 | 0 | 500 | 5 | 30000 | 5 | 6 | 60170 | 99403 | 1 | 3 | 3 | 10 | 1 | 4.5 | 4.7 | 4.2 | 12 | 1.00 | 5 | 0 | 4.5 | 4.5 | 4.5 | 4.5 | 4.5 | 4.5 | 4.5 | 4.3 |
| 96 | 0 | 0 | 0 | 746082 | 1 | 1 | 2500 | 6 | 100000 | 5 | 2 | 60849 | 59948 | 4 | 13 | 1 | 50 | 4 | 4.4 | 4.5 | 4.5 | 75 | 0.20 | 4.8 | 0 | 4.4 | 4.8 | 4.7 | 4.6 | 4.8 | 4.4 | 4.8 | 4.4 |
| 97 | 0 | 0 | 0 | 147457 | 1 | 0 | 400 | 5 | 100000 | 0 | 5 | 6452 | 95268 | 2 | 5 | 2 | 45 | 6 | 4.8 | 4.6 | 4.5 | 52 | 5.63 | 5 | 0 | 4.2 | 4.4 | 4.5 | 4.6 | 4.8 | 4.2 | 4.4 | 4.7 |
| 98 | 100000 | 0 | 0 | 599299 | 1 | 0 | 1000 | 5 | 20000 | 5 | 7 | 35094 | 63622 | 1 | 3 | 3 | 10 | 1 | 4.5 | 4.5 | 4.5 | 0 | 1.00 | 5 | 0 | 4.5 | 4.7 | 4.8 | 4.4 | 4.5 | 4.5 | 4.7 | 4.5 |
| 99 | 0 | 0 | 0 | 421764 | 1 | 0 | 500 | 6 | 100000 | 5 | 2 | 34541 | 62264 | 2 | 5 | 1 | 30 | 4 | 4.2 | 4.6 | 4.6 | 18 | 0.07 | 5 | 0 | 4.5 | 4.8 | 4.8 | 4.5 | 4.4 | 4.5 | 4.8 | 4.6 |
| 100 | 0 | 0 | 0 | 426947 | 1 | 0 | 300 | 5 | 150000 | 5 | 6 | 53608 | 2535 | 2 | 5 | 0 | 0 | 0 | 4.8 | 4.5 | 4.6 | 0 | 0.00 | 5 | 0 | 4.8 | 4.5 | 4.5 | 4.2 | 4.2 | 4.8 | 4.5 | 4.5 |
| 101 | 100000 | 0 | 0 | 352390 | 1 | 0 | 300 | 5 | 150000 | 5 | 6 | 41063 | 6383 | 2 | 5 | 0 | 0 | 0 | 4.4 | 4.5 | 4.7 | 0 | 0.00 | 5 | 0 | 4.8 | 4.2 | 4.4 | 4.3 | 4.5 | 4.8 | 4.2 | 4.6 |
| 102 | 0 | 0 | 0 | 21003 | 1 | 0 | 800 | 8 | 250000 | 1 | 2 | 2537 | 29670 | 4 | 6 | 0 | 0 | 0 | 4.2 | 4.4 | 4.9 | 0 | 0.02 | 4.7 | 2 | 4.4 | 4.6 | 4.2 | 4.5 | 4.5 | 4.5 | 4.4 | 4.5 |
| 103 | 0 | 0 | 0 | 144252 | 1 | 0 | 1500 | 6 | 350000 | 5 | 6 | 12510 | 4998 | 2 | 4 | 2 | 6 | 4 | 4.5 | 4.2 | 4.7 | 0 | 0.13 | 5 | 0 | 4.7 | 4.2 | 4.5 | 4.5 | 4.5 | 4.8 | 4.5 | 4.5 |
| 104 | 0 | 0 | 0 | 199501 | 1 | 0 | 2000 | 5 | 100000 | 5 | 5 | 8078 | 7938 | 2 | 2 | 0 | 4 | 0 | 4.6 | 4.8 | 4.4 | 29 | 0.35 | 5 | 0 | 4.5 | 4.8 | 4.5 | 4.6 | 4.8 | 4.5 | 4.9 | 4.4 |
| 105 | 80000 | 0 | 0 | 25781 | 1 | 0 | 150 | 6 | 130000 | 5 | 5 | 1525 | 9544 | 1 | 4 | 1 | 4 | 6 | 4.5 | 4.4 | 4.8 | 0 | 0.10 | 5 | 0 | 4.5 | 4.8 | 4.8 | 4.6 | 4.8 | 4.7 | 4.6 | 4.2 |
| 106 | 100000 | 0 | 0 | 60010 | 1 | 0 | 180 | 5 | 65000 | 5 | 4 | 2549 | 2122 | 2 | 2 | 2 | 5 | 1 | 4.4 | 4.5 | 4.4 | 0 | 0.02 | 5 | 0 | 4.8 | 4.5 | 4.8 | 4.2 | 4.4 | 4.5 | 4.4 | 4.8 |
| 107 | 100000 | 0 | 0 | 28226 | 1 | 0 | 227.2 | 6 | 10000 | 5 | 7 | 2190 | 1084 | 2 | 4 | 2 | 30 | 2 | 4.7 | 4.8 | 4.5 | 0 | 0.29 | 5 | 0 | 4.8 | 4.2 | 4.7 | 4.5 | 4.7 | 4.8 | 4.5 | 4.4 |
| 108 | 0 | 0 | 0 | 31373 | 1 | 0 | 305 | 5 | 50000 | 0 | 5 | 2800 | 451 | 3 | 3 | 3 | 10 | 0 | 4.5 | 4.5 | 4.9 | 2 | 0.73 | 5 | 0 | 4.4 | 4.6 | 4.5 | 4.7 | 4.6 | 4.5 | 4.9 | 4.5 |
| 109 | 100000 | 40000 | 0 | 30066 | 1 | 0 | 200 | 5 | 67000 | 5 | 2 | 2399 | 0 | 3 | 10 | 1 | 7 | 0 | 4.5 | 4.7 | 4.6 | 0 | 1.59 | 5 | 0 | 4.7 | 4.2 | 4.4 | 4.5 | 4.7 | 4.7 | 4.6 | 4.8 |
| 110 | 200000 | 0 | 0 | 9226 | 0 | 0 | 1200 | 5 | 30000 | 5 | 4 | 1318 | 0 | 1 | 2 | 2 | 48 | 0 | 4.5 | 4.5 | 4.8 | 100 | 0.63 | 5 | 0 | 4.5 | 4.5 | 4.2 | 4.4 | 4.9 | 4.5 | 4.8 | 4.5 |
| 111 | 50000 | 0 | 0 | 431113 | 1 | 0 | 400 | 8 | 800000 | 5 | 10 | 25686 | 80141 | 2 | 4 | 6 | 15 | 0 | 4.6 | 4.8 | 4.5 | 2 | 0.84 | 4.8 | 0 | 4.6 | 4.5 | 4.5 | 4.2 | 4.7 | 4.5 | 4.8 | 4.5 |
| 112 | 50000 | 0 | 0 | 270707 | 1 | 0 | 800 | 5 | 600000 | 5 | 10 | 14749 | 41744 | 2 | 4 | 4 | 15 | 0 | 4.6 | 4.8 | 4.2 | 18 | 0.86 | 5 | 0 | 4.5 | 4.5 | 4.8 | 4.5 | 4.9 | 4.8 | 4.8 | 4.2 |
| 113 | 210000 | 0 | 0 | 277227 | 1 | 1 | 240 | 10 | 100000 | 5 | 9 | 17781 | 14129 | 2 | 3 | 10 | 20 | 3 | 4.4 | 4.5 | 4.5 | 0 | 0.64 | 5 | 0 | 4.6 | 4.6 | 4.9 | 4.5 | 4.8 | 4.4 | 4.5 | 4.5 |
| 114 | 0 | 0 | 0 | 241345 | 1 | 2 | 350 | 10 | 1000 | 5 | 11 | 21342 | 6176 | 3 | 2 | 7 | 10 | 4 | 4.5 | 4.4 | 4.8 | 8 | 1.00 | 5 | 0 | 4.7 | 4.8 | 4.8 | 4.8 | 4.6 | 4.7 | 4.4 | 4.8 |
| 115 | 0 | 0 | 100000 | 221439 | 1 | 0 | 140 | 5 | 30000 | 5 | 6 | 21228 | 3344 | 1 | 1 | 2 | 15 | 0 | 4.2 | 4.2 | 4.4 | 0 | 1.00 | 5 | 0 | 4.5 | 4.5 | 4.8 | 4.5 | 4.8 | 4.8 | 4.2 | 4.4 |
| 116 | 100000 | 0 | 0 | 56102 | 1 | 0 | 300 | 5 | 1500 | 5 | 9 | 5394 | 15650 | 1 | 1 | 1 | 18 | 0 | 4.3 | 4.5 | 4.7 | 4 | 3.06 | 5 | 0 | 4.5 | 4.7 | 4.5 | 4.4 | 4.6 | 4.8 | 4.5 | 4.7 |
| 117 | 100000 | 0 | 0 | 53331 | 1 | 0 | 200 | 5 | 1600 | 5 | 2 | 5573 | 15423 | 1 | 1 | 1 | 15 | 0 | 4.5 | 4.5 | 4.8 | 3 | 2.04 | 4.7 | 0 | 4.4 | 4.5 | 4.8 | 4.5 | 4.4 | 4.5 | 4.6 | 4.8 |
| 118 | 20000 | 0 | 0 | 144181 | 1 | 1 | 120 | 5 | 100000 | 3 | 4 | 13748 | 6493 | 2 | 3 | 1 | 20 | 0 | 4.6 | 4.8 | 4.5 | 0 | 0.24 | 5 | 0 | 4.7 | 4.8 | 4.2 | 4.2 | 4.4 | 4.2 | 4.5 | 4.4 |
| 119 | 30000 | 0 | 0 | 283043 | 1 | 0 | 50 | 5 | 80000 | 5 | 11 | 16646 | 1508 | 2 | 4 | 6 | 20 | 0 | 4.6 | 4.8 | 4.2 | 9 | 0.68 | 5 | 0 | 4.5 | 4.5 | 4.3 | 4.5 | 4.7 | 4.6 | 4.4 | 4.5 |
| 120 | 0 | 0 | 0 | 68793 | 1 | 0 | 500 | 10 | 2000 | 5 | 7 | 4340 | 13703 | 3 | 4 | 3 | 30 | 0 | 4.2 | 4.4 | 4.6 | 40 | 0.86 | 5 | 0 | 4.5 | 4.7 | 4.5 | 4.5 | 4.8 | 4.2 | 4.6 | 4.8 |
| 121 | 0 | 0 | 0 | 111039 | 1 | 0 | 220 | 10 | 500000 | 5 | 6 | 14547 | 3217 | 3 | 6 | 0 | 5 | 0 | 4.5 | 4.7 | 4.2 | 0 | 0.78 | 5 | 0 | 4.5 | 4.5 | 4.6 | 4.8 | 4.5 | 4.5 | 4.5 | 4.4 |
| 122 | 130000 | 0 | 0 | 124010 | 1 | 0 | 800 | 10 | 260000 | 5 | 9 | 11241 | 5393 | 2 | 2 | 3 | 25 | 2 | 4.5 | 4.2 | 4.7 | 4 | 1.00 | 5 | 0 | 4.6 | 4.8 | 4.6 | 4.8 | 4.2 | 4.5 | 4.4 | 4.5 |
| 123 | 50000 | 0 | 0 | 42252 | 1 | 0 | 120 | 5 | 40000 | 5 | 11 | 4049 | 11836 | 1 | 3 | 4 | 12 | 0 | 4.6 | 4.8 | 4.4 | 11 | 0.79 | 5 | 0 | 4.6 | 4.8 | 4.4 | 4.5 | 4.5 | 4.5 | 4.7 | 4.8 |
| 124 | 50000 | 0 | 0 | 86180 | 1 | 0 | 160 | 5 | 50000 | 5 | 11 | 8450 | 7128 | 2 | 5 | 3 | 65 | 6 | 4.5 | 4.4 | 4.8 | 13 | 0.97 | 5 | 0 | 4.4 | 4.5 | 4.5 | 4.4 | 4.8 | 4.6 | 4.5 | 4.5 |
| 125 | 80000 | 0 | 0 | 116170 | 1 | 0 | 160 | 10 | 30000 | 5 | 6 | 10131 | 5122 | 2 | 4 | 2 | 9 | 1 | 4.4 | 4.5 | 4.4 | 13 | 0.00 | 4.8 | 1 | 4.5 | 4.4 | 4.2 | 4.2 | 4.4 | 4.8 | 4.5 | 4.7 |
| 126 | 50000 | 0 | 0 | 75232 | 1 | 0 | 50 | 5 | 30000 | 5 | 7 | 5839 | 8750 | 1 | 3 | 3 | 8 | 0 | 4.6 | 4.8 | 4.4 | 19 | 0.54 | 5 | 0 | 4.2 | 4.2 | 4.3 | 4.5 | 4.7 | 4.5 | 4.4 | 4.5 |
| 127 | 50000 | 0 | 0 | 48316 | 1 | 0 | 80 | 2 | 20000 | 5 | 11 | 4178 | 9769 | 2 | 2 | 2 | 1 | 1 | 4.5 | 4.4 | 4.8 | 10 | 0.46 | 5 | 0 | 4.3 | 4.5 | 4.5 | 4.5 | 4.8 | 4.7 | 4.7 | 4.8 |
| 128 | 50000 | 0 | 0 | 60657 | 1 | 0 | 100 | 5 | 1000 | 5 | 0 | 3939 | 9707 | 1 | 1 | 1 | 10 | 0 | 4.4 | 4.5 | 4.4 | 0 | 0.00 | 5 | 0 | 4.5 | 4.5 | 4.6 | 4.8 | 4.5 | 4.5 | 4.5 | 4.5 |
| 129 | 100000 | 0 | 0 | 36289 | 1 | 0 | 180 | 10 | 1200 | 5 | 9 | 3769 | 9222 | 1 | 1 | 1 | 15 | 0 | 4.7 | 4.8 | 4.5 | 2 | 3.88 | 5 | 0 | 4.5 | 4.5 | 4.6 | 4.8 | 4.2 | 4.5 | 4.5 | 4.5 |
| 130 | 30000 | 0 | 0 | 51043 | 1 | 0 | 30 | 2 | 30000 | 5 | 11 | 4220 | 8529 | 1 | 2 | 3 | 3 | 0 | 4.5 | 4.5 | 4.9 | 23 | 0.61 | 5 | 0 | 4.5 | 4.7 | 4.2 | 4.4 | 4.6 | 4.4 | 4.8 | 4.5 |
| 131 | 0 | 0 | 100000 | 68741 | 1 | 0 | 440 | 5 | 170000 | 5 | 6 | 5813 | 6383 | 1 | 5 | 8 | 15 | 6 | 4.5 | 4.7 | 4.6 | 0 | 0.40 | 5 | 0 | 4.4 | 4.5 | 4.5 | 4.7 | 4.2 | 4.2 | 4.4 | 4.8 |
| 132 | 30000 | 0 | 0 | 157634 | 1 | 0 | 40 | 2 | 20000 | 5 | 11 | 6086 | 5569 | 1 | 2 | 2 | 2 | 2 | 4.4 | 4.5 | 4.4 | 0 | 0.69 | 5 | 0 | 4.7 | 4.8 | 4.4 | 4.5 | 4.5 | 4.5 | 4.7 | 4.8 |
| 133 | 50000 | 0 | 0 | 117103 | 1 | 0 | 160 | 0 | 20000 | 0 | 3 | 4560 | 4406 | 1 | 9 | 10 | 15 | 0 | 4.7 | 4.8 | 4.5 | 0 | 0.01 | 5 | 0 | 4.5 | 4.5 | 4.8 | 4.6 | 4.5 | 4.5 | 4.4 | 4.5 |
| 134 | 30000 | 0 | 0 | 88962 | 1 | 0 | 60 | 2 | 10000 | 5 | 11 | 6220 | 168 | 2 | 4 | 3 | 2 | 0 | 4.5 | 4.5 | 4.9 | 5 | 0.55 | 5 | 0 | 4.5 | 4.5 | 4.5 | 4.5 | 4.5 | 4.5 | 4.7 | 4.8 |
| 135 | 0 | 50000 | 100000 | 45349 | 1 | 1 | 330 | 10 | 150000 | 5 | 4 | 5523 | 517 | 3 | 10 | 2 | 10 | 2 | 4.5 | 4.7 | 4.6 | 0 | 0.00 | 4.7 | 0 | 4.8 | 4.5 | 4.2 | 4.6 | 4.6 | 4.4 | 4.5 | 4.5 |
| 136 | 30000 | 0 | 0 | 15651 | 1 | 0 | 100 | 5 | 10000 | 5 | 11 | 1844 | 1236 | 2 | 2 | 3 | 4 | 0 | 4.5 | 4.5 | 4.8 | 13 | 0.39 | 5 | 0 | 4.4 | 4.8 | 4.8 | 4.5 | 4.6 | 4.2 | 4.5 | 4.7 |
| 137 | 0 | 0 | 0 | 15303 | 1 | 0 | 220 | 10 | 5000 | 0 | 4 | 1937 | 344 | 4 | 6 | 0 | 5 | 4 | 4.6 | 4.8 | 4.5 | 3 | 0.27 | 5 | 0 | 4.7 | 4.8 | 4.4 | 4.5 | 4.7 | 4.8 | 4.4 | 4.5 |
| 138 | 0 | 0 | 0 | 4150 | 1 | 0 | 120 | 5 | 0 | 5 | 6 | 188 | 1688 | 2 | 2 | 3 | 10 | 8 | 4.6 | 4.8 | 4.2 | 2 | 0.00 | 5 | 0 | 4.4 | 4.5 | 4.5 | 4.7 | 4.6 | 4.4 | 4.7 | 4.8 |
| 139 | 0 | 0 | 0 | 29455 | 1 | 0 | 180 | 5 | 150000 | 0 | 5 | 1417 | 0 | 4 | 13 | 1 | 13 | 0 | 4.4 | 4.5 | 4.5 | 0 | 0.00 | 5 | 0 | 4.7 | 4.8 | 4.5 | 4.8 | 4.2 | 4.5 | 4.5 | 4.5 |
| 140 | 0 | 0 | 0 | 4926 | 1 | 0 | 100 | 5 | 10000 | 5 | 6 | 492 | 911 | 3 | 8 | 5 | 10 | 7 | 4.5 | 4.4 | 4.8 | 0 | 0.00 | 5 | 0 | 4.5 | 4.5 | 4.6 | 4.5 | 4.5 | 4.8 | 4.5 | 4.7 |
| 141 | 5000 | 0 | 0 | 1884 | 1 | 0 | 1000 | 5 | 31000 | 5 | 7 | 258 | 0 | 2 | 2 | 3 | 46 | 3 | 4.2 | 4.2 | 4.4 | 0 | 1.14 | 5 | 0 | 4.5 | 4.7 | 4.6 | 4.2 | 4.5 | 4.5 | 4.5 | 4.5 |
| 142 | 260000 | 0 | 0 | 127417 | 1 | 2 | 120 | 10 | 300000 | 5 | 1 | 12789 | 20173 | 1 | 3 | 0 | 5 | 0 | 4.3 | 4.5 | 4.7 | 6 | 0.05 | 4.8 | 0 | 4.4 | 4.5 | 4.7 | 4.5 | 4.5 | 4.7 | 4.6 | 4.8 |
| 143 | 290000 | 0 | 0 | 77824 | 1 | 0 | 810 | 10 | 100000 | 5 | 1 | 7139 | 24055 | 2 | 6 | 2 | 32 | 0 | 4.5 | 4.5 | 4.8 | 29 | 0.44 | 4.6 | 1 | 4.5 | 4.5 | 4.3 | 4.8 | 4.6 | 4.5 | 4.6 | 4.8 |
| 144 | 190000 | 0 | 0 | 102646 | 1 | 0 | 380 | 10 | 50000 | 5 | 1 | 11958 | 18688 | 1 | 2 | 5 | 10 | 2 | 4.6 | 4.8 | 4.5 | 4 | 0.29 | 5 | 0 | 4.5 | 4.7 | 4.5 | 4.5 | 4.8 | 4.6 | 4.4 | 4.5 |
| 145 | 60000 | 0 | 0 | 105533 | 1 | 0 | 120 | 5 | 200000 | 0 | 1 | 13824 | 11061 | 2 | 3 | 3 | 5 | 3 | 4.4 | 4.5 | 4.7 | 2 | 0.35 | 5 | 0 | 4.5 | 4.5 | 4.6 | 4.8 | 4.5 | 4.2 | 4.7 | 4.8 |
| 146 | 100000 | 0 | 0 | 63460 | 1 | 0 | 3047 | 66 | 2000000 | 5 | 1 | 3173 | 13876 | 2 | 5 | 2 | 20 | 2 | 4.2 | 4.4 | 4.9 | 0 | 0.12 | 5 | 0 | 4.6 | 4.8 | 4.6 | 4.8 | 4.2 | 4.5 | 4.5 | 4.5 |
| 147 | 3090000 | 0 | 150000 | 58537 | 0 | 0 | 720 | 10 | 3000000 | 0 | 1 | 5330 | 11297 | 2 | 3 | 3 | 12 | 0 | 4.5 | 4.2 | 4.7 | 1 | 0.39 | 5 | 0 | 4.6 | 4.8 | 4.4 | 4.5 | 4.5 | 4.4 | 4.5 | 4.7 |
| 148 | 200000 | 0 | 0 | 59045 | 1 | 0 | 240 | 10 | 5000 | 5 | 1 | 6095 | 10280 | 1 | 1 | 1 | 0 | 1 | 4.6 | 4.8 | 4.4 | 0 | 0.01 | 5 | 0 | 4.4 | 4.5 | 4.5 | 4.4 | 4.8 | 4.8 | 4.4 | 4.5 |
| 149 | 180000 | 0 | 0 | 133029 | 1 | 0 | 200 | 5 | 120000 | 5 | 1 | 6889 | 9012 | 1 | 2 | 1 | 25 | 1 | 4.5 | 4.4 | 4.8 | 5 | 0.00 | 4.7 | 0 | 4.5 | 4.4 | 4.2 | 4.2 | 4.4 | 4.5 | 4.5 | 4.5 |
| 150 | 530000 | 0 | 0 | 51366 | 0.5 | 0 | 500 | 5 | 1500000 | 0 | 1 | 5190 | 10418 | 2 | 4 | 1 | 20 | 2 | 4.5 | 4.8 | 4.8 | 6 | 0.04 | 5 | 0 | 4.2 | 4.2 | 4.3 | 4.5 | 4.7 | 4.2 | 4.5 | 4.7 |
| 151 | 0 | 5000 | 0 | 48802 | 1 | 0 | 100 | 5 | 30000 | 0 | 1 | 5504 | 10038 | 1 | 2 | 3 | 2 | 1 | 4.4 | 4.4 | 4.5 | 0 | 0.00 | 5 | 0 | 4.3 | 4.5 | 4.5 | 4.5 | 4.8 | 4.8 | 4.5 | 4.5 |
| 152 | 530000 | 0 | 220000 | 143200 | 1 | 0 | 260 | 10 | 1220000 | 5 | 1 | 10884 | 4586 | 8 | 10 | 1 | 5 | 2 | 4.6 | 4.4 | 4.7 | 3 | 0.29 | 5 | 0 | 4.5 | 4.5 | 4.6 | 4.8 | 4.5 | 4.4 | 4.6 | 4.8 |
| 153 | 340000 | 0 | 0 | 77552 | 1 | 1 | 500 | 10 | 300000 | 5 | 5 | 6349 | 7364 | 2 | 5 | 1 | 100 | 5 | 4.5 | 4.8 | 4.5 | 0 | 1.12 | 5 | 0 | 4.5 | 4.5 | 4.6 | 4.8 | 4.2 | 4.2 | 4.6 | 4.8 |
| 154 | 230000 | 0 | 30000 | 273127 | 1 | 0 | 140 | 5 | 60000 | 5 | 1 | 10733 | 416 | 2 | 5 | 5 | 30 | 3 | 4.4 | 4.4 | 4.8 | 2 | 0.64 | 5 | 0 | 4.6 | 4.8 | 4.2 | 4.4 | 4.6 | 4.5 | 4.4 | 4.5 |
| 155 | 119200 | 0 | 0 | 73710 | 1 | 0 | 200 | 5 | 5000 | 0 | 1 | 10530 | 0 | 1 | 4 | 4 | 3 | 3 | 4.7 | 4.5 | 4.8 | 4 | 0.85 | 5 | 0 | 4.6 | 4.8 | 4.5 | 4.7 | 4.2 | 4.6 | 4.5 | 4.4 |
| 156 | 360000 | 0 | 0 | 78319 | 1 | 0 | 200 | 5 | 2000000 | 5 | 1 | 9459 | 560 | 2 | 4 | 1 | 15 | 2 | 4.5 | 4.9 | 4.5 | 5 | 0.05 | 5 | 0 | 4.2 | 4.4 | 4.4 | 4.5 | 4.5 | 4.5 | 4.2 | 4.2 |
| 157 | 0 | 5000 | 0 | 21979 | 1 | 0 | 100 | 5 | 30000 | 0 | 1 | 1986 | 5657 | 1 | 2 | 3 | 2 | 1 | 4.6 | 4.8 | 4.4 | 0 | 0.00 | 5 | 0 | 4.5 | 4.6 | 4.2 | 4.5 | 4.7 | 4.4 | 4.3 | 4.5 |
| 158 | 150000 | 0 | 150000 | 62185 | 1 | 0 | 270 | 10 | 1500000 | 5 | 1 | 4595 | 2817 | 2 | 2 | 1 | 15 | 0 | 4.7 | 4.5 | 4.4 | 4 | 0.88 | 5 | 0 | 4.5 | 4.7 | 4.8 | 4.4 | 4.5 | 4.7 | 4.5 | 4.5 |
| 159 | 170000 | 0 | 0 | 23910 | 1 | 0 | 240 | 5 | 480000 | 5 | 1 | 1086 | 6093 | 1 | 3 | 1 | 16 | 0 | 4.4 | 4.6 | 4.6 | 0 | 0.45 | 5 | 0 | 4.7 | 4.6 | 4.4 | 4.7 | 4.8 | 4.5 | 4.5 | 4.5 |
| 160 | 60000 | 0 | 0 | 2121 | 0 | 0 | 300 | 5 | 0 | 0 | 1 | 303 | 1753 | 1 | 3 | 1 | 300 | 0 | 4.8 | 4.6 | 4.5 | 6 | 0.76 | 4.7 | 0 | 4.8 | 4.2 | 4.5 | 4.5 | 4.5 | 4.5 | 4.6 | 4.8 |
| 161 | 60000 | 0 | 0 | 11817 | 1 | 0 | 0 | 5 | 0 | 0 | 1 | 1079 | 975 | 0 | 0 | 0 | 0 | 0 | 4.8 | 4.4 | 4.4 | 0 | 0.00 | 5 | 0 | 4.5 | 4.5 | 4.8 | 4.5 | 4.7 | 4.5 | 4.6 | 4.8 |
| 162 | 180000 | 0 | 200000 | 11452 | 0 | 0 | 175 | 10 | 500000 | 5 | 1 | 1546 | 0 | 2 | 6 | 6 | 50 | 0 | 4.5 | 4.5 | 4.7 | 3 | 0.05 | 5 | 0 | 4.2 | 4.5 | 4.5 | 4.5 | 4.5 | 4.6 | 4.2 | 4.4 |
| 163 | 270000 | 0 | 0 | 6001 | 0 | 0 | 110 | 5 | 100000 | 5 | 1 | 796 | 0 | 2 | 2 | 2 | 5 | 1 | 4.7 | 4.2 | 4.5 | 10 | 0.93 | 5 | 0 | 4.5 | 4.5 | 4.7 | 4.6 | 4.8 | 4.6 | 4.5 | 4.6 |
| 164 | 0 | 0 | 0 | 531326 | 1 | 0 | 150 | 10 | 80000 | 5 | 0 | 44599 | 3549 | 1 | 8 | 1 | 15 | 2 | 4.5 | 4.3 | 4.8 | 13 | 0.32 | 5 | 0 | 4.8 | 4.6 | 4.5 | 4.6 | 4.8 | 4.4 | 4.5 | 4.7 |
| 165 | 50000 | 0 | 0 | 35832 | 1 | 0 | 100 | 8 | 5000 | 0 | 2 | 1801 | 20827 | 2 | 10 | 10 | 45 | 0 | 4.8 | 4.5 | 4.5 | 1 | 0.15 | 5 | 0 | 4.5 | 4.8 | 4.6 | 4.7 | 4.6 | 4.5 | 4.7 | 4.6 |
| 166 | 0 | 0 | 0 | 94463 | 1 | 0 | 260 | 11 | 10000 | 5 | 3 | 4729 | 15585 | 1 | 2 | 14 | 45 | 2 | 4.8 | 4.6 | 4.7 | 4 | 0.14 | 5 | 0 | 4.8 | 4.5 | 4.2 | 4.5 | 4.6 | 4.2 | 4.8 | 4.2 |
| 167 | 0 | 0 | 0 | 90236 | 1 | 0 | 100 | 5 | 20000 | 0 | 2 | 3538 | 12131 | 2 | 1 | 3 | 40 | 0 | 4.4 | 4.4 | 4.5 | 0 | 0.00 | 5 | 0 | 4.8 | 4.2 | 4.5 | 4.6 | 4.8 | 4.3 | 4.5 | 4.5 |
| 168 | 0 | 0 | 0 | 102966 | 1 | 0 | 130 | 10 | 50000 | 5 | 0 | 13632 | 415 | 1 | 4 | 1 | 10 | 2 | 4.7 | 4.2 | 4.8 | 8 | 0.37 | 5 | 0 | 4.5 | 4.5 | 4.4 | 4.2 | 4.4 | 4.5 | 4.2 | 4.5 |
| 169 | 0 | 0 | 0 | 58564 | 1 | 0 | 400 | 5 | 30000 | 0 | 2 | 7590 | 559 | 1 | 2 | 0 | 10 | 2 | 4.5 | 4.5 | 4.8 | 10 | 0.22 | 4.6 | 0 | 4.4 | 4.8 | 4.8 | 4.5 | 4.7 | 4.6 | 4.5 | 4.5 |
| 170 | 0 | 0 | 0 | 123669 | 1 | 0 | 370 | 5 | 100000 | 5 | 1 | 8829 | 20692 | 2 | 4 | 6 | 20 | 4 | 4.5 | 4.6 | 4.5 | 10 | 0.28 | 5 | 0 | 4.2 | 4.4 | 4.5 | 4.4 | 4.5 | 4.6 | 4.8 | 4.5 |
| 171 | 0 | 0 | 0 | 56642 | 1 | 0 | 510 | 5 | 120000 | 5 | 1 | 3598 | 21035 | 2 | 6 | 4 | 20 | 0 | 4.5 | 4.5 | 4.4 | 4 | 0.25 | 5 | 0 | 4.5 | 4.7 | 4.2 | 4.8 | 4.6 | 4.2 | 4.5 | 4.6 |
| 172 | 0 | 0 | 0 | 84563 | 1 | 0 | 120 | 5 | 400000 | 0 | 1 | 8860 | 4865 | 2 | 17 | 1 | 30 | 1 | 4.6 | 4.5 | 4.2 | 17 | 0.19 | 5 | 0 | 4.5 | 4.8 | 4.8 | 4.5 | 4.5 | 4.5 | 4.8 | 4.6 |
| 173 | 0 | 0 | 0 | 114738 | 1 | 0 | 400 | 10 | 1000000 | 5 | 1 | 8401 | 4203 | 2 | 3 | 0 | 25 | 0 | 4.6 | 4.4 | 4.4 | 26 | 0.19 | 5 | 0 | 4.8 | 4.5 | 4.4 | 4.2 | 4.6 | 4.5 | 4.8 | 4.2 |
| 174 | 0 | 0 | 0 | 95678 | 1 | 0 | 650 | 30 | 420000 | 5 | 1 | 8848 | 3431 | 2 | 8 | 2 | 49 | 5 | 4.4 | 4.5 | 4.6 | 15 | 0.19 | 5 | 0 | 4.8 | 4.2 | 4.2 | 4.8 | 4.5 | 4.6 | 4.8 | 4.5 |
| 175 | 0 | 0 | 0 | 71310 | 1 | 0 | 300 | 5 | 450000 | 5 | 1 | 5152 | 6342 | 2 | 12 | 2 | 30 | 1 | 4.5 | 4.9 | 4.5 | 7 | 0.20 | 4.8 | 0 | 4.4 | 4.6 | 4.5 | 4.4 | 4.5 | 4.5 | 4.4 | 4.5 |
| 176 | 0 | 0 | 0 | 21354 | 1 | 0 | 220 | 3 | 20000 | 5 | 1 | 2739 | 7832 | 1 | 6 | 7 | 15 | 2 | 4.9 | 4.5 | 4.4 | 6 | 0.04 | 5 | 0 | 4.7 | 4.2 | 4.6 | 4.2 | 4.4 | 4.4 | 4.5 | 4.7 |
| 177 | 0 | 0 | 0 | 67995 | 1 | 0 | 350 | 5 | 350000 | 5 | 1 | 6578 | 3813 | 2 | 20 | 2 | 30 | 1 | 4.8 | 4.7 | 4.7 | 24 | 0.10 | 5 | 0 | 4.5 | 4.5 | 4.5 | 4.5 | 4.2 | 4.6 | 4.8 | 4.8 |
| 178 | 0 | 0 | 0 | 68966 | 1 | 0 | 265 | 5 | 307300 | 5 | 1 | 6318 | 3783 | 2 | 15 | 2 | 30 | 1 | 4.5 | 4.5 | 4.5 | 8 | 0.10 | 5 | 0 | 4.2 | 4.4 | 4.5 | 4.6 | 4.8 | 4.5 | 4.4 | 4.5 |
| 179 | 0 | 0 | 0 | 49666 | 1 | 0 | 450 | 10 | 600000 | 5 | 1 | 5700 | 2686 | 2 | 20 | 2 | 50 | 2 | 4.6 | 4.8 | 4.8 | 20 | 0.19 | 5 | 0 | 4.5 | 4.7 | 4.2 | 4.5 | 4.4 | 4.4 | 4.5 | 4.2 |
| 180 | 0 | 0 | 0 | 55977 | 1 | 0 | 472 | 11 | 450000 | 5 | 1 | 5300 | 3029 | 2 | 6 | 1 | 30 | 0 | 4.5 | 4.8 | 4.5 | 7 | 0.21 | 5 | 0 | 4.5 | 4.8 | 4.8 | 4.4 | 4.5 | 4.7 | 4.8 | 4.5 |
| 181 | 0 | 0 | 0 | 73622 | 1 | 0 | 240 | 8 | 346300 | 5 | 1 | 5647 | 1590 | 3 | 15 | 1 | 30 | 0 | 4.5 | 4.4 | 4.7 | 10 | 0.29 | 5 | 0 | 4.8 | 4.5 | 4.4 | 4.6 | 4.8 | 4.5 | 4.5 | 4.8 |
| 182 | 0 | 0 | 0 | 48325 | 1 | 0 | 220 | 20 | 1000000 | 5 | 1 | 4389 | 2344 | 2 | 2 | 0 | 14 | 1 | 4.5 | 4.8 | 4.8 | 5 | 0.31 | 5 | 0 | 4.8 | 4.2 | 4.2 | 4.5 | 4.4 | 4.5 | 4.7 | 4.5 |
| 183 | 0 | 0 | 0 | 60337 | 1 | 0 | 285 | 10 | 80000 | 5 | 1 | 4004 | 526 | 2 | 4 | 2 | 30 | 4 | 4.4 | 4.4 | 4.5 | 3 | 0.52 | 5 | 0 | 4.4 | 4.6 | 4.5 | 4.4 | 4.5 | 4.4 | 4.5 | 4.8 |
| 184 | 0 | 0 | 0 | 15176 | 1 | 0 | 200 | 20 | 300000 | 5 | 1 | 1100 | 1572 | 1 | 3 | 0 | 30 | 3 | 4.6 | 4.4 | 4.7 | 9 | 0.30 | 5 | 0 | 4.7 | 4.2 | 4.6 | 4.7 | 4.8 | 4.7 | 4.8 | 4.8 |
| 185 | 0 | 0 | 20000 | 22646 | 1 | 0 | 180 | 5 | 150000 | 0 | 2 | 2227 | 3443 | 1 | 2 | 1 | 5 | 1 | 4.5 | 4.8 | 4.5 | 10 | 0.56 | 5 | 0 | 4.5 | 4.5 | 4.5 | 4.5 | 4.5 | 4.5 | 4.5 | 4.5 |
| 186 | 0 | 0 | 0 | 1328566 | 1 | 0 | 176.3 | 5 | 1000000 | 5 | 0 | 67609 | 58 | 5 | 15 | 0 | 0 | 0 | 4.8 | 4.6 | 4.7 | 0 | 0.00 | 5 | 0 | 4.2 | 4.4 | 4.5 | 4.5 | 4.5 | 4.5 | 4.7 | 4.4 |

**S2 Table. Raw value of indicators.**
