# Supplementary material for: A SBM-DEA based performance evaluation and optimization for social organizations participating in community and home-based elderly care services
Source: PLoS One. 2021 Mar 17;16(3):e0248474. doi: 10.1371/journal.pone.0248474 (PMC7968683; doi:10.1371/journal.pone.0248474)
Supplement: S1 Appendix — (DOCX) [file pone.0248474.s003.docx]

**S1 Appendix.**

Projections for DMUs

|  | C1 | C2 | C3 | C4 | C5 | C6 | C7 | C8 | C9 | C10 | C11 | C12 | C13 | C14 | C15 | C16 |
| --- | --- | --- | --- | --- | --- | --- | --- | --- | --- | --- | --- | --- | --- | --- | --- | --- |
| 001 | 100000.00 | 0.00 | 0.00 | 2254454.143 | 30.10 | 0.00 | 800.00 | 10.00 | 50000.00 | 11.02 | 21.97 | 117841.09 | 190375.69 | 2.00 | 3.63 | 5.81 |
| 002 | 56030.74 | 0.00 | 0.00 | 2670031.3 | 36.85 | 0.00 | 1000.00 | 5.00 | 39600.64 | 23.47 | 102.60 | 158544.94 | 253789.14 | 1.00 | 2.00 | 2.28 |
| 003 | 92042.62 | 0.00 | 0.00 | 421144.3418 | 5.81 | 0.00 | 360.00 | 9.59 | 138658.57 | 9.90 | 33.42 | 30396.62 | 50584.09 | 1.00 | 3.00 | 1.00 |
| 004 | 23000.00 | 0.00 | 0.00 | 2893433.407 | 38.91 | 0.00 | 500.00 | 4.43 | 80930.96 | 8.74 | 35.86 | 149870.00 | 268522.95 | 1.93 | 4.43 | 3.46 |
| 008 | 34482.76 | 0.00 | 0.00 | 242373.7789 | 3.57 | 0.00 | 641.64 | 10.00 | 6000.00 | 8.09 | 17.87 | 16882.84 | 30249.66 | 1.91 | 3.00 | 3.09 |
| 009 | 60000.00 | 600.00 | 1248.36 | 1389307.704 | 18.79 | 0.00 | 1200.00 | 4.49 | 24586.52 | 21.26 | 94.00 | 95063.63 | 164766.96 | 0.88 | 2.00 | 1.42 |
| 013 | 0.00 | 0.00 | 0.00 | 530228.5382 | 7.01 | 0.00 | 76.97 | 3.33 | 166666.67 | 1.74 | 2.36 | 29199.55 | 35057.45 | 1.00 | 2.00 | 0.00 |
| 014 | 0.00 | 0.00 | 0.00 | 559875.7718 | 7.52 | 0.00 | 120.00 | 2.62 | 50000.00 | 1.08 | 3.11 | 29513.41 | 37613.72 | 1.05 | 6.00 | 1.22 |
| 015 | 60000.00 | 0.00 | 0.00 | 557450.7438 | 8.22 | 0.00 | 300.00 | 4.11 | 60000.00 | 14.87 | 45.86 | 37535.77 | 65012.24 | 2.00 | 5.00 | 6.08 |
| 019 | 41227.61 | 0.00 | 0.00 | 564263.1921 | 7.22 | 0.00 | 300.00 | 3.52 | 20000.00 | 9.96 | 34.44 | 34513.88 | 54929.62 | 1.00 | 4.00 | 2.32 |
| 020 | 0.00 | 0.00 | 0.00 | 417278.4512 | 4.16 | 0.00 | 300.00 | 3.30 | 18664.11 | 6.97 | 18.05 | 33007.63 | 47796.04 | 1.00 | 3.00 | 1.00 |
| 021 | 0.00 | 0.00 | 0.00 | 395465.2855 | 4.92 | 0.38 | 100.00 | 1.93 | 1000.00 | 1.65 | 5.37 | 22138.94 | 27297.38 | 0.63 | 1.00 | 1.29 |
| 023 | 62938.60 | 0.00 | 0.00 | 976755.4386 | 13.46 | 0.00 | 700.00 | 5.00 | 20000.00 | 20.56 | 72.63 | 66918.56 | 106987.81 | 2.74 | 5.00 | 4.36 |
| 025 | 60000.00 | 0.00 | 0.00 | 1752861.081 | 24.50 | 0.00 | 1200.00 | 4.86 | 60000.00 | 33.36 | 134.92 | 121768.58 | 208952.54 | 2.00 | 12.00 | 6.65 |
| 026 | 0.00 | 0.00 | 0.00 | 1606723.99 | 18.95 | 0.00 | 1000.00 | 3.77 | 6342.11 | 22.43 | 94.24 | 108407.99 | 195276.79 | 1.00 | 2.00 | 7.59 |
| 029 | 100000.00 | 0.00 | 0.00 | 282387.2122 | 3.95 | 0.00 | 600.00 | 5.00 | 66607.98 | 7.22 | 23.22 | 19871.57 | 34216.56 | 0.78 | 2.00 | 1.25 |
| 033 | 13048.16 | 0.00 | 0.00 | 174034.6334 | 1.86 | 0.00 | 240.00 | 7.58 | 1000.00 | 5.78 | 6.65 | 11625.41 | 11997.23 | 1.00 | 2.00 | 2.00 |
| 034 | 0.00 | 0.00 | 0.00 | 87735.62046 | 1.37 | 0.00 | 200.00 | 4.14 | 1000.00 | 2.99 | 5.06 | 7951.92 | 10089.86 | 0.97 | 10.00 | 1.68 |
| 035 | 77890.26 | 0.00 | 0.00 | 238304.1863 | 7.93 | 0.00 | 200.00 | 8.00 | 5000.00 | 7.68 | 8.90 | 14502.90 | 19264.41 | 1.91 | 2.68 | 2.00 |
| 037 | 0.00 | 0.00 | 0.00 | 109471.924 | 3.20 | 0.00 | 140.00 | 4.91 | 0.00 | 3.39 | 5.65 | 7393.86 | 12609.20 | 0.49 | 0.98 | 1.47 |
| 043 | 1538140.00 | 0.00 | 0.00 | 1459856.363 | 34.49 | 0.00 | 8000.00 | 149.46 | 394703.40 | 21.42 | 45.84 | 78102.68 | 81969.91 | 8.20 | 8.57 | 1.00 |
| 044 | 30000.00 | 0.00 | 0.00 | 258873.2524 | 3.33 | 0.00 | 220.00 | 10.00 | 107147.73 | 6.51 | 11.17 | 18272.07 | 32868.93 | 3.00 | 8.00 | 2.00 |
| 045 | 0.00 | 0.00 | 0.00 | 309620.0324 | 2.19 | 0.00 | 180.00 | 4.53 | 4516.13 | 6.56 | 2.98 | 22832.37 | 7859.82 | 1.65 | 3.00 | 1.00 |
| 047 | 30000.00 | 0.00 | 0.00 | 137298.5395 | 1.19 | 0.00 | 120.00 | 7.53 | 73574.32 | 5.62 | 5.62 | 12398.54 | 14766.24 | 2.08 | 5.00 | 2.00 |
| 049 | 30000.00 | 0.00 | 0.00 | 196256.8308 | 1.32 | 0.00 | 120.00 | 6.41 | 59527.32 | 6.08 | 5.42 | 19280.80 | 19187.47 | 1.46 | 4.00 | 2.00 |
| 050 | 0.00 | 0.00 | 0.00 | 289098.9711 | 1.37 | 0.04 | 120.00 | 3.99 | 8829.57 | 5.09 | 5.09 | 24135.92 | 8511.36 | 0.97 | 10.00 | 2.00 |
| 051 | 0.00 | 0.00 | 0.00 | 319690.3667 | 1.60 | 0.00 | 120.00 | 3.89 | 7233.33 | 5.29 | 5.29 | 26963.43 | 8352.31 | 0.92 | 3.49 | 1.80 |
| 052 | 0.00 | 0.00 | 0.00 | 350254.1137 | 1.33 | 0.00 | 125.00 | 5.00 | 1187.21 | 6.38 | 6.38 | 25211.23 | 39151.73 | 0.88 | 16.26 | 8.00 |
| 053 | 0.00 | 0.00 | 0.00 | 0 | 0.00 | 0.00 | 0.00 | 0.00 | 0.00 | 0.00 | 0.00 | 0.00 | 0.00 | 0.00 | 0.00 | 0.00 |
| 054 | 0.00 | 0.00 | 0.00 | 950194.4579 | 12.88 | 0.00 | 60.00 | 0.00 | 0.00 | 1.20 | 6.00 | 48372.00 | 69245.18 | 0.00 | 0.00 | 0.00 |
| 055 | 0.00 | 0.00 | 0.00 | 0 | 0.00 | 0.00 | 0.00 | 0.00 | 0.00 | 0.00 | 0.00 | 0.00 | 0.00 | 0.00 | 0.00 | 0.00 |
| 057 | 0.00 | 0.00 | 0.00 | 397263.4501 | 2.92 | 0.00 | 280.00 | 3.50 | 0.00 | 7.23 | 11.03 | 25352.92 | 21506.13 | 1.00 | 4.00 | 1.00 |
| 060 | 0.00 | 0.00 | 0.00 | 4708845.9 | 63.74 | 0.00 | 80.00 | 0.00 | 0.00 | 1.60 | 8.00 | 233728.00 | 327686.98 | 0.00 | 0.00 | 0.00 |
| 061 | 47128.21 | 0.00 | 0.00 | 1469990.723 | 19.87 | 0.00 | 200.00 | 5.00 | 23162.39 | 7.94 | 22.60 | 82255.87 | 109543.24 | 1.00 | 3.00 | 2.00 |
| 062 | 0.00 | 0.00 | 0.00 | 2195621.5 | 29.02 | 0.00 | 700.00 | 2.00 | 5000.00 | 15.50 | 65.50 | 132548.34 | 189556.31 | 0.50 | 2.00 | 0.00 |
| 063 | 0.00 | 0.00 | 0.00 | 789730.4617 | 5.48 | 0.00 | 120.00 | 3.93 | 0.00 | 5.23 | 11.14 | 43192.28 | 35357.45 | 0.77 | 3.00 | 7.00 |
| 067 | 0.00 | 0.00 | 0.00 | 750155.3148 | 4.23 | 0.00 | 230.00 | 4.47 | 0.00 | 7.81 | 22.07 | 44391.20 | 36574.87 | 0.89 | 3.00 | 8.00 |
| 068 | 0.00 | 0.00 | 0.00 | 608019.7358 | 4.41 | 0.00 | 300.00 | 8.00 | 20000.00 | 12.00 | 12.00 | 59400.00 | 21316.48 | 2.00 | 8.00 | 0.00 |
| 070 | 0.00 | 0.00 | 0.00 | 545538.2593 | 3.73 | 0.00 | 200.00 | 2.86 | 0.00 | 6.05 | 19.27 | 33161.00 | 32164.49 | 0.54 | 3.00 | 5.00 |
| 073 | 0.00 | 0.00 | 0.00 | 657281 | 6.10 | 0.00 | 380.00 | 6.00 | 15000.00 | 12.10 | 24.50 | 58715.90 | 40782.00 | 1.50 | 6.00 | 0.00 |
| 075 | 0.00 | 0.00 | 0.00 | 601356 | 1.40 | 0.00 | 90.00 | 5.00 | 0.00 | 5.40 | 8.00 | 33332.60 | 12530.00 | 1.00 | 3.00 | 9.00 |
| 077 | 0.00 | 0.00 | 0.00 | 706754.3148 | 3.63 | 0.00 | 200.00 | 4.47 | 0.00 | 7.21 | 19.07 | 41430.80 | 31471.87 | 0.89 | 3.00 | 8.00 |
| 079 | 0.00 | 0.00 | 0.00 | 361030 | 3.00 | 0.00 | 200.00 | 4.00 | 10000.00 | 7.00 | 11.00 | 33881.00 | 18116.00 | 1.00 | 4.00 | 0.00 |
| 080 | 0.00 | 0.00 | 0.00 | 966959.9013 | 11.64 | 0.00 | 90.00 | 3.00 | 7500.00 | 4.05 | 2.25 | 57987.90 | 56922.18 | 0.75 | 3.00 | 0.00 |
| 083 | 0.00 | 0.00 | 0.00 | 362155.1023 | 4.54 | 0.00 | 300.00 | 2.00 | 15278.02 | 6.07 | 21.87 | 26117.19 | 77086.75 | 0.72 | 1.16 | 3.65 |
| 084 | 0.00 | 0.00 | 0.00 | 121359.9882 | 1.67 | 0.00 | 55.00 | 0.00 | 0.00 | 1.10 | 5.50 | 7482.20 | 12213.22 | 0.00 | 0.00 | 0.00 |
| 086 | 77000.00 | 0.00 | 0.00 | 655143.7905 | 9.18 | 0.00 | 155.00 | 6.00 | 20000.00 | 6.67 | 22.53 | 35825.07 | 52914.98 | 0.83 | 1.67 | 2.00 |
| 088 | 0.00 | 0.00 | 0.00 | 586889 | 1.20 | 0.00 | 80.00 | 5.00 | 0.00 | 5.20 | 7.00 | 32345.80 | 10829.00 | 1.00 | 3.00 | 9.00 |
| 090 | 0.00 | 0.00 | 0.00 | 2695273.226 | 30.71 | 0.00 | 1700.00 | 13.16 | 32903.23 | 43.87 | 140.39 | 214297.61 | 236840.71 | 3.29 | 13.16 | 0.00 |
| 091 | 100000.00 | 0.00 | 0.00 | 518429.3526 | 7.16 | 0.00 | 300.00 | 9.16 | 20299.15 | 10.15 | 34.45 | 35981.01 | 48195.57 | 1.00 | 5.00 | 2.00 |
| 092 | 0.00 | 0.00 | 0.00 | 434010 | 6.00 | 0.00 | 300.00 | 0.00 | 0.00 | 6.00 | 30.00 | 29604.00 | 51030.00 | 0.00 | 0.00 | 0.00 |
| 093 | 0.00 | 0.00 | 0.00 | 405433.4349 | 3.02 | 0.00 | 200.00 | 3.49 | 60000.00 | 5.15 | 15.70 | 25672.94 | 25556.45 | 0.86 | 2.00 | 3.42 |
| 094 | 0.00 | 0.00 | 0.00 | 163651.3329 | 4.77 | 0.00 | 120.00 | 0.49 | 9867.17 | 1.61 | 8.55 | 19903.89 | 29794.19 | 0.20 | 0.49 | 0.20 |
| 095 | 57518.46 | 0.00 | 0.00 | 2319195.24 | 31.63 | 0.00 | 346.31 | 5.00 | 30000.00 | 9.25 | 33.69 | 122271.20 | 185015.73 | 1.00 | 2.15 | 1.96 |
| 098 | 68539.33 | 0.00 | 0.00 | 1328019.649 | 17.95 | 0.00 | 816.46 | 5.00 | 9733.71 | 18.31 | 76.47 | 84161.78 | 140983.49 | 1.00 | 1.45 | 1.45 |
| 099 | 0.00 | 0.00 | 0.00 | 839271.4161 | 8.89 | 0.00 | 500.00 | 6.00 | 100000.00 | 7.63 | 18.39 | 52707.76 | 98051.73 | 2.00 | 5.00 | 1.00 |
| 100 | 0.00 | 0.00 | 0.00 | 1688007.026 | 20.50 | 0.00 | 300.00 | 5.00 | 12500.00 | 9.75 | 18.75 | 104535.60 | 110771.15 | 1.25 | 5.00 | 0.00 |
| 101 | 0.00 | 0.00 | 0.00 | 1190472.119 | 13.77 | 0.00 | 300.00 | 5.00 | 12500.00 | 9.75 | 18.75 | 80072.85 | 76749.49 | 1.25 | 5.00 | 0.00 |
| 103 | 0.00 | 0.00 | 0.00 | 2081090.973 | 26.48 | 0.00 | 1500.00 | 6.00 | 181663.13 | 30.91 | 129.61 | 144834.05 | 227806.11 | 2.00 | 4.00 | 0.23 |
| 105 | 47775.00 | 0.00 | 0.00 | 308534.48 | 3.08 | 0.00 | 150.00 | 6.00 | 14625.00 | 6.62 | 13.84 | 24100.13 | 16712.79 | 1.00 | 4.00 | 1.00 |
| 106 | 36666.67 | 0.00 | 0.00 | 273641.3333 | 2.60 | 0.00 | 180.00 | 3.67 | 17000.00 | 7.93 | 14.00 | 16529.07 | 20208.33 | 1.33 | 2.00 | 2.00 |
| 107 | 59776.17 | 0.00 | 0.00 | 295608.0097 | 2.98 | 0.00 | 227.20 | 6.00 | 10000.00 | 9.47 | 13.25 | 17637.21 | 22968.65 | 1.75 | 2.40 | 2.00 |
| 109 | 48916.88 | 0.00 | 0.00 | 198912.9733 | 2.84 | 0.00 | 200.00 | 5.00 | 5863.83 | 5.63 | 16.79 | 14923.89 | 23509.94 | 0.75 | 1.22 | 1.00 |
| 111 | 50000.00 | 0.00 | 0.00 | 2544945.099 | 32.80 | 0.00 | 400.00 | 8.00 | 12636.81 | 11.37 | 30.15 | 140563.63 | 182228.01 | 1.46 | 4.00 | 2.80 |
| 112 | 50000.00 | 0.00 | 0.00 | 902302.9072 | 12.38 | 0.00 | 537.98 | 5.00 | 51257.95 | 8.69 | 21.20 | 48126.58 | 72540.03 | 1.77 | 3.29 | 4.00 |
| 113 | 109471.32 | 0.00 | 0.00 | 655978.9188 | 9.79 | 0.00 | 240.00 | 10.00 | 31046.12 | 11.83 | 23.15 | 49976.24 | 74025.15 | 2.00 | 3.00 | 5.14 |
| 118 | 20000.00 | 0.00 | 0.00 | 343574.9981 | 3.70 | 0.00 | 120.00 | 4.00 | 14292.56 | 5.70 | 7.61 | 26131.01 | 20408.79 | 1.01 | 3.00 | 1.00 |
| 119 | 30000.00 | 0.00 | 0.00 | 306373.8754 | 4.14 | 0.00 | 50.00 | 2.50 | 26650.84 | 5.22 | 11.48 | 17365.02 | 25169.43 | 0.95 | 4.00 | 3.16 |
| 122 | 110738.33 | 0.00 | 0.00 | 890929.2903 | 13.48 | 0.00 | 800.00 | 10.00 | 18986.77 | 19.35 | 74.32 | 135949.83 | 131085.15 | 1.47 | 2.00 | 2.50 |
| 123 | 38547.06 | 0.00 | 0.00 | 153163.6125 | 2.39 | 0.00 | 120.00 | 3.10 | 26483.49 | 6.51 | 17.77 | 11355.69 | 20523.96 | 1.00 | 3.00 | 2.85 |
| 124 | 50000.00 | 0.00 | 0.00 | 254629.8949 | 2.35 | 0.00 | 160.00 | 5.00 | 50000.00 | 8.29 | 18.24 | 16431.40 | 23319.04 | 1.70 | 4.31 | 3.00 |
| 125 | 80000.00 | 0.00 | 0.00 | 223804.0768 | 2.47 | 0.00 | 160.00 | 7.66 | 30000.00 | 8.13 | 9.76 | 18129.30 | 16749.19 | 1.55 | 4.00 | 2.00 |
| 126 | 50000.00 | 0.00 | 0.00 | 85892.03846 | 1.67 | 0.00 | 50.00 | 3.88 | 29487.18 | 5.43 | 12.78 | 6996.33 | 11533.37 | 1.00 | 3.00 | 3.00 |
| 130 | 30000.00 | 0.00 | 0.00 | 51043 | 1.00 | 0.00 | 30.00 | 2.00 | 30000.00 | 5.00 | 11.00 | 4220.00 | 8529.00 | 1.00 | 2.00 | 3.00 |
| 131 | 0.00 | 0.00 | 83573.76 | 660944.1079 | 7.05 | 0.00 | 440.00 | 5.00 | 26177.74 | 11.22 | 35.85 | 51543.51 | 55092.96 | 0.95 | 4.78 | 1.96 |
| 135 | 0.00 | 0.00 | 0.00 | 679117.1384 | 4.14 | 0.00 | 330.00 | 10.00 | 22920.43 | 14.20 | 11.36 | 67426.80 | 27631.64 | 2.52 | 9.52 | 2.00 |
| 139 | 0.00 | 0.00 | 0.00 | 264147 | 3.90 | 0.00 | 180.00 | 0.00 | 0.00 | 3.60 | 18.00 | 17942.70 | 31077.30 | 0.00 | 0.00 | 0.00 |
| 140 | 0.00 | 0.00 | 0.00 | 432847.0811 | 1.41 | 0.00 | 100.00 | 4.25 | 3686.07 | 5.11 | 6.13 | 28666.05 | 9603.79 | 0.92 | 3.14 | 5.00 |
| 141 | 5000.00 | 0.00 | 0.00 | 637475.2322 | 8.52 | 0.00 | 586.75 | 3.07 | 31000.00 | 8.83 | 43.02 | 42402.44 | 99731.53 | 0.97 | 2.00 | 0.97 |
| 143 | 142640.01 | 0.00 | 0.00 | 895229.5414 | 10.37 | 0.00 | 810.00 | 10.00 | 46956.94 | 10.46 | 10.02 | 60199.98 | 50299.71 | 2.00 | 6.00 | 2.00 |
| 144 | 31741.15 | 0.00 | 0.00 | 551628.1165 | 7.92 | 0.00 | 380.00 | 2.20 | 29283.64 | 11.93 | 45.57 | 38381.20 | 67440.31 | 1.00 | 2.00 | 2.95 |
| 145 | 60000.00 | 0.00 | 0.00 | 1177634.831 | 15.30 | 0.00 | 105.17 | 5.00 | 26112.00 | 7.38 | 15.18 | 57810.86 | 80242.85 | 1.75 | 2.77 | 3.00 |
| 146 | 61672.37 | 0.00 | 0.00 | 4454968.296 | 59.39 | 0.00 | 3047.00 | 8.42 | 23056.69 | 67.39 | 298.48 | 307640.48 | 497794.33 | 2.00 | 5.00 | 2.00 |
| 147 | 88060.70 | 0.00 | 0.00 | 1288337.614 | 17.79 | 0.00 | 193.03 | 10.00 | 6022.47 | 6.95 | 12.32 | 65958.07 | 90496.76 | 1.87 | 3.00 | 2.70 |
| 148 | 0.00 | 0.00 | 0.00 | 286807.7778 | 2.89 | 0.00 | 240.00 | 3.72 | 0.00 | 5.74 | 11.88 | 18808.04 | 21560.87 | 0.71 | 1.00 | 0.86 |
| 149 | 37543.50 | 0.00 | 0.00 | 281523.3142 | 3.25 | 0.00 | 200.00 | 4.45 | 18610.03 | 7.25 | 14.76 | 20247.84 | 25925.59 | 1.00 | 2.00 | 1.00 |
| 150 | 79249.99 | 0.00 | 0.00 | 1193981.24 | 88.13 | 0.00 | 284.29 | 5.00 | 98685.45 | 4.49 | 11.64 | 60425.31 | 147594.58 | 0.86 | 4.00 | 1.00 |
| 151 | 0.00 | 0.00 | 0.00 | 1363877.895 | 18.50 | 0.00 | 100.00 | 0.00 | 0.00 | 2.00 | 10.00 | 69813.90 | 100380.00 | 0.00 | 0.00 | 0.00 |
| 152 | 30969.62 | 0.00 | 0.00 | 549314.9432 | 4.80 | 0.00 | 260.00 | 10.00 | 42658.60 | 12.99 | 9.79 | 53090.13 | 11911.53 | 2.61 | 10.00 | 1.00 |
| 153 | 78334.29 | 0.00 | 0.00 | 610187.5401 | 7.51 | 0.00 | 500.00 | 10.00 | 9178.08 | 13.30 | 39.02 | 47298.18 | 59829.24 | 1.45 | 3.29 | 1.00 |
| 154 | 69581.88 | 0.00 | 0.00 | 619971.9369 | 7.65 | 0.00 | 140.00 | 5.00 | 48794.57 | 11.35 | 28.27 | 32444.45 | 46553.32 | 2.00 | 5.00 | 5.00 |
| 156 | 22140.24 | 0.00 | 0.00 | 373550.8951 | 1.86 | 0.00 | 200.00 | 5.00 | 28227.15 | 8.55 | 6.99 | 26389.65 | 7144.37 | 1.73 | 4.00 | 1.00 |
| 157 | 0.00 | 0.00 | 0.00 | 709358.4746 | 10.80 | 0.00 | 100.00 | 0.00 | 0.00 | 2.00 | 10.00 | 37615.64 | 56570.00 | 0.00 | 0.00 | 0.00 |
| 158 | 98941.40 | 0.00 | 155.36 | 351869.8599 | 5.33 | 0.00 | 270.00 | 10.00 | 10231.87 | 7.72 | 24.06 | 27653.80 | 35752.56 | 0.85 | 2.00 | 1.00 |
| 159 | 22718.69 | 0.00 | 0.00 | 358728.3905 | 3.98 | 0.00 | 240.00 | 4.27 | 14456.59 | 7.98 | 19.85 | 30149.22 | 29697.31 | 1.00 | 3.00 | 1.00 |
| 162 | 126769.23 | 0.00 | 0.00 | 285558.7769 | 5.29 | 0.00 | 175.00 | 10.00 | 58974.36 | 12.36 | 33.51 | 21875.05 | 36259.24 | 2.00 | 6.00 | 6.00 |
| 163 | 41290.19 | 0.00 | 0.00 | 159415.1248 | 1.30 | 0.00 | 110.00 | 4.05 | 22786.74 | 6.02 | 9.82 | 9454.87 | 8409.53 | 1.20 | 2.00 | 2.00 |
| 165 | 50000.00 | 0.00 | 0.00 | 1528968.294 | 21.27 | 0.00 | 100.00 | 5.08 | 5000.00 | 3.79 | 10.27 | 77206.52 | 106928.11 | 1.11 | 1.47 | 1.59 |
| 166 | 0.00 | 0.00 | 0.00 | 578365.1708 | 4.45 | 0.00 | 260.00 | 3.66 | 10000.00 | 7.44 | 22.18 | 36602.58 | 58244.89 | 0.95 | 2.00 | 6.45 |
| 167 | 0.00 | 0.00 | 0.00 | 782937.1053 | 10.64 | 0.00 | 100.00 | 0.00 | 0.00 | 2.00 | 10.00 | 41250.26 | 60655.00 | 0.00 | 0.00 | 0.00 |
| 170 | 0.00 | 0.00 | 0.00 | 560125.5456 | 5.65 | 0.00 | 370.00 | 5.00 | 31884.12 | 10.03 | 24.98 | 43554.53 | 88943.14 | 1.63 | 4.00 | 4.31 |
| 171 | 0.00 | 0.00 | 0.00 | 535594.368 | 7.18 | 0.00 | 510.00 | 5.00 | 120000.00 | 9.22 | 36.72 | 38460.08 | 60573.63 | 1.48 | 6.00 | 1.08 |
| 174 | 0.00 | 0.00 | 0.00 | 751775.2897 | 7.21 | 0.00 | 650.00 | 19.62 | 228997.27 | 11.96 | 30.68 | 54567.00 | 85432.29 | 2.00 | 8.00 | 2.00 |
| 175 | 0.00 | 0.00 | 0.00 | 419106.093 | 4.45 | 0.00 | 300.00 | 5.00 | 14237.05 | 8.06 | 19.95 | 33154.11 | 46522.15 | 0.96 | 8.11 | 2.00 |
| 176 | 0.00 | 0.00 | 0.00 | 355197.2989 | 3.59 | 0.00 | 220.00 | 3.00 | 13475.96 | 6.54 | 15.63 | 28655.13 | 48383.54 | 0.92 | 2.38 | 2.31 |
| 177 | 0.00 | 0.00 | 0.00 | 67995 | 1.00 | 0.00 | 350.00 | 5.00 | 350000.00 | 5.00 | 1.00 | 6578.00 | 3813.00 | 2.00 | 20.00 | 2.00 |
| 178 | 0.00 | 0.00 | 0.00 | 369800.0946 | 3.91 | 0.00 | 265.00 | 5.00 | 12882.28 | 7.42 | 16.97 | 29493.10 | 41271.97 | 0.98 | 5.23 | 2.00 |
| 179 | 0.00 | 0.00 | 0.00 | 363073.1543 | 3.68 | 0.00 | 450.00 | 10.00 | 211322.99 | 8.00 | 14.20 | 29050.39 | 53416.88 | 2.00 | 5.25 | 2.00 |
| 180 | 0.00 | 0.00 | 0.00 | 469481.1931 | 5.23 | 0.00 | 472.00 | 7.43 | 148361.53 | 9.62 | 24.46 | 38214.92 | 40921.53 | 2.00 | 6.00 | 1.00 |
| 181 | 0.00 | 0.00 | 0.00 | 177505.5774 | 1.25 | 0.00 | 240.00 | 8.00 | 128326.20 | 5.84 | 3.51 | 19192.24 | 4824.15 | 1.99 | 4.79 | 1.00 |
| 183 | 0.00 | 0.00 | 0.00 | 378611.249 | 1.75 | 0.00 | 285.00 | 5.47 | 19993.48 | 8.01 | 3.21 | 29083.99 | 30385.03 | 2.00 | 4.00 | 2.00 |
| 185 | 0.00 | 0.00 | 0.00 | 59131.645 | 1.26 | 0.00 | 175.41 | 4.89 | 90683.39 | 2.33 | 2.35 | 4710.19 | 11963.13 | 0.98 | 2.00 | 1.00 |
| 186 | 0.00 | 0.00 | 0.00 | 1933329.243 | 23.77 | 0.00 | 176.30 | 5.00 | 12500.00 | 7.28 | 6.38 | 113189.79 | 118742.18 | 1.25 | 5.00 | 0.00 |

Projections for DMUs

|  | C17 | C18 | C19 | C20 | C21 | C22 | C23 | C24 | C25 | C26 | C27 | C28 | C29 | C30 | C31 | C32 | C33 |
| --- | --- | --- | --- | --- | --- | --- | --- | --- | --- | --- | --- | --- | --- | --- | --- | --- | --- |
| 001 | 27.21 | 2.39 | 136.51 | 136.44 | 129.52 | 81.57 | 0.67 | 152.62 | 0.00 | 145.46 | 138.34 | 133.91 | 137.08 | 145.75 | 139.70 | 137.83 | 136.92 |
| 002 | 8.31 | 0.41 | 160.35 | 160.37 | 159.06 | 28.16 | 1.41 | 184.26 | 0.00 | 172.91 | 171.49 | 158.44 | 164.03 | 172.91 | 165.68 | 166.02 | 162.19 |
| 003 | 18.98 | 2.77 | 24.70 | 24.70 | 25.96 | 89.10 | 0.74 | 29.04 | 0.00 | 26.71 | 27.57 | 24.98 | 25.84 | 26.74 | 25.75 | 26.16 | 25.46 |
| 004 | 28.37 | 3.48 | 173.81 | 173.70 | 165.13 | 47.76 | 3.73 | 194.57 | 0.00 | 185.23 | 176.68 | 170.14 | 174.60 | 185.65 | 177.64 | 175.28 | 174.24 |
| 008 | 20.69 | 0.00 | 15.92 | 15.86 | 16.77 | 45.29 | 0.48 | 18.72 | 0.00 | 17.11 | 17.67 | 16.10 | 16.51 | 17.35 | 16.50 | 17.09 | 16.44 |
| 009 | 7.10 | 2.00 | 79.38 | 79.38 | 83.11 | 187.04 | 0.17 | 94.27 | 0.00 | 86.75 | 90.34 | 79.47 | 83.05 | 86.75 | 83.12 | 84.93 | 81.22 |
| 013 | 1.67 | 0.00 | 31.51 | 31.58 | 29.45 | 0.00 | 0.26 | 35.04 | 0.00 | 33.52 | 31.56 | 30.89 | 31.63 | 33.52 | 32.19 | 31.54 | 31.49 |
| 014 | 6.47 | 0.00 | 33.78 | 33.81 | 31.80 | 0.56 | 0.34 | 37.58 | 0.00 | 35.88 | 33.87 | 33.11 | 33.89 | 36.00 | 34.51 | 33.92 | 33.82 |
| 015 | 6.70 | 0.00 | 35.58 | 35.58 | 36.89 | 46.00 | 1.24 | 41.11 | 0.00 | 37.91 | 38.82 | 34.84 | 36.33 | 38.13 | 36.48 | 37.60 | 36.06 |
| 019 | 5.90 | 1.08 | 31.04 | 31.11 | 31.62 | 7.26 | 1.22 | 36.08 | 0.00 | 33.48 | 34.09 | 30.79 | 32.02 | 33.39 | 32.12 | 32.66 | 31.71 |
| 020 | 10.00 | 1.51 | 17.79 | 17.74 | 18.65 | 7.16 | 0.75 | 20.80 | 0.00 | 18.98 | 19.72 | 17.71 | 18.39 | 19.23 | 18.33 | 18.94 | 18.09 |
| 021 | 1.88 | 1.20 | 21.95 | 21.92 | 20.90 | 5.37 | 0.19 | 24.60 | 0.00 | 23.46 | 22.39 | 21.59 | 22.13 | 23.44 | 22.52 | 22.12 | 22.06 |
| 023 | 7.74 | 0.00 | 57.00 | 57.05 | 59.85 | 12.39 | 0.73 | 67.28 | 0.00 | 61.72 | 64.12 | 57.00 | 59.50 | 61.75 | 59.35 | 60.84 | 58.51 |
| 025 | 11.62 | 0.00 | 103.53 | 103.55 | 108.79 | 46.00 | 1.36 | 122.49 | 0.00 | 112.44 | 117.32 | 102.95 | 107.81 | 112.75 | 107.81 | 110.82 | 105.82 |
| 026 | 28.41 | 3.65 | 79.82 | 79.89 | 83.57 | 4.47 | 0.36 | 94.77 | 0.00 | 86.94 | 90.83 | 80.02 | 83.76 | 87.06 | 83.38 | 85.51 | 81.75 |
| 029 | 22.89 | 1.01 | 18.18 | 18.18 | 19.11 | 67.90 | 0.51 | 21.37 | 0.00 | 19.62 | 20.32 | 18.15 | 18.91 | 19.76 | 18.91 | 19.36 | 18.63 |
| 033 | 1.72 | 0.28 | 8.26 | 7.99 | 8.58 | 17.35 | 0.27 | 9.37 | 0.00 | 8.50 | 8.64 | 8.21 | 8.22 | 8.55 | 8.41 | 8.63 | 8.25 |
| 034 | 15.67 | 0.00 | 5.90 | 6.13 | 6.23 | 12.64 | 0.43 | 6.84 | 0.00 | 6.02 | 6.29 | 6.20 | 6.35 | 6.20 | 5.89 | 6.17 | 6.23 |
| 035 | 11.62 | 0.33 | 34.55 | 35.83 | 36.56 | 2.83 | 2.07 | 39.65 | 0.00 | 34.38 | 35.69 | 36.96 | 37.77 | 33.59 | 33.77 | 35.76 | 35.80 |
| 037 | 0.00 | 0.00 | 13.78 | 13.99 | 14.60 | 11.30 | 0.02 | 16.02 | 0.00 | 14.16 | 14.65 | 14.59 | 14.81 | 14.03 | 13.84 | 14.57 | 14.23 |
| 043 | 20.00 | 0.00 | 163.60 | 154.17 | 151.27 | 128.97 | 1.44 | 172.89 | 0.00 | 157.09 | 156.75 | 162.74 | 155.96 | 163.40 | 156.13 | 159.82 | 162.81 |
| 044 | 18.14 | 2.28 | 14.94 | 14.92 | 14.94 | 62.47 | 0.39 | 16.70 | 0.00 | 15.69 | 15.14 | 14.48 | 14.92 | 15.77 | 14.73 | 15.01 | 15.15 |
| 045 | 3.33 | 3.19 | 10.04 | 9.94 | 9.87 | 0.67 | 0.00 | 10.94 | 0.00 | 10.02 | 9.84 | 9.60 | 9.75 | 10.22 | 9.90 | 10.09 | 9.94 |
| 047 | 10.53 | 1.79 | 5.50 | 5.51 | 5.71 | 44.99 | 0.22 | 5.96 | 0.00 | 5.49 | 5.29 | 5.33 | 5.40 | 5.59 | 5.21 | 5.50 | 5.60 |
| 049 | 8.14 | 1.86 | 5.93 | 5.99 | 6.29 | 36.46 | 0.36 | 6.60 | 0.00 | 5.96 | 6.01 | 6.04 | 6.06 | 6.16 | 5.91 | 6.28 | 6.08 |
| 050 | 6.00 | 1.33 | 6.04 | 6.05 | 6.38 | 11.20 | 0.15 | 6.87 | 0.00 | 6.19 | 6.32 | 6.09 | 6.20 | 6.44 | 6.13 | 6.42 | 6.11 |
| 051 | 2.00 | 0.00 | 6.96 | 6.98 | 7.45 | 0.00 | 0.00 | 7.98 | 0.00 | 7.23 | 7.39 | 7.02 | 7.18 | 7.47 | 7.12 | 7.51 | 7.05 |
| 052 | 34.43 | 3.15 | 5.87 | 6.00 | 6.07 | 1.35 | 0.28 | 6.67 | 0.00 | 5.83 | 6.14 | 6.12 | 6.26 | 6.12 | 5.90 | 6.20 | 6.12 |
| 053 | 0.00 | 0.00 | 0.00 | 0.00 | 0.00 | 0.00 | 0.00 | 0.00 | 0.00 | 0.00 | 0.00 | 0.00 | 0.00 | 0.00 | 0.00 | 0.00 | 0.00 |
| 054 | 0.00 | 0.00 | 57.61 | 57.61 | 54.34 | 0.00 | 0.00 | 64.41 | 0.00 | 61.59 | 58.33 | 56.44 | 57.85 | 61.59 | 59.02 | 57.97 | 57.73 |
| 055 | 0.00 | 0.00 | 0.00 | 0.00 | 0.00 | 0.00 | 0.00 | 0.00 | 0.00 | 0.00 | 0.00 | 0.00 | 0.00 | 0.00 | 0.00 | 0.00 | 0.00 |
| 057 | 3.39 | 0.96 | 12.83 | 12.54 | 13.32 | 2.89 | 0.06 | 14.60 | 0.00 | 13.24 | 13.67 | 12.37 | 12.71 | 13.29 | 12.96 | 13.18 | 12.79 |
| 060 | 0.00 | 0.00 | 286.37 | 286.37 | 268.05 | 0.00 | 0.00 | 318.72 | 0.00 | 305.65 | 287.33 | 280.16 | 286.69 | 305.65 | 292.91 | 286.85 | 286.53 |
| 061 | 2.20 | 0.00 | 88.67 | 88.48 | 84.81 | 15.14 | 0.41 | 99.33 | 0.00 | 94.34 | 90.40 | 86.92 | 89.04 | 94.60 | 90.59 | 89.78 | 88.96 |
| 062 | 0.00 | 0.00 | 126.69 | 126.69 | 124.83 | 0.00 | 0.00 | 145.10 | 0.00 | 136.55 | 134.49 | 125.14 | 129.29 | 136.70 | 130.84 | 130.79 | 127.99 |
| 063 | 8.20 | 0.00 | 24.20 | 24.28 | 23.67 | 0.00 | 0.01 | 27.41 | 0.00 | 25.74 | 25.05 | 24.10 | 24.77 | 25.82 | 24.88 | 24.82 | 24.41 |
| 067 | 9.10 | 0.00 | 17.96 | 18.05 | 18.89 | 0.00 | 0.01 | 21.16 | 0.00 | 19.29 | 20.05 | 18.22 | 18.98 | 19.38 | 18.71 | 19.22 | 18.38 |
| 068 | 0.00 | 0.00 | 19.26 | 19.26 | 20.34 | 0.00 | 0.00 | 22.07 | 0.00 | 20.19 | 20.46 | 19.22 | 19.66 | 20.79 | 19.71 | 20.66 | 19.46 |
| 070 | 6.40 | 0.00 | 15.79 | 15.84 | 16.58 | 0.00 | 0.02 | 18.65 | 0.00 | 17.05 | 17.73 | 15.95 | 16.64 | 17.12 | 16.47 | 16.89 | 16.17 |
| 073 | 0.00 | 0.00 | 26.07 | 26.07 | 27.59 | 0.00 | 0.00 | 30.50 | 0.00 | 27.91 | 28.83 | 26.07 | 26.99 | 28.36 | 26.99 | 28.05 | 26.53 |
| 075 | 10.00 | 0.00 | 6.08 | 6.18 | 6.46 | 0.00 | 0.00 | 7.00 | 0.00 | 6.24 | 6.42 | 6.38 | 6.56 | 6.34 | 6.26 | 6.50 | 6.22 |
| 077 | 9.10 | 0.00 | 15.44 | 15.53 | 16.25 | 0.00 | 0.01 | 18.16 | 0.00 | 16.53 | 17.17 | 15.70 | 16.34 | 16.62 | 16.07 | 16.52 | 15.80 |
| 079 | 0.00 | 0.00 | 12.90 | 12.90 | 13.70 | 0.00 | 0.00 | 15.00 | 0.00 | 13.70 | 14.10 | 12.90 | 13.30 | 14.00 | 13.30 | 13.90 | 13.10 |
| 080 | 0.00 | 0.00 | 52.31 | 52.31 | 49.49 | 0.00 | 0.00 | 58.22 | 0.00 | 55.61 | 52.49 | 51.25 | 52.37 | 55.83 | 53.43 | 52.70 | 52.34 |
| 083 | 25.96 | 3.82 | 19.24 | 19.26 | 20.04 | 8.98 | 0.86 | 22.70 | 0.00 | 20.70 | 21.74 | 19.28 | 20.18 | 20.83 | 19.91 | 20.54 | 19.71 |
| 084 | 0.00 | 0.00 | 7.16 | 7.16 | 7.21 | 0.00 | 0.00 | 8.33 | 0.00 | 7.77 | 7.82 | 7.11 | 7.38 | 7.77 | 7.44 | 7.49 | 7.27 |
| 086 | 2.00 | 1.00 | 40.78 | 40.48 | 39.57 | 7.67 | 0.55 | 45.91 | 0.00 | 43.16 | 42.29 | 40.18 | 41.04 | 43.27 | 41.53 | 41.61 | 41.23 |
| 088 | 10.00 | 0.00 | 5.24 | 5.34 | 5.58 | 0.00 | 0.00 | 6.00 | 0.00 | 5.32 | 5.46 | 5.54 | 5.68 | 5.42 | 5.38 | 5.60 | 5.36 |
| 090 | 0.00 | 0.00 | 129.97 | 129.97 | 136.77 | 0.00 | 0.00 | 153.55 | 0.00 | 140.94 | 146.42 | 129.97 | 135.45 | 141.92 | 135.45 | 139.51 | 132.71 |
| 091 | 3.57 | 1.00 | 31.10 | 30.65 | 31.75 | 6.09 | 0.55 | 35.80 | 0.00 | 32.79 | 33.86 | 31.03 | 31.71 | 33.08 | 31.71 | 32.61 | 31.80 |
| 092 | 0.00 | 0.00 | 25.20 | 25.20 | 26.40 | 0.00 | 0.00 | 30.00 | 0.00 | 27.60 | 28.80 | 25.20 | 26.40 | 27.60 | 26.40 | 27.00 | 25.80 |
| 093 | 5.65 | 0.00 | 12.81 | 12.88 | 13.40 | 1.88 | 0.13 | 15.12 | 0.00 | 13.82 | 14.37 | 12.94 | 13.50 | 13.87 | 13.34 | 13.69 | 13.11 |
| 094 | 4.44 | 0.59 | 20.72 | 21.47 | 21.76 | 5.13 | 0.56 | 23.84 | 0.00 | 20.82 | 21.93 | 21.59 | 22.55 | 20.73 | 20.51 | 21.45 | 21.00 |
| 095 | 10.00 | 0.51 | 140.94 | 140.96 | 134.32 | 22.20 | 1.85 | 158.15 | 0.00 | 150.55 | 143.90 | 138.18 | 141.90 | 150.54 | 144.36 | 142.47 | 141.38 |
| 098 | 10.00 | 0.90 | 76.72 | 76.85 | 78.35 | 1.66 | 2.22 | 89.74 | 0.00 | 83.28 | 84.95 | 76.39 | 79.49 | 83.13 | 79.88 | 80.85 | 78.29 |
| 099 | 24.20 | 3.51 | 39.40 | 39.17 | 38.74 | 27.47 | 2.83 | 44.45 | 0.00 | 41.47 | 40.87 | 38.56 | 39.64 | 41.91 | 40.02 | 40.17 | 39.51 |
| 100 | 0.00 | 0.00 | 91.22 | 91.22 | 87.69 | 0.00 | 0.00 | 102.52 | 0.00 | 97.34 | 93.32 | 89.64 | 91.92 | 97.72 | 93.49 | 92.77 | 91.57 |
| 101 | 0.00 | 0.00 | 60.93 | 60.93 | 59.42 | 0.00 | 0.00 | 68.86 | 0.00 | 65.03 | 63.03 | 60.02 | 61.63 | 65.41 | 62.53 | 62.48 | 61.28 |
| 103 | 6.00 | 1.12 | 111.80 | 111.68 | 116.87 | 5.23 | 0.80 | 132.42 | 0.00 | 121.62 | 126.75 | 111.47 | 116.60 | 121.75 | 116.63 | 119.26 | 114.11 |
| 105 | 1.58 | 0.93 | 13.55 | 13.38 | 13.84 | 0.00 | 0.34 | 15.42 | 0.00 | 14.12 | 14.47 | 13.56 | 13.73 | 14.32 | 13.73 | 14.24 | 13.90 |
| 106 | 5.00 | 1.00 | 11.35 | 11.32 | 11.77 | 7.67 | 0.43 | 13.00 | 0.00 | 11.86 | 12.25 | 11.12 | 11.54 | 11.83 | 11.54 | 11.87 | 11.55 |
| 107 | 9.60 | 1.06 | 12.96 | 13.00 | 13.30 | 0.74 | 0.55 | 14.88 | 0.00 | 13.58 | 13.91 | 12.95 | 13.42 | 13.48 | 13.25 | 13.49 | 13.28 |
| 109 | 7.00 | 0.00 | 12.24 | 12.29 | 12.69 | 4.25 | 1.79 | 14.22 | 0.00 | 13.02 | 13.47 | 12.16 | 12.70 | 12.92 | 12.57 | 12.88 | 12.39 |
| 111 | 15.00 | 0.00 | 146.34 | 146.40 | 139.41 | 6.27 | 1.91 | 164.02 | 0.00 | 156.13 | 149.01 | 143.59 | 147.33 | 156.21 | 149.85 | 147.92 | 146.71 |
| 112 | 15.00 | 0.00 | 55.34 | 55.41 | 53.12 | 31.28 | 1.49 | 61.91 | 0.00 | 58.72 | 56.41 | 54.06 | 55.54 | 58.84 | 56.45 | 56.10 | 55.43 |
| 113 | 20.00 | 1.14 | 43.95 | 44.06 | 42.68 | 23.95 | 1.51 | 48.93 | 0.00 | 45.66 | 44.83 | 42.86 | 44.42 | 45.98 | 44.45 | 44.43 | 43.63 |
| 118 | 2.79 | 0.00 | 16.47 | 16.49 | 16.32 | 6.60 | 0.46 | 18.52 | 0.00 | 17.34 | 16.92 | 16.19 | 16.62 | 17.52 | 16.77 | 16.98 | 16.53 |
| 119 | 4.97 | 0.00 | 18.56 | 18.58 | 18.06 | 20.47 | 0.71 | 20.68 | 0.00 | 19.51 | 18.84 | 18.01 | 18.53 | 19.62 | 18.81 | 18.88 | 18.59 |
| 122 | 16.25 | 0.05 | 59.60 | 60.91 | 60.90 | 15.48 | 3.87 | 67.40 | 0.00 | 60.66 | 64.39 | 56.99 | 62.05 | 61.68 | 60.65 | 60.82 | 57.09 |
| 123 | 5.25 | 0.00 | 10.37 | 10.39 | 10.97 | 20.44 | 1.03 | 11.96 | 0.00 | 10.89 | 11.34 | 10.10 | 10.57 | 10.96 | 10.54 | 11.02 | 10.49 |
| 124 | 9.68 | 2.04 | 10.35 | 10.41 | 10.74 | 21.55 | 1.61 | 11.78 | 0.00 | 10.80 | 11.13 | 10.19 | 10.53 | 10.79 | 10.47 | 10.96 | 10.69 |
| 125 | 9.00 | 0.32 | 10.98 | 10.88 | 11.33 | 21.15 | 0.34 | 12.34 | 0.00 | 11.18 | 11.39 | 10.94 | 11.16 | 11.39 | 11.01 | 11.41 | 11.10 |
| 126 | 3.59 | 0.00 | 7.55 | 7.41 | 7.87 | 22.41 | 0.61 | 8.37 | 0.00 | 7.56 | 7.82 | 7.26 | 7.41 | 7.75 | 7.41 | 7.87 | 7.58 |
| 130 | 3.00 | 0.00 | 4.50 | 4.50 | 4.90 | 23.00 | 0.61 | 5.00 | 0.00 | 4.50 | 4.70 | 4.20 | 4.40 | 4.60 | 4.40 | 4.80 | 4.50 |
| 131 | 15.00 | 0.00 | 29.67 | 29.68 | 31.09 | 0.00 | 0.90 | 35.26 | 0.00 | 32.33 | 33.54 | 30.18 | 31.13 | 32.66 | 31.39 | 31.52 | 30.47 |
| 135 | 7.84 | 1.06 | 18.10 | 18.13 | 19.38 | 0.45 | 0.01 | 20.68 | 0.00 | 18.74 | 19.15 | 18.16 | 18.52 | 19.44 | 18.42 | 19.59 | 18.31 |
| 139 | 0.00 | 0.00 | 16.41 | 16.47 | 17.22 | 0.00 | 0.00 | 19.50 | 0.00 | 17.85 | 18.63 | 16.53 | 17.28 | 17.82 | 17.10 | 17.55 | 16.83 |
| 140 | 5.56 | 0.00 | 6.14 | 6.20 | 6.55 | 0.00 | 0.00 | 7.05 | 0.00 | 6.33 | 6.49 | 6.31 | 6.46 | 6.50 | 6.29 | 6.60 | 6.24 |
| 141 | 17.68 | 1.84 | 36.01 | 35.97 | 37.56 | 20.21 | 2.01 | 42.62 | 0.00 | 39.07 | 40.77 | 35.94 | 37.60 | 39.27 | 37.43 | 38.34 | 36.84 |
| 143 | 28.56 | 0.00 | 49.05 | 48.95 | 47.27 | 60.64 | 0.92 | 54.56 | 0.00 | 51.60 | 49.31 | 47.98 | 49.00 | 52.18 | 49.84 | 49.80 | 49.07 |
| 144 | 3.32 | 0.00 | 33.59 | 33.59 | 35.36 | 22.48 | 0.69 | 39.62 | 0.00 | 36.35 | 37.93 | 33.29 | 34.88 | 36.44 | 34.87 | 35.95 | 34.28 |
| 145 | 5.00 | 1.86 | 68.83 | 68.87 | 64.80 | 8.36 | 1.46 | 76.52 | 0.00 | 73.12 | 69.13 | 67.39 | 68.90 | 73.07 | 70.30 | 69.15 | 69.19 |
| 146 | 7.92 | 1.50 | 249.98 | 250.11 | 261.70 | 0.57 | 1.62 | 296.93 | 0.00 | 273.13 | 284.68 | 250.00 | 261.65 | 273.12 | 261.50 | 267.69 | 255.99 |
| 147 | 12.00 | 0.00 | 80.19 | 80.22 | 75.36 | 8.01 | 3.12 | 88.94 | 0.00 | 84.97 | 80.03 | 78.51 | 80.25 | 84.77 | 81.70 | 80.20 | 80.19 |
| 148 | 0.00 | 0.43 | 12.47 | 12.26 | 13.01 | 6.60 | 0.01 | 14.44 | 0.00 | 13.17 | 13.62 | 12.28 | 12.63 | 13.20 | 12.81 | 13.09 | 12.56 |
| 149 | 6.58 | 0.69 | 13.87 | 13.94 | 14.44 | 7.25 | 0.22 | 16.24 | 0.00 | 14.89 | 15.36 | 13.99 | 14.54 | 14.92 | 14.40 | 14.75 | 14.26 |
| 150 | 20.00 | 1.44 | 379.64 | 396.94 | 406.02 | 69.86 | 0.47 | 441.43 | 0.00 | 380.16 | 397.66 | 414.25 | 423.16 | 371.58 | 371.28 | 397.37 | 397.17 |
| 151 | 0.00 | 0.00 | 82.63 | 82.63 | 78.08 | 0.00 | 0.00 | 92.48 | 0.00 | 88.38 | 83.83 | 80.98 | 83.03 | 88.38 | 84.68 | 83.23 | 82.83 |
| 152 | 5.00 | 1.01 | 21.15 | 21.59 | 22.56 | 7.79 | 0.75 | 24.07 | 0.00 | 21.41 | 21.91 | 21.90 | 22.24 | 21.68 | 21.06 | 22.54 | 21.72 |
| 153 | 11.43 | 0.25 | 32.13 | 32.22 | 33.43 | 1.49 | 2.98 | 37.57 | 0.00 | 34.45 | 35.67 | 32.06 | 33.43 | 34.37 | 33.21 | 34.07 | 32.64 |
| 154 | 6.22 | 2.00 | 34.00 | 34.06 | 33.28 | 22.09 | 1.45 | 38.24 | 0.00 | 36.03 | 35.27 | 33.28 | 34.22 | 35.95 | 34.63 | 34.92 | 34.52 |
| 156 | 2.90 | 1.85 | 8.50 | 8.38 | 8.84 | 8.55 | 0.38 | 9.31 | 0.00 | 8.38 | 8.52 | 8.18 | 8.23 | 8.42 | 8.34 | 8.67 | 8.54 |
| 157 | 0.00 | 0.00 | 47.72 | 48.00 | 46.32 | 0.00 | 0.00 | 54.00 | 0.00 | 50.74 | 49.20 | 47.54 | 48.82 | 50.60 | 48.72 | 48.60 | 48.20 |
| 158 | 5.55 | 0.00 | 23.31 | 22.94 | 23.75 | 6.17 | 1.36 | 26.65 | 0.00 | 24.33 | 25.08 | 23.21 | 23.71 | 24.55 | 23.61 | 24.27 | 23.59 |
| 159 | 2.99 | 0.00 | 17.03 | 17.04 | 17.94 | 6.85 | 0.72 | 19.88 | 0.00 | 18.19 | 18.84 | 16.93 | 17.61 | 18.35 | 17.57 | 18.21 | 17.30 |
| 162 | 7.18 | 0.00 | 23.53 | 23.08 | 24.30 | 44.82 | 1.22 | 26.47 | 0.00 | 24.03 | 24.85 | 22.95 | 23.42 | 24.50 | 23.42 | 24.54 | 23.76 |
| 163 | 5.00 | 1.00 | 5.91 | 5.89 | 6.10 | 12.05 | 1.12 | 6.49 | 0.00 | 5.87 | 6.02 | 5.66 | 5.81 | 5.79 | 5.78 | 6.03 | 5.95 |
| 165 | 3.71 | 0.00 | 95.67 | 95.54 | 89.91 | 5.13 | 0.77 | 106.37 | 0.00 | 101.66 | 96.03 | 93.79 | 95.71 | 101.75 | 97.54 | 95.81 | 95.82 |
| 166 | 15.37 | 2.00 | 18.87 | 18.95 | 19.72 | 5.95 | 0.21 | 22.26 | 0.00 | 20.31 | 21.21 | 19.07 | 19.89 | 20.40 | 19.62 | 20.20 | 19.35 |
| 167 | 0.00 | 0.00 | 47.26 | 47.26 | 45.07 | 0.00 | 0.00 | 53.18 | 0.00 | 50.65 | 48.46 | 46.40 | 47.66 | 50.65 | 48.52 | 47.86 | 47.46 |
| 170 | 16.48 | 4.00 | 24.10 | 24.18 | 25.09 | 20.06 | 0.56 | 28.26 | 0.00 | 25.83 | 26.94 | 24.23 | 25.17 | 26.09 | 24.93 | 25.81 | 24.69 |
| 171 | 10.23 | 0.00 | 30.26 | 30.36 | 31.62 | 7.95 | 0.46 | 35.90 | 0.00 | 32.95 | 34.33 | 30.35 | 31.73 | 33.07 | 31.59 | 32.34 | 31.05 |
| 174 | 39.19 | 4.72 | 30.94 | 30.59 | 32.20 | 35.89 | 2.36 | 36.06 | 0.00 | 33.16 | 33.97 | 30.88 | 32.06 | 33.45 | 31.86 | 32.64 | 31.67 |
| 175 | 6.89 | 1.00 | 18.97 | 18.95 | 19.84 | 11.29 | 0.32 | 22.26 | 0.00 | 20.36 | 21.10 | 19.05 | 19.70 | 20.61 | 19.69 | 20.29 | 19.40 |
| 176 | 8.34 | 2.00 | 15.29 | 15.34 | 15.99 | 7.85 | 0.08 | 17.95 | 0.00 | 16.42 | 17.11 | 15.37 | 15.96 | 16.57 | 15.84 | 16.42 | 15.65 |
| 177 | 30.00 | 1.00 | 4.80 | 4.70 | 4.70 | 24.00 | 0.10 | 5.00 | 0.00 | 4.50 | 4.50 | 4.50 | 4.50 | 4.20 | 4.60 | 4.80 | 4.80 |
| 178 | 4.12 | 1.00 | 16.66 | 16.64 | 17.43 | 11.87 | 0.15 | 19.53 | 0.00 | 17.87 | 18.51 | 16.76 | 17.30 | 18.07 | 17.29 | 17.85 | 17.04 |
| 179 | 19.28 | 2.00 | 16.02 | 15.79 | 16.63 | 31.98 | 1.92 | 18.40 | 0.00 | 16.73 | 17.14 | 15.98 | 16.28 | 17.11 | 16.29 | 16.93 | 16.14 |
| 180 | 12.30 | 0.00 | 22.19 | 22.27 | 23.30 | 13.47 | 0.40 | 26.13 | 0.00 | 23.95 | 24.81 | 22.29 | 23.13 | 24.20 | 23.02 | 23.78 | 22.75 |
| 181 | 7.61 | 0.00 | 5.54 | 5.59 | 5.85 | 11.68 | 0.34 | 6.24 | 0.00 | 5.61 | 5.64 | 5.68 | 5.68 | 5.85 | 5.54 | 5.93 | 5.65 |
| 183 | 15.63 | 3.74 | 8.15 | 7.91 | 8.37 | 8.33 | 0.83 | 8.74 | 0.00 | 7.70 | 7.91 | 7.64 | 7.72 | 7.98 | 7.78 | 8.14 | 7.95 |
| 185 | 5.00 | 0.55 | 5.56 | 5.63 | 5.75 | 11.73 | 0.66 | 6.31 | 0.00 | 5.53 | 5.67 | 5.87 | 5.91 | 5.55 | 5.48 | 5.77 | 5.67 |
| 186 | 0.00 | 0.00 | 106.66 | 106.66 | 100.92 | 0.00 | 0.00 | 118.85 | 0.00 | 113.52 | 107.28 | 104.51 | 106.86 | 113.89 | 109.01 | 107.47 | 106.76 |
